# Relationship Between Lipohypertrophy, Glycemic Control, and Insulin Dosing: A Systematic Meta-Analysis

**DOI:** 10.1089/dia.2023.0491

**Published:** 2024-04-30

**Authors:** Julia K. Mader, Ricardo Fornengo, Ahmed Hassoun, Lutz Heinemann, Bernhard Kulzer, Magdalena Monica, Trung Nguyen, Jochen Sieber, Eric Renard, Yves Reznik, Przemysław Ryś, Anita Stożek-Tutro, Emma G. Wilmot

**Affiliations:** ^1^ Division of Endocrinology and Diabetology, Department of Internal Medicine, Medical University of Graz, Graz, Austria.; ^2^ Dipartimento di Area Medica, ASL TO4 S.S.D. di Diabetologia, Chivasso, Italy.; ^3^ Department of Medicine, Fakeeh University Hospital, Dubai, United Arab Emirates.; ^4^ Science Consulting in Diabetes GmbH, Kaarst, Germany.; ^5^ Research Institute Diabetes Academy Bad Mergentheim (FIDAM), Diabetes Center Bad Mergentheim, Bad Mergentheim, Germany.; ^6^ HTA Consulting, Cracow, Poland.; ^7^ Doctoral School of Medical and Health Sciences, Jagiellonian University Medical College, Kraków, Poland.; ^8^ embecta, Eysins, Switzerland.; ^9^ embecta, Heidelberg, Germany.; ^10^ Montpellier University Hospital and University of Montpellier, Montpellier, France.; ^11^ Endocrinology and Diabetes Department, CHU Côte de Nacre, Caen Cedex, France.; ^12^ Department of Translational Medical Sciences, University of Nottingham, Nottingham, United Kingdom.

**Keywords:** Diabetes, Glycemic Control, Insulin-injection technique, Lipohypertrophy, Meta-analysis, Systematic review

## Abstract

**Background:**

Lipohypertrophy is a common complication in patients with diabetes receiving insulin therapy. There is a lack of consensus regarding how much lipohypertrophy affects diabetes management. Our study aimed to assess the potential correlation between lipohypertrophy and glycemic control, as well as insulin dosing in patients with diabetes.

**Methods:**

We performed a systematic review followed by a meta-analysis to collect data about glycemic control and insulin dosing in diabetic patients with and without lipohypertrophy. To identify relevant studies published in English, we searched medical databases (MEDLINE/PubMed, Embase, and CENTRAL) from 1990 to January 20, 2023. An additional hand-search of references was performed to retrieve publications not indexed in medical databases. Results of meta-analyses were presented either as prevalence odds ratios (pORs) or mean differences (MDs) with 95% confidence intervals (95% CIs). This study was registered on PROSPERO (CRD42023393103).

**Results:**

Of the 5540 records and 240 full-text articles screened, 37 studies fulfilled the prespecified inclusion criteria. Performed meta-analyses showed that patients with lipohypertrophy compared with those without lipohypertrophy were more likely to experience unexplained hypoglycemia (pOR [95% CI] = 6.98 [3.30–14.77]), overall hypoglycemia (pOR [95% CI] = 6.65 [1.37–32.36]), and glycemic variability (pOR [95% CI] = 5.24 [2.68–10.23]). Patients with lipohypertrophy also had higher HbA1c (MD [95% CI] = 0.55 [0.23–0.87] %), and increased daily insulin consumption (MD [95% CI] = 7.68 IU [5.31–10.06]).

**Conclusions:**

These results suggest that overall glycemic control is worse in patients with lipohypertrophy than in those without this condition.

## Introduction

Lipohypertrophy is a common complication in patients with diabetes treated with insulin therapy.^1^ Several risk factors for developing lipohypertrophy among insulin-injecting patients have been considered, including lack of systemic rotation,^2–4^ needle reuse,^2,4,5^ needle length,^3,6,7^ and number of daily injections.^8^ The condition is primarily characterized by the enlargement of adipocytes that manifests by nodular swelling and the induration of fat tissue around the injection sites.^9^

In clinical practice, lipohypertrophy is usually diagnosed by physical examination, that is, visually and by palpation, and the most common presentation of lipohypertrophic nodules are those of large visible and esthetically displeasing mounds. However, increasing evidence suggests lipohypertrophic nodules exist in various forms, many of which are not easily visible or detectable by palpation.^10^

As physical examination methods vary between countries, as evident in the lack of uniformity in the approaches to visual and palpation examination methods, new methods of lipohypertrophy detection have emerged, including ultrasonographic skin scanning.^11,12^ A recent meta-analysis, based on data from 26,865 patients, showed that lipohypertrophy is a common health problem with a worldwide prevalence of 41.8% (95% CI: 35.9%–47.6%) among patients with diabetes.^13^ However, when considering studies that specifically utilized ultrasound sonography, these figures can rise as high as 86.5%, suggesting an underappreciated prevalence of this complication.^14,15^

Published studies suggest that many insulin-treated patients have significant deficiencies in their injection technique. They often fail to ensure proper site rotation and show a preference for injecting insulin into lipohypertrophic nodules, as these areas are less sensitive to pain.^11^ Available data indicate that insulin injections into lipohypertrophic areas may occur in up to 95.3% of patients with diabetes receiving insulin therapy.^16–18^

Lack of thorough understanding of the possible consequences of lipohypertrophy may have an unaware impact on the efficacy of antihyperglycemic therapy in individual patients. Driven by the high number of people living with diabetes and the high prevalence of lipohypertrophy, this represents an unnoticed global health problem.

Aside from the apparent esthetic influence on patients' well-being and self-image, pharmacological studies suggest that different structural properties of lipohypertrophic lesions may affect insulin absorption and metabolism.^19,20^ The insulin release from lipohypertrophic tissue is considered slower and more unpredictable than from normal fat tissue, which may result in excessive insulin dosing to achieve a pharmacological effect.^9^ However, available clinical evidence regarding the possible relationship between the presence of lipohypertrophy and glycemic control is contradictory.

Previous studies reported an increased risk of uncontrolled glycemia, glycemic variability, and episodes of unexplained hypoglycemia in patients with lipohypertrophy.^2,4,6^ For example, Gentile et al. found that 46.2% of patients with lipohypertrophy experienced one or more episodes of hypoglycemia compared with 6.8% of patients without lipohypertrophy.^16^ In contrast, other studies found no such association.^21–23^ According to Kamrul-Hasan et al., the prevalence of hypoglycemia was comparable among patients with and without lipohypertrophy.^18^ Hence, clarifying the possible link between the presence of lipohypertrophy and glycemic control is needed.

Our research aimed to critically evaluate and explain the potential relationship between lipohypertrophy and outcomes related to glycemic control (e.g., hypoglycemia events, HbA1c, and glycemic variability) and insulin dosing. We performed a systematic literature review followed by a meta-analysis to synthesize the current knowledge of this important clinical issue. We hope our results will provide clinicians with additional evidence-based information for the best management of diabetic patients and help identify critical knowledge gaps and further directions for research in this area.

## Materials and Methods

### Search strategy and selection criteria

This systematic review with a meta-analysis followed the Preferred Reporting Items for Systematic Review and Meta-Analyses (PRISMA) 2020 guidelines.^24^ We searched for studies reporting glycemic control (HbA1c, glycemic variability, uncontrolled glycemia, or continuous glycemia monitoring data), episodes of hypoglycemia (symptomatic, asymptomatic, severe, unexplained, and overall), hyperglycemia, and daily insulin doses in diabetic patients with lipohypertrophy (LH^+^) and without lipohypertrophy (LH^−^) who were treated with subcutaneous antihyperglycemic therapy administered by pens or syringes.

As therapy with glucagon-like peptide-1 (GLP-1) receptor agonists has become more prevalent in recent years, we also sought to retrieve data for the group using these antidiabetic agents in addition to insulin-treated patients. We defined glycemic variability as blood glucose oscillations <60 to >250 mg/dL at least three times a week or more than two unexplained glycemic fluctuations per week. Unexplained hypoglycemia was determined as hypoglycemic episodes without a definable precipitating event, such as a change in medication, diet, or activity. Uncontrolled glycemia included the proportion of patients with HbA1c >7.0%.

We included randomized, observational, and cross-sectional studies published in English since 1990. We justified the publication date limitation because the standard of diabetes care and insulin therapy had changed in the past decades, which could have influenced the meta-analysis results. Studies were excluded if they were conducted in patients administering insulin by nonautomated pumps or other sensor-augmented devices for continuous subcutaneous infusion. Other exclusion criteria included studies describing patients treated mainly (≥80%) with animal insulins, studies published only as conference proceedings, and studies with data presentations unsuitable for cumulation.

The systematic search was performed in MEDLINE (through PubMed), Embase, and CENTRAL (through The Cochrane Library) databases on January 20, 2023, using the keywords “diabetes” and “lipohypertrophy.” Detailed search strategies are provided in the Supplementary Appendix SA1 (Supplementary Tables S1–S3). A hand-search of references of the included studies was performed to retrieve other relevant publications not indexed in searched medical databases.

If the same research group published more than one study, we contacted the corresponding authors to ensure that studies published by the same authors do not duplicate data for the same patients. Two independent reviewers (A.S.-T. and M.M.) selected the studies according to the protocol and predefined eligibility criteria (Table 1). Any disagreements between reviewers on the full-text stage selection process were resolved by consensus.

**Table 1.** Inclusion and Exclusion Criteria

PICO	Inclusion criteria	Exclusion criteria
Population	Patients with diabetes treated with insulin or GLP-1 analogs administered by pens or syringes	Participants using insulin pumps exclusively Most of the participants (≥80%) used insulins other than humans and analogs
Intervention (exposure)	Presence of lipohypertrophy (lipodystrophy)	Not applicable
Comparator (control)	Lack of lipohypertrophy (lipodystrophy)	Not applicable
Outcomes	Glycemic control (glycemic variability, HbA1c, and CGM data), episodes of hypoglycemia and hyperglycemia (symptomatic, asymptomatic, severe, and unexplained), and daily insulin dosage	Other than defined Data presentation unsuitable for cumulation (e.g., continuous endpoints presented as medians)
Study type	Randomized clinical trials and observational studies (cohort, case–control, and cross-sectional) published in English	Studies published only as conference abstracts or posters Studies published before 1990 Studies published in languages other than English

CGM, Continuous glucose monitoring; GLP-1, glucagon-like peptide-1.

### Data analysis

Two reviewers (A.S.-T. and M.M.) performed data extraction independently. All discrepancies between reviewers were discussed and resolved. Extracted items included the design of studies, baseline population characteristics, details of antihyperglycemic therapy, analyzed outcomes (HbA1c, glycemic variability, uncontrolled glycemia, continuous glucose monitoring data, hypo/hyperglycemia, and daily insulin doses), and their definitions. The risk of bias was assessed using Joanna Briggs Institute (JBI) tools for cross-sectional^25^ and quasi-experimental^26^ studies.

We conducted meta-analyses comparing data for LH^+^ and LH^−^ only if two or more studies reported the same outcome. Results of meta-analyses were presented either as prevalence odds ratios (pORs) for the proportion of patients with an event or as mean differences (MDs) for outcomes expressed by means and standard deviations. All results were given with 95% confidence intervals (95% CIs). We used a random model (DerSimonian and Laird) for data cumulation if significant between-study heterogeneity was observed (*P*-value for Cochrane *Q* test <0.10 and *I*^2^ > 50%). In other cases, a fixed model was chosen. If available, we also extracted *P*-values for comparisons reported by authors of the individual studies.

We performed subgroup analyses to explore the effect of diabetes mellitus type, geographic region, duration of insulin therapy, and a type of lipohypertrophy measurement on meta-analyses results. We also conducted sensitivity analyses, including only studies published in the past 10 years, to determine if the publication date impacted meta-analyses results. Subgroup and sensitivity analyses were performed only for outcomes, including at least 10 studies in the primary meta-analyses. The risk of publication bias for meta-analysis of at least 10 studies was assessed by Eggers plots. For all statistical analyses, Sophie ver. 1.5.0 software was used (validated with STATA ver. 10.0).

The study was registered on the PROSPERO database (CRD42023393103).

## Results

Of the 5540 records identified during databases and references search, 240 full-text articles were assessed for eligibility, of which 200 were excluded. Finally, 37 studies described in 40 articles were included in the systematic meta-analysis (Table 2 and Supplementary Fig. S1). All excluded studies with reasons are provided in the Supplementary Appendix SA1 (Supplementary Table S4).

**Table 2.** Summary of the Included Studies

Study	Study design (location)	Number of patients (LH^+^/LH^−^)	LH measurement	Diabetes type	Population	Duration of diabetes	Duration of insulin therapy
Abujbara et al.^7^	Cross-sectional, single-center (Jordan)	851 (477/374)	Visual inspection and palpation	T1DM, T2DM	Unknown	12.3 (8.1)	7.0 (6.2)
Al Ajlouni et al.^28^	Cross-sectional, single-center (Jordan)	1090 (407/683)	Visual inspection and palpation	Only T2DM	Only adults	13.5 (9–20)^a^	4.6 (5.0)
Al Hayek et al.^2^	Cross-sectional, single-center (Saudi Arabia)	174 (83/91)	Visual inspection and palpation	Only T1DM	Only children	6.1 (4.5)	Unknown
AlJaber et al.^29^	Cross-sectional, multicenter (Saudi-Arabia)	202 (80/122)	Visual inspection and palpation	Only T2DM	Only adults	16.9 (8.5)	8.5 (5.8)
Arora et al.^30^	Cross-sectional, single-center (India)	500 (290/210)	Visual inspection and palpation, USG in all patients	T1DM, T2DM	Unknown	Unknown	3.0 (2.5–5.0)^a^
Barola et al.^3^	Cross-sectional, single-center (India)	372 (231/141)	Visual inspection and palpation	Only T1DM	Children, adults	5.6 (5.3)	Unknown
Baruah et al.^5^	Cross-sectional, single-center (India)	748 (94/654)	Visual inspection and palpation	Only T2DM	Children, adults	12.2 (7.6)	3.4 (4.2)
Blanco et al.^4^	Cross-sectional, multicenter (Spain)	430 (277/153)	Visual inspection and palpation, USG in some patients	T1DM, T2DM	Children, adults	NA (6–15)^b^	NA (1–5)^b^
Bochanen et al.^17^	Prospective, quasi-experimental, multicenter (Belgium)	146 (92/54)	Visual inspection and palpation	T1DM, T2DM	Only adults	Unknown	Unknown
Cunningham and McKenna^31^	Cross-sectional, multicenter (Ireland)	55 (28/27)	Visual inspection and palpation	T1DM, T2DM	Unknown	Unknown	15.0 (12.6)
Frid et al.^32–34^	Cross-sectional, multicenter (Worldwide)	13,289 (3855/9344)	Visual inspection and palpation	T1DM, T2DM	Children, adults	13.2 (9.7)	8.7 (8.9)
Gentile et al.^35^	Cross-sectional, multicenter (Italy)	296 (169/127)	Visual inspection and palpation	T1DM, T2DM	Only adults	7.0 (2.0)	3.0 (1.0)
Gentile et al.^36^	Cross-sectional, multicenter (Italy)	1227 (718/509)	Visual inspection and palpation, USG in all patients	Only T2DM	Only adults	10.6 (7.9)	7.6 (6.0)
Gentile et al.^6^	Cross-sectional, multicenter (Italy)	780 (360/420)	Visual inspection and palpation, USG in all patients	T1DM, T2DM	Only adults	18.0 (11.0)	10.1 (2.1)
Gentile et al.^16^	Prospective, quasi-experimental, multicenter (Italy)	1160 (487/673)	Visual inspection and palpation, USG in some patients	Only T2DM	Only adults	15.8 (7.6)	7.6 (2.2)
Gunhan et al.^37^	Cross-sectional, single-center (Turkey)	345 (98/247)	Visual inspection and palpation	Only T2DM	Only adults	37.0 (8.5)	8.9 (5.7)
Gupta et al.^38^	Cross-sectional, single-center (India)	139 (97/42)	Visual inspection and palpation	Only T2DM	Only adults	8.7 (7.5)	Unknown
Hajheydari et al.^39^	Cross-sectional, single-center (Iran)	230 (35/185)	Visual inspection and palpation	T1DM, T2DM	Unknown	14.0 (8.5)	5.4 (6.0)
Hauner et al.^40^	Cross-sectional, single-center (Germany)	279 (66/213)	Visual inspection and palpation	T1DM, T2DM	Unknown	14.1 (9.5)	Unknown
Ji and Lou^41^	Cross-sectional, multicenter (China)	380 (134/246)	Visual inspection and palpation	Only T2DM	Only adults	Unknown	3.6 (4.1)
Ji et al.^23,42^	Cross-sectional, multicenter (China)	401 (213/188)	Visual inspection and palpation	T1DM, T2DM	Only adults	11.8 (7.3)	5.8 (4.5)
Kamrul-Hasan et al.^18^	Cross-sectional, multicenter (China)	847 (78/769)	Unknown	T1DM, T2DM	Unknown	9.8 (7.0)	3.8 (4.1)
Korkmaz et al.^43^	Cross-sectional, single-center (Turkey)	136 (119/17)	Only USG	T1DM, T2DM	Only adults	15.8 (9.2)	11.4 (8.3)
Kumar et al.^44^	Cross-sectional, single-center (India)	88 (60/28)	Visual inspection and palpation, USG in all patients	T1DM, T2DM	Only adults	26.4 (5.1)	6.5 (6.6)
Lin et al.^8^	Cross-sectional, single-center (China)	120 (83/37)	Visual inspection and palpation, USG in all patients	Only T2DM	Only adults	Unknown	6.6 (4.3)
Luo et al.^45^	Cross-sectional, single-center (China)	316 (270/46)	Visual inspection and palpation, USG in some patients	T1DM, T2DM	Only adults	12.8 (16.6–19.2)^a^	6.2 (2.9–10.4)^a^
Nawaz et al.^46^	Cross-sectional, single-center (Pakistan)	363 (83/280)	Unknown	Unknown	Children, adults	7.9 (4.5)	6.5 (3.7)
Omar et al.^47^	Cross-sectional, single-center (Egypt)	119 (62/51)	Visual inspection and palpation	Only T1DM	Only children	Unknown	Unknown
Pahuja et al.^48^	Cross-sectional, single-center (India)	96 (65/31)	Visual inspection and palpation	Only T2DM	Only adults	19.8 (NA)	6.8 (NA)
Pozzuoli et al.^22^	Cross-sectional, single-center (Italy)	352 (151/201)	Visual inspection and palpation	T1DM, T2DM	Unknown	20.4 (9.9)	9.1 (8.6)
Saeed et al.^49^	Cross-sectional, single-center (Pakistan)	360 (157/203)	Visual inspection and palpation	T1DM, T2DM	Unknown	14.7 (7.6)	8.5 (6.1)
Saez de Ibarra and Gallego^50^	Cross-sectional, single-center (Spain)	150 (78/72)	Visual inspection and palpation	T1DM, T2DM	Unknown	13.0 (9.0)	11.4 (7.9)
Singha et al.^51^	Cross-sectional, single-center (India)	95 (46/45)	Visual inspection and palpation, USG in all patients	Only T1DM	Unknown	Unknown	Unknown
Strollo et al.^52^	Cross-sectional, multicenter (Italy)	387 (298/98)	Visual inspection and palpation	T1DM, T2DM	Only adults	13.0 (9.0)	10.0 (9.0)
Arda Sürücü and OKurArslan^53^	Cross-sectional, single-center (Turkey)	436 (191/245)	Visual inspection and palpation	Only T2DM	Only adults	Unknown	Unknown
Thewjitcharoen et al.^54^	Cross-sectional, single-center (Thailand)	400 (149/251)	Visual inspection and palpation, USG in all patients	T1DM, T2DM	Unknown	23.0 (10.2)	11.4 (8.7)
Tsadik et al.^21^	Cross-sectional, single-center (Ethiopia)	176 (103/73)	Visual inspection and palpation	Only T1DM	Only children	Unknown	Unknown

Continuous data are given as mean (SD) unless otherwise stated.

^a^ Median (IQR).

^b^ Median (range).

IQR, interquartile range; LH, lipohypertrophy; NA, not available; SD, standard deviation; T1DM, type 1 diabetes; T2DM, type 2 diabetes; USG, ultrasonography.

Most of the included studies were cross-sectional (35 studies) and single-center (24 studies). Only two studies^17,55^ were prospective and quasi-experimental. Five studies included only individuals with type 1 diabetes (T1DM), 11 with type 2 diabetes (T2DM), patients with either type of diabetes participated in 20 studies, and 1 study did not report information about diabetes type.^46^ Three studies^2,21,47^ focused on the pediatric population.

The size of the population in the included studies varied from 55^31^ to 13,289^9^ participants. The quality of the research was diverse based on JBI scales (4–8/8 points for cross-sectional studies and 5–6/9 points for quasi-experimental), although no study was excluded from meta-analyses due to the high risk of bias. Detailed characteristics of the included studies are presented in the Supplementary Appendix SA1 (Supplementary Tables S5–S17).

**FIG. 1.**
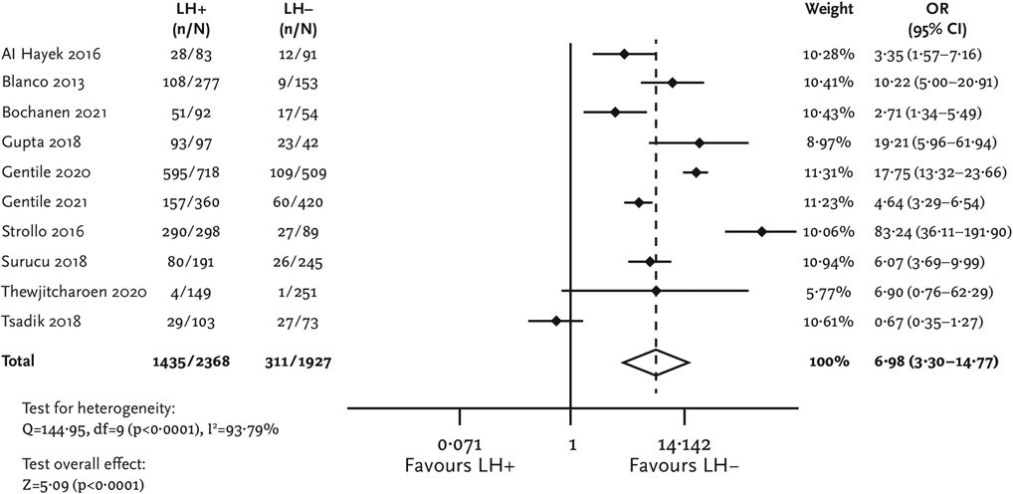
Forest plot for unexplained hypoglycemia.

**FIG. 2.**
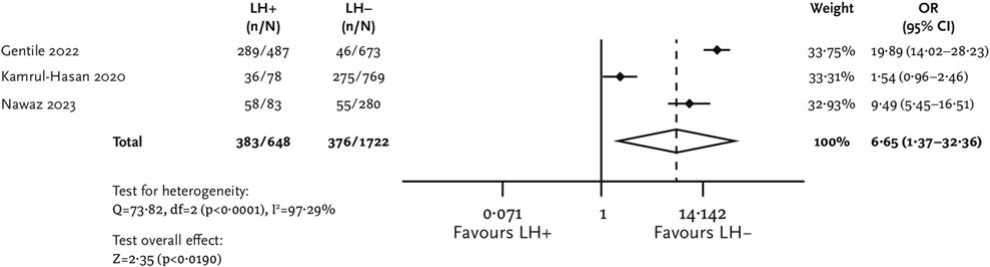
Forest plot for overall hypoglycemia.

The primary analysis showed that patients with lipohypertrophy were more likely to experience unexplained hypoglycemia (pOR [95% CI] = 6.98 [3.30–14.77]; Fig. 1) and overall hypoglycemia (pOR [95% CI] = 6.65 [1.37–32.36]; Fig. 2) compared with patients without lipohypertrophy. No between-group difference was found regarding symptomatic and severe hypoglycemia. Data for other endpoints related to hypoglycemia were presented only in individual studies, and performing meta-analyses for these outcomes was impossible.

**FIG. 3.**
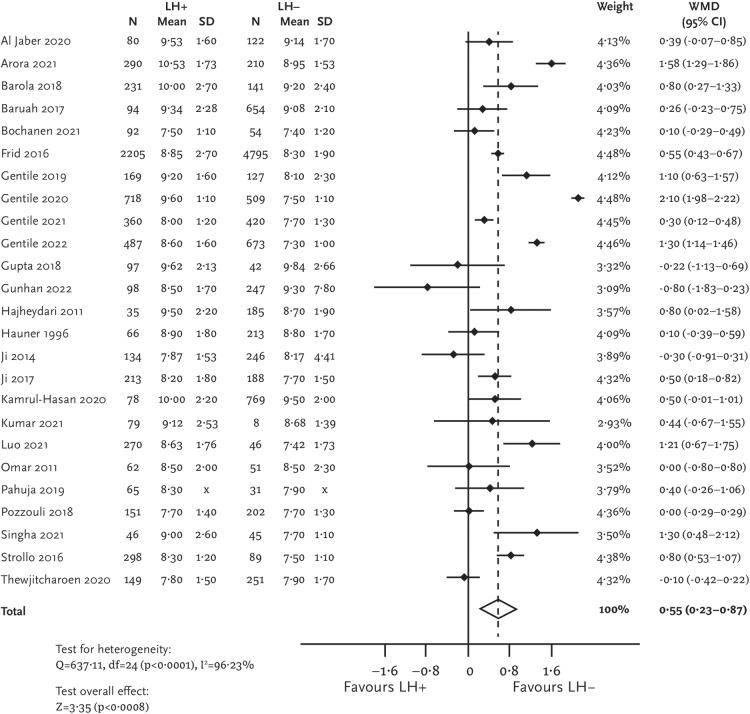
Forest plot for HbA1c.

**FIG. 4.**
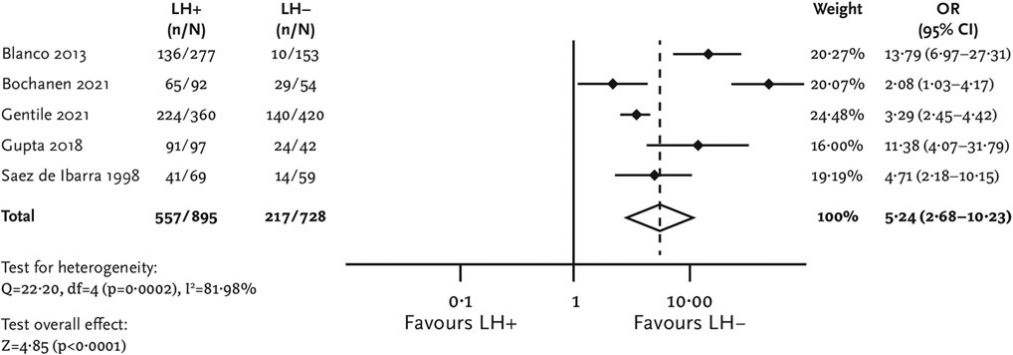
Forest plot for glycemic variability.

Patients with lipohypertrophy also had significantly higher values of HbA1c than those without lipohypertrophy (MD [95% CI] = 0.55 [0.23–0.87] %; Fig. 3). Uncontrolled glycemia, defined as HbA1c values >7%, was also more common among the lipohypertrophy group (pOR [95% CI] = 2.77 [1.62–4.73]; Fig. 4).

**FIG. 5.**
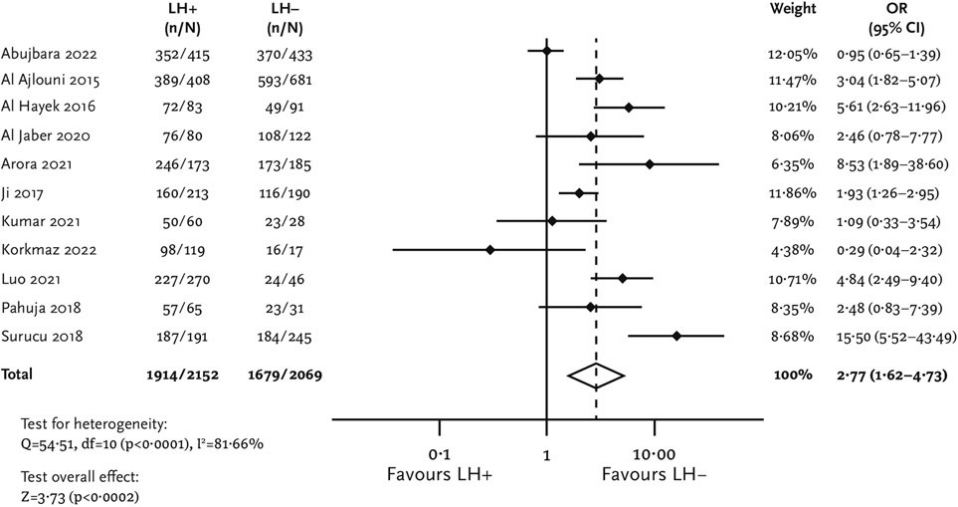
Forest plot for uncontrolled glycemia (HbA1c >7%).

The presence of lipohypertrophy was also associated with a higher prevalence of glycemic variability among patients with diabetes (pOR [95% CI] = 5.24 [2.68–10.23]; Fig. 5). Mean values of glycemic variability based on only two studies^35,36^ were higher in the lipohypertrophy group compared with the no lipohypertrophy group (MD [95% CI] = 100.20 [93.70–106.69] mg/dL).

**FIG. 6.**
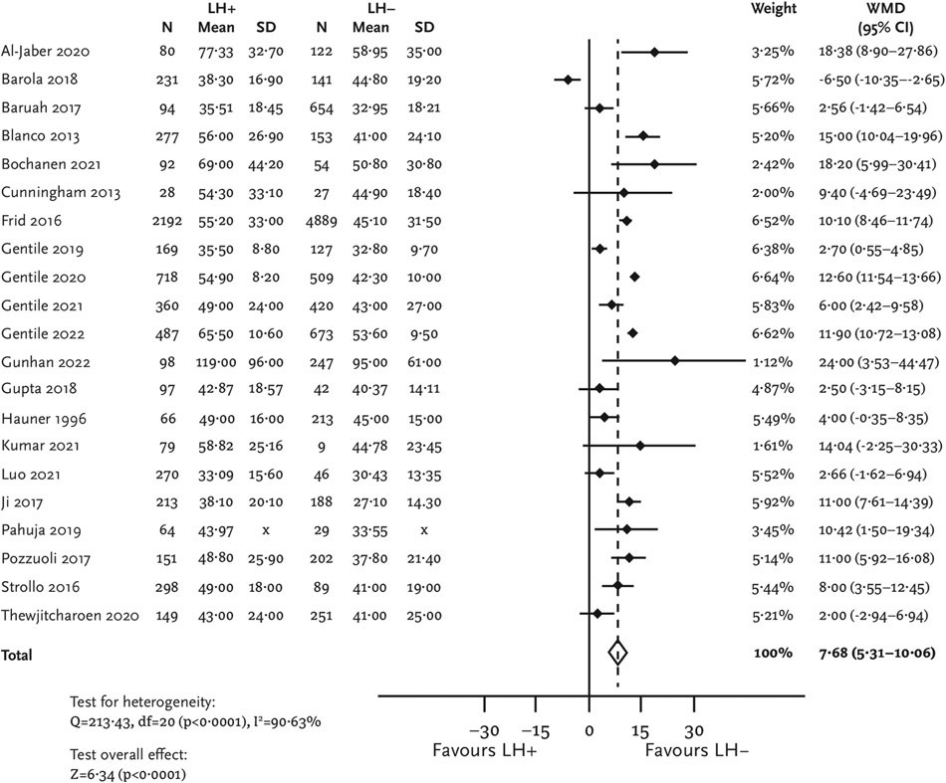
Forest plot for daily insulin dose.

Patients with lipohypertrophy were treated with higher insulin doses compared with those without lipohypertrophy (MD [95% CI] = 7.68 IU [5.31–10.06]; Fig. 6). The difference remained significant even if insulin doses were adjusted to the individuals' body weight (MD [95% CI] = 0.06 [0.01–0.12] IU/kg). Only a few identified studies reported data for hyperglycemia and continuous glucose monitoring. Results for these endpoints are given in the Supplementary Appendix SA1 (Supplementary Tables S35 and S36).

Performed sensitivity analyses indicate that the publication date did not significantly impact meta-analysis results for primary outcomes, including unexplained hypoglycemia, HbA1c, and the total daily insulin dose (Supplementary Figs. S35–S37). However, only a few identified studies were published before 2014.

Based on subgroup analyses, we noticed that the impact of lipohypertrophy on the prevalence of unexplained hypoglycemia and uncontrolled glycemia was slightly more pronounced in individuals with T2DM than in those with T1DM; however, no significant interaction between subgroups was observed (*P* > 0.05). In contrast, we reported a significant interaction for a greater impact of lipohypertrophy on the total daily insulin dose in the T2DM subgroup compared with T1DM (*P* = 0.013).

We also found that in studies reporting lipohypertrophy measured by ultrasonography, the association with unexplained hypoglycemia, HbA1c values, and total daily insulin dose was more substantial than in those with only clinical assessment of lipohypertrophy (*P* < 0.05). Regarding the geographical region, random interactions resulting from the imbalance in the number of studies in particular subgroups were noticed for unexplained hypoglycemia and the total daily insulin dose. Similarly, inconsistent results were reported for the impact of the diabetes duration. All subgroup analyses are presented in the Supplementary Appendix SA1 (Supplementary Figs. S14–S34).

No publication bias was identified for analyzed outcomes (Supplementary Figs. S38–S39).

## Discussion

Our systematic review identified 37 studies comparing glycemic control parameters and insulin dosing in patients with and without lipohypertrophy. We performed meta-analyses with a satisfactory number of studies only for four outcomes (HbA1c, uncontrolled glycemia, unexplained hypoglycemia, and total daily insulin dose). Other endpoints stated in the protocol (e.g., hyperglycemia and continuous glucose monitoring) were available in only a few studies, limiting the possibility of conducting a reliable meta-analysis. Nonetheless, we were able to collect sufficient data to determine the possible relationship between lipohypertrophy and glycemic control.

Our results showed that all primary outcomes regarding glycemic control were significantly worse in patients with lipohypertrophy than those without lipohypertrophy. Episodes of unexplained hypoglycemia, uncontrolled glycemia, and glycemic variability were more prevalent in patients with lipohypertrophy than in a control group. In addition, those with confirmed lipohypertrophy also used higher insulin doses. Although these results suggest that lipohypertrophy is associated with poorer glycemic control and higher insulin doses, we cannot draw an unequivocal causal conclusion. Nearly all of the included studies were designed as cross-sectional without any follow-up.

Therefore, we cannot rule out that there are other causal factors affecting both the development of lipohypertrophy, poor glycemic control, and higher doses of insulin. However, available data from studies evaluating the impact of educational programs on proper insulin injection techniques, with avoidance of injections into lipohypertrophy areas, indicate the direct involvement of lipohypertrophy in worsening glycemic control and excessive insulin dosing.

Wang et al.^56^ reported that a 3-month intensive training on insulin injection technique in patients with lipohypertrophy resulted in a significant and clinically relevant decrease of mean HbA1c by 0.60%, fasting plasma glucose by 1.20 mmol/L, 2h postprandial plasma glucose by 1.70 mmol/L without increasing the insulin dosage. Indicators of glycemic variability, hyperglycemic and hypoglycemic events were also markedly decreased. These meaningful results were further confirmed by a randomized controlled trial conducted by the AMD-OSDI Study Group,^57^ in which 318 patients with lipohypertrophy were assigned either to the intervention group receiving appropriate injection technique education or to the control group without education.

After a 6-month follow-up, HbA1c values, glycemic variability, and episodes of severe and symptomatic hypoglycemia were significantly decreased in the intervention group compared with the control group. In addition, insulin doses in the intervention group decreased by nearly 21%, suggesting that improvement in injection technique and its impact on lipohypertrophy allows for a reduction in insulin consumption. Notably, the benefits associated with educating patients with lipohypertrophy on injection technique are not only limited to improving health outcomes but also result in cost savings in diabetic care, including insulin costs and treatment of diabetes complications.^55^ Obtained results indicate the significance of the issue of lipohypertrophy and the necessity to adhere to FITTER guidelines regarding proper injection technique, including the importance of avoiding injections in areas affected by lipohypertrophy, proper site rotation, and needle single-use.^9^

Our research has some limitations that cannot be overcome, which result mainly from the low reliability of data pooled in meta-analyses. The studies included in this meta-analysis exhibited varying risks of bias and quality of reported outcomes. In many studies, comprehensive information on the research methodology and statistical analysis assumptions was not provided. Errors in reporting patient numbers and events also occurred,^28,52^ which made it impossible to cumulate these data for specific endpoints. Another issue observed was a high heterogeneity for almost all analyzed outcomes (*I*^2^ > 80%).

We could not establish the source of heterogeneity since the studies included in the meta-analysis were diverse in many factors simultaneously. At the same time, based on aggregated data, we could only investigate one factor in subgroup analyses. According to the protocol, we aimed to obtain a result on the effect of lipohypertrophy on glycemic control, regardless of the type of diabetes, patients' age, treatment history, and different diabetic management standards of care in various geographical regions.

In addition, differences in the definition and diagnosis of lipohypertrophy and analyzed outcomes between studies could have influenced the high heterogeneity of results. For example, in some studies, the definition of lipohypertrophy also included patients with lipoatrophy, and the diagnosis could be based on either ultrasonographic examination or solely visually and by palpation. To eliminate this potential bias, if possible, we excluded data for patients with lipoatrophy from the analysis. In other cases, patients with lipoatrophy constituted a small proportion of the entire lipohypertrophy group, so including their data should not have significantly affected the meta-analysis results.

We also performed subgroup analyses regarding the type of lipohypertrophy measurement. Interestingly, our results showed that the negative impact of lipohypertrophy on glycemic control was markedly higher in those with lipohypertrophy confirmed by ultrasound imaging compared with those with clinical assessment alone. This result may suggest that patients with subclinical lipohypertrophy, often unaware of their condition, are particularly vulnerable to glycemic fluctuations due to insulin injections into lipohypertrophy areas. At the same time, adequately educated patients with visible lipohypertrophy may avoid administering insulin into lipohypertrophic nodules.

Unfortunately, due to insufficient reporting in the studies, no analysis considering the proportion of diabetic patients with lipohypertrophy who injected insulin into affected areas could be performed. Therefore, the observed heterogeneity in our meta-analyses may also result from the inability to consider the actual percentage of patients injecting insulin into lipohypertrophy. Nonetheless, in light of these findings, it may be worth considering the introduction of routine ultrasonographic assessments in both clinical practice and trials as a more sensitive diagnostic method.

Other subgroup analyses indicate that the impact of lipohypertrophy on glycemic control was slightly more pronounced in T2DM than in those in T1DM. One possible explanation is that patients with T1DM are often better educated in injection technique and the negative consequences of lipohypertrophy due to the usual longer duration of insulin therapy. In contrast, the frequently coexisting obesity in patients with T2DM may affect the overlook of skin changes.

The pathophysiology of lipohypertrophy in patients with diabetes has not been fully elucidated, but some authors suggest that it may result not only from the lipogenic properties of insulin, promoting the growth of fat cells but also from mechanical damage to subcutaneous tissue through repeated and improper injections in the same location.^9^ The rationale for including glucagon-like peptide 1 receptor agonists (GLP-1-RAs) in the meta-analysis was based on the observation that some studies reported the occurrence of lipohypertrophy in patients using GLP-1-RA.^58^

Despite a comprehensive literature review, we found no scientific evidence regarding the potential link between lipohypertrophy and glycemic control in patients receiving GLP-1 analogs. Only one study included in the systematic review^9^ involved patients using GLP-1 receptor agonists, but they accounted for <2% of all study participants, and no subgroup analysis was available for them. Thus, there is a significant evidence gap for this patient group, and further research should focus on assessing the occurrence of lipohypertrophy and its consequences in patients treated with other than insulin subcutaneous antihyperglycemic medications.

## Conclusions

The meta-analysis results indicate that lipohypertrophy is associated with poorer glycemic control and higher insulin consumption. Clinicians and health care providers should be aware that lipohypertrophy is not only a cosmetic issue but also a clinically relevant topic. Routine screening for lipohypertrophy and intensive patient education on the proper insulin injection technique, including site rotation and needle single-use, may have a beneficial effect on better diabetes control, insulin dosing, and prevention of long-term complications of the disease.

## Supplementary Material

Supplemental data

## Supplementary Material

Supplementary Appendix SA1
