# Supplementary material for: Relationship Between Lipohypertrophy, Glycemic Control, and Insulin Dosing: A Systematic Meta-Analysis
Source: Diabetes Technol Ther. 2024 Apr 30;26(5):351–62. doi: 10.1089/dia.2023.0491 (PMC11058417; doi:10.1089/dia.2023.0491)
Supplement: Supplemental data [file Suppl_AppendixSA1.pdf]

Supplementary appendix

Supplementary tables

Table 1. MEDLINE search strategy ... 3
Table 2. EMBASE search strategy ... 3
Table 3: The Cochrane Library search strategy ... 3
Table 4. List of excluded studies with reasons ... 4
Table 5. Methodology of the included studies ... 11
Table 6. Baseline characteristics of patients in the included studies ... 13
Table 7. Characteristics of the insulin therapy in the included studies ... 18
Table 8. Detailed risk of bias in cross-sectional studies (JBI questionnaire) ... 24
Table 9. Detailed risk of bias in quasi-experimental studies (JBI questionnaire) ... 26
Table 10. Definitions of exposure (lipohypertrophy) in the included studies ... 27
Table 11. Definitions of hypoglycemia in the included studies ... 31
Table 12. Definitions of glycemic variability (GV) in the included studies ... 32
Table 13. Definitions of CGM data in the included studies ... 33
Table 14. Definitions of hyperglycemia in the included studies ... 33
Table 15. Definitions of HbA1c in the included studies ... 34
Table 16. Definitions of uncontrolled glycemia (HbA1c ≥7 or >7) in the included studies ... 34
Table 17. Definitions of daily insulin dose in the included studies ... 36
Table 18. Results in individual studies – unexplained hypoglycemia ... 37
Table 19. Results in individual studies – symptomatic hypoglycemia ... 37
Table 20. Results in individual studies – severe hypoglycemia ... 37
Table 21. Results in individual studies – overall hypoglycemia ... 37
Table 22. Results in individual studies – episodes of hypoglycemia per month ... 37
Table 23. Results in individual studies – non-symptomatic hypoglycemia ... 37
Table 24. Results in individual studies – unexplained or severe hypoglycemia ... 37
Table 25. Results in individual studies – HbA1c (%) ... 38
Table 26. Results in individual studies – uncontrolled glycemia (HbA1c >7% or ≥7%) ... 38
Table 27. Results in individual studies – glycemic variability (dich.) ... 39
Table 28. Results in individual studies – glycemic variability (mg/dL) ... 39
Table 29. Results in individual studies – daily insulin dose (insulin units) ... 39
Table 30. Results in individual studies – daily insulin dose adjusted to body weight (insulin units/kg) ... 40
Table 31. Results in individual studies – daily insulin dose >60 insulin units/day ... 40
Table 32. Results in individual studies – daily insulin dose >40 insulin units/day ... 40
Table 33. Results in individual studies – daily insulin dose >0.7 insulin units/kg/day ... 40
Table 34. Results in individual studies – daily insulin dose adjusted to HbA1c (%) ... 40
Table 35. Results in individual studies – hyperglycemia ... 40
Table 36. Results in individual studies – CGM data ... 40

Supplementary figures

Figure 1. PRISMA flow diagram ... 42
Figure 2. Forest plot for symptomatic hypoglycemia ... 43
Figure 3. Forest plot for severe hypoglycemia ... 43
Figure 4. Forest plot for episodes of hypoglycemia ... 43
Figure 5. Forest plot for glycemic variability (mg/dl) ... 43
Figure 6. Forest plot for daily insulin dose adjusted to weight (insulin units) ... 44
Figure 7. Forest plot for daily insulin dose >60 IU/day ... 44
Figure 8. Forest plot for daily insulin dose >40 IU/day ... 44
Figure 9. Forest plot for daily insulin dose >0.7 IU/kg/day ... 44
Figure 10. Forest plot for hyperglycemia ... 45
Figure 11. Forest plot for fasting plasma glucose (mg/dl) ... 45
Figure 12. Subgroup analysis for unexplained hypoglycemia – types of diabetes ... 46
Figure 13. Subgroup analysis for unexplained hypoglycemia –% of patients with T1DM ... 46
Figure 14. Subgroup analysis for unexplained hypoglycemia – geographical region (general) ... 47
Figure 15. Subgroup analysis for unexplained hypoglycemia – geographical region (including Western vs Eastern Asia) ... 47

Figure 16. Subgroup analysis for unexplained hypoglycemia – duration of diabetes in years 48
Figure 17. Subgroup analysis for unexplained hypoglycemia – a type of lipohypertrophy assessment 48
Figure 18. Subgroup analysis for HbA1c (%) – types of diabetes 49
Figure 19. Subgroup analysis for HbA1c (%) – % of patients with T1DM 50
Figure 20. Subgroup analysis for HbA1c (%) – geographical region (general) 51
Figure 21. Subgroup analysis for HbA1c (%) – geographical region (including Western vs Eastern Asia) 52
Figure 22. Subgroup analysis for HbA1c (%) – duration of diabetes in years 53
Figure 23. Subgroup analysis for HbA1c (%) – a type of lipohypertrophy measurement 54
Figure 24. Subgroup analyses for uncontrolled glycemia – types of diabetes 55
Figure 25. Subgroup analyses for uncontrolled glycemia – % of patients with T1DM 55
Figure 26. Subgroup analyses for uncontrolled glycemia – geographical region (including Western vs Eastern Asia) 56
Figure 27. Subgroup analyses for uncontrolled glycemia – duration of diabetes in years 56
Figure 28. Subgroup analyses for uncontrolled glycemia – a type of lipohypertrophy measurement 57
Figure 29. Subgroup analyses for total daily insulin dose – types of diabetes 58
Figure 30. Subgroup analyses for total daily insulin dose – % of patients with T1DM 59
Figure 31. Subgroup analyses for total daily insulin dose – geographical region (general) 60
Figure 32. Subgroup analyses for total daily insulin dose – geographical region (including Western vs Eastern Asia) 61
Figure 33. Subgroup analyses for total daily insulin dose – duration of insulin therapy 62
Figure 34. Subgroup analyses for total daily insulin dose – a type of lipohypertrophy measurement 63
Figure 35. Sensitivity analysis for unexplained hypoglycemia (studies published between 2014–2023) 64
Figure 36. Sensitivity analysis for HbA1c (%) (studies published between 2014–2023) 64
Figure 37. Sensitivity analysis for total daily insulin dose (studies published between 2014–2023) 65
Figure 38. Egger plot for publication bias – unexplained hypoglycemia 66
Figure 39. Egger plot for publication bias – uncontrolled glycemia (HbA1c >7%) 66

Supplementary tables

Table 1. MEDLINE search strategy

Index	Search terms	Hits
1	(GLP1 OR "GLP-1" OR "glucagon-like peptide 1" OR "glucagon-like peptide-1") AND (analog* OR agonist*)	11,157
2	insulin OR insulins OR insulin [MeSH]	456,962
3	#1 OR #2	463,184
4	diabetes OR diabetes mellitus [MeSH]	911,048
5	lipodystrophy OR lipodystroph* OR lipodystrophy [MeSH]	7,399
6	lipohypertrophy OR lipohypertroph* OR lipohipertrophy OR lipohipertroph*	414
7	lipoatrophy OR lipoatroph*	7,891
8	#5 OR #6 OR #7	8,303
9	#3 AND #4 AND #8	1,592

Table 2. EMBASE search strategy

Index	Search terms	Hits
1	(glp1 OR 'glp-1'/exp OR 'glp-1' OR 'glucagon-like peptide 1'/exp OR 'glucagon-like peptide 1' OR 'glucagon-like peptide-1'/exp OR 'glucagon-like peptide-1') AND (analog* OR agonist*)	20,471
2	'insulin'/exp OR insulin OR 'insulins'/exp OR insulins	942,643
3	#1 OR #2	947,642
4	'diabetes'/exp OR diabetes OR 'diabetes mellitus'/exp OR 'diabetes mellitus'	1,497,194
5	'lipodystrophy'/exp OR lipodystrophy OR lipodystroph*	14,583
6	'lipohypertrophy'/exp OR lipohypertrophy OR lipohypertroph* OR lipohipertrophy OR lipohipertroph*	1,355
7	'lipoatrophy'/exp OR lipoatrophy OR lipoatroph*	3,879
8	#5 OR #6 OR #7	17,790
9	#3 AND #4 AND #8	3,788

Table 3: The Cochrane Library search strategy

Index	Search terms	Hits
1	(GLP1 OR "GLP-1" OR "glucagon-like peptide 1" OR "glucagon-like peptide-1") AND (analog* OR agonist*)	3,016
2	MeSH descriptor: [Insulins] in all MeSH products	15,470
3	insulin OR insulins	70,095
4	#1 OR #2 OR #3	71,057
5	MeSH descriptor: [Diabetes Mellitus] explode all trees	36,048
6	diabetes OR "diabetes mellitus"	101,842
7	#5 OR #6	104,374
8	MeSH descriptor: [Lipodystrophy] explode all trees	257
9	lipodystrophy OR lipodystroph*	542
10	lipohypertrophy OR lipohypertroph* OR lipohipertrophy OR lipohipertroph*	65
11	lipoatrophy OR lipoatroph*	243
12	#8 OR #9 OR #10 OR #11	698
13	#4 AND #7 AND #12	125
14	#13 in trials	116

Table 4. List of excluded studies with reasons

PICO	Reason for exclusion	Publications
Population	All patients with lipohypertrophy (no comparison group)	1. Chen et al. Injection Technique Education in Patients with Diabetes Injecting Insulin into Areas of Lipohypertrophy: A Randomized Controlled Trial. Diabetes Ther. 2021;12(3):813-826<br>2. Famulla et al. Insulin Injection Into Lipohypertrophic Tissue: Blunted and More Variable Insulin Absorption and Action and Impaired Postprandial Glucose Control. Diabetes Care. 2016;39(9):1486-92<br>3. Gentile et al. Role of Structured Education in Reducing Lypodistrophy and its Metabolic Complications in Insulin-Treated People with Type 2 Diabetes: A Randomized Multicenter Case-Control Study. Diabetes Ther. 2021;12(5):1379-1398<br>4. Gentile et al. The Durability of an Intensive, Structured Education-Based Rehabilitation Protocol for Best Insulin Injection Practice: The ISTERP-2 Study. Diabetes Ther. 2021;12(9):2557-2569<br>5. Gentile et al. The Economic Burden of Insulin Injection-Induced Lipohypertophy. Role of Education: The ISTERP-3 Study. Adv Ther. 2022;39(5):2192-2207<br>6. Murao et al. Repeated insulin injection without site rotation affects skin thickness - ultrasonographic and histological evaluation. J Diabetes Investig. 2022;13(6):997-1003<br>7. Wang et al. Evaluation of ultrasound examination combined with intensive injection technique education on insulin-induced lipohypertrophy (LH) management: a prospective cohort study in China. International Journal of Diabetes in Developing Countries. 2021;41:669-675
Population	Congenital lipodystrophy	8. Kamrath et al. Frequency and characteristics of diabetes in lipodystrophies and insulin receptoropathies compared with type 1 and type 2: results from the multicenter DPV registry. Endocr Connect. 2023;EC-22-0333
Population	Insulin pump users	9. Al Hayek et al. Skin-Related Complications Among Adolescents With Type 1 Diabetes Using Insulin Pump Therapy. Clinical Medicine Insights: Endocrinology and Diabetes. 2018;11:<br>10. Binder et al. Frequency of dermatological side effects of continuous subcutaneous insulin infusion in children and adolescents with type 1 diabetes. Exp Clin Endocrinol Diabetes. 2015;123(4):260-4<br>11. Karlin et al. Duration of Infusion Set Survival in Lipohypertrophy Versus Nonlipohypertrophied Tissue in Patients with Type 1 Diabetes. Diabetes Technol Ther. 2016;18(7):429-35<br>12. Overland et al. Lipohypertrophy: does it matter in daily life? A study using a continuous glucose monitoring system. Diabetes Obes Metab. 2009;11(5):460-3.<br>13. Rabbone et al. Insulin pump breakdown and infusion set failure in Italian children with type 1 diabetes: A 1-year prospective observational study with suggestions to minimize clinical impact. Diabetes Obes Metab. 2018;20(11):2551-2556
Population	Lipodystrophy as underlying disease that preceded diabetes development	14. Saydam et al. Risk factors for diabetic foot ulcers in metreleptin naive patients with lipodystrophy. Clin Diabetes Endocrinol. 2021;7(1):18
Population	Mixed population – no results for multiple daily injections subgroup	15. Deeb et al. Impact of Insulin Injection and Infusion Routines on Lipohypertrophy and Glycemic Control in Children and Adults with Diabetes. Diabetes Ther. 2019;10(1):259-267<br>16. Demir et al. Local complications of insulin administration sites and effect on diabetes management. J Clin Nurs. 2022;31(17-18):2530-2538<br>17. Hernar et al. Differences in depression, treatment satisfaction and injection behaviour in adults with type 1 diabetes and different degrees of lipohypertrophy. J Clin Nurs. 2017;26(23-24):4583-4596<br>18. Lombardo et al. The Impact of Insulin-Induced Lipodystrophy on Glycemic Variability in Pediatric Patients with Type 1 Diabetes. Children (Basel). 2022;9(7):1087
Population	No information about patients with lipohypertrophy	19. Al Hayek et al. Patient-Reported Preference and Clinical Efficacy of Insulin Pen Devices With Safety Needles in Adolescents and Young Adults With Type 1 Diabetes: A Prospective Study. Cureus. 2021;13(4):e14555<br>20. Guy et al. IDF21-0662 Diabetes CarePak: Creating & testing a bundling solution to improve access to safe insulin administration in Kenya. Diabetes Research and Clinical Practice. 2022;186:<br>21. Mahoney et al. Use of a Diabetes Self-Management Application in Combination with a 4 mm Pen Needle and Its Impact on Glycemic Variability and Patient-Reported Outcomes in People with Type 2 Diabetes Using Basal-Bolus Insulin Therapy. Clinical diabetology. 2022;11:156–164<br>22. Montoya et al. Patterns of Sharps Handling and Disposal Among Insulin-Using Patients With Diabetes Mellitus. J Diabetes Sci Technol. 2021;15(1):60-66<br>23. Ranasinghe et al. Glycemic and cardiometabolic effects of exercise in South Asian Sri Lankans with type 2 diabetes mellitus: A randomized controlled trial Sri Lanka diabetes aerobic and resistance training study (SL-DARTS). Diabetes and Metabolic Syndrome: Clinical Research and Reviews. 2021;15:77-85

		24. Tsadik et al. Insulin injection practices among youngsters with diabetes in Tikur Anbesa Specialized Hospital, Ethiopia. J Diabetes Metab Disord. 2020 16;19(2):805-812<br>25. Winter et al. V-Go insulin delivery system versus multiple daily insulin injections for patients with uncontrolled type 2 diabetes mellitus. Journal of Diabetes Science and Technology. 2015;9:1111-1116
Population	Patients already described in the included Frid 2016 study	26. Dagdelen et al. Turkish Insulin Injection Techniques Study: Complications of Injecting Insulin Among Turkish Patients with Diabetes, Education They Received, and the Role of Health Care Professional as Assessed by Survey Questionnaire. Diabetes Ther. 2018;9(4):1615-1628.
Population	Patients with lipodystrophy excluded	27. Nirali et al. Prevalence of Autoantibodies in Underweight Diabetic Adult Subject in Southern Rajasthan. International Journal of Pharmaceutical and Clinical Research. 2022;14:536-542
Outcomes	Lack of defined endpoints in the study	28. Alemzadeh et al. Glucose sensor evaluation of glycemic instability in pediatric type 1 diabetes mellitus. Diabetes Technol Ther. 2003;5(2):167-73<br>29. Al-Hayek et al. Frequency and associated risk factors of recurrent diabetic ketoacidosis among Saudi adolescents with type 1 diabetes mellitus. Saudi Med J. 2015;36(2):216-20<br>30. Angamo et al. Determinants of Glycemic Control among Insulin Treated Diabetic Patients in Southwest Ethiopia: Hospital Based Cross Sectional Study. PLoS ONE. 2013;8(4): e61759<br>31. Bandari et al. Machine Learning Decision Support for Detecting Lipohypertrophy With Bedside Ultrasound: Proof-of-Concept Study. JMIR Form Res. 2022;6(5):e34830<br>32. Berard et al. Injection technique practices in a population of Canadians with diabetes: results from a recent patient/diabetes educator survey. Can J Diabetes. 2015;39(2):146-51<br>33. Bertuzzi et al. Ultrasound characterization of insulin induced lipohypertrophy in type 1 diabetes mellitus. J Endocrinol Invest. 2017;40(10):1107-1113<br>34. Calliari et al. Insulin Injection Technique Questionnaire: Results of an international study comparing Brazil, Latin America and World data. Diabetology and Metabolic Syndrome. 2018;10:85<br>35. Chlup et al. A prospective study of the hazards of multiple use of disposable syringes and needles in intensified insulin therapy. Diabet Med. 1990;7(7):624-7<br>36. Cunha et al. Insulin therapy waste produced in the households of people with diabetes monitored in Primary Care. Rev Bras Enferm. 2017;70(3):618-625<br>37. Dagdelen et al. Turkish Insulin Injection Technique Study: Population Characteristics of Turkish Patients with Diabetes Who Inject Insulin and Details of Their Injection Practices as Assessed by Survey Questionnaire. Diabetes Ther. 2018;9(4):1629-1645<br>38. De Coninck et al. Results and analysis of the 2008–2009 Insulin Injection Technique Questionnaire survey. Journal of Diabetes. 2010; 2:168–179<br>39. De Salvo et al. Effect of lipohypertrophy on accuracy of continuous glucose monitoring in patients with type 1 diabetes. Diabetes Care. 2015;38:e166-e167<br>40. De Villiers et al. Lipohypertrophy - A complication of insulin injections. South African Medical Journal. 2005;95:858-859<br>41. De Villiers et al. Lipohypertrophy - A complication of insulin injections. Journal of Endocrinology, Metabolism and Diabetes of South Africa. 2006;11:64-66<br>42. Gentile et al. A suitable palpation technique allows to identify skin lipohypertrophic lesions in insulin-treated people with diabetes. Springerplus. 2016;5:563<br>43. Gentile et al. Metabolic consequences of incorrect insulin administration techniques in aging subjects with diabetes. Acta Diabetol. 2011;48(2):121-5<br>44. Grassi et al. Optimizing insulin injection technique and its effect on blood glucose control. J Clin Transl Endocrinol. 2014;1(4):145-150<br>45. Guan et al. An analysis on the factors associated with reuse of insulin pen needles in type 2 diabetic patients in China. Journal of Chinese Pharmaceutical Sciences. 2018;27:51-58<br>46. Haslacher et al. Type 1 diabetes care: Improvement by standardization in a diabetes rehabilitation clinic. An observational report. PLoS ONE. 2018;13(3): e0194135<br>47. Hirose et al. Identification and comparison of insulin pharmacokinetics injected with a new 4-mm needle vs 6- and 8-mm needles accounting for endogenous insulin and C-peptide secretion kinetics in non-diabetic adult males. J Diabetes Investig. 2013;4(3):287-96<br>48. Kakourou et al. Limited joint mobility and lipodystrophy in children and adolescents with insulin-dependent diabetes mellitus. Pediatr Dermatol. 1994;11(4):310-4<br>49. Kalra et al. Indian Injection Technique Study: Injecting Complications, Education, and the Health Care Professional. Diabetes Ther. 2017;8(3):659-672<br>50. Kalra et al. Indian Injection Technique Study: Population Characteristics and Injection Practices. Diabetes Ther. 2017;8(3):637-657<br>51. Kalra et al. Pediatric Insulin Injection Technique: A Multi-Country Survey and Clinical Practice Implications. Diabetes Ther. 2018;9(6):2291-2302<br>52. Khutsoane et al. Biphasic insulin aspart 30 treatment improves glycaemic control in patients with type 2 diabetes in a clinical practice setting: experience from the PRESENT study. Diabetes Obes Metab. 2008;10(3):212-22.<br>53. Klimontov et al. Insulin-induced lipohypertrophy: Clinical and ultrasound characteristics. Diabetes Mellitus. 2018;21:255-263<br>54. Li et al. Injection sites lipohypertrophy among 736 patients with type 2 diabetes of

		different-grade hospitals. International Journal of Clinical and Experimental Medicine. 2016;9:13178-13183<br>55. Marran et al. SKINNY – SKIN thickness and needles in the young. SAJCH South African Journal of Child Health. 2014;8:92-95<br>56. Misnikova et al. The risks of repeated use of insulin pen needles in patients with diabetes mellitus. Journal of Diabetology. 2011;1:1-5<br>57. Nasser et al. Lipohypertrophy among insulin-treated patients. Bahrain Medical Bulletin. 2017;39(3): 146-149<br>58. Olamoyegun et al. Audit of insulin prescription patterns and associated burden among diabetics in a tertiary health institution in Nigeria. Afr Health Sci. 2018;18(4):852-864<br>59. Partanen et al. Insulin injection practices. Practical Diabetes International. 2000;17:252-254<br>60. Patil et al. Assessment of insulin injection techniques among diabetes patients in a tertiary care centre. Diabetes Metab Syndr. 2017;11(Suppl 1):S53-S56<br>61. Puder et al. Using insulin pen needles up to five times does not affect needle tip shape nor increase pain intensivity. Diabetes Research and Clinical Practice. 2005;67:119-123<br>62. Reid et al. Changing practice for safe insulin administration. Nurs Times. 2012 M;108(10):22, 24, 26<br>63. Sackey et al. Images in clinical medicine. Injection-site lipoatrophy. N Engl J Med. 2009;361(19):e41.<br>64. Schmeisl et al. Koinzidenzen: Injektionsweohnheiten, Lipohypertrophien, Glukoseschwankungen. Diabetes Stoffvy Herz. 2009;18:251-258<br>65. Shetty et al. Screening of lipohipertrophy (LH) in type I and type 2 diabetes patients and factors influencing this condition. Endocrinol Metab Int J. 2018;6(4): 309-312<br>66. Sim et al. The appropriateness of the length of insulin needles based on determination of skin and subcutaneous fat thickness in the abdomen and upper arm in patients with type 2 diabetes. Diabetes and Metabolism Journal. 2014;38:120-133<br>67. Song et al. Insulin Injection Technique in China Compared with the Rest of the World. Diabetes Ther. 2018;9(6):2357-2368<br>68. Spain et al. Self-reported Barriers to Adherence and Persistence to Treatment With Injectable Medications for Type 2 Diabetes. Clin Ther. 2016;38(7):1653-1664<br>69. Stephens et al. Subcutaneous insulin without a needle: A pilot evaluation of the J-Tip delivery system. Practical Diabetes International. 2003;20:47-50<br>70. Strauss et al. A pan-European epidemiologic study of insulin injection technique in patients with diabetes. Pract Diab Int April. 2002;19(3):71-76<br>71. Tosun et al. Do patients with diabetes use the pen insulin properly? African Health Sciences. 2019;19(1):1629–1637<br>72. Trief et al. Incorrect insulin administration: A problem that warrants attention. Clinical Diabetes. 2016;34:25-33<br>73. Tsadik et al. Insulin injection practices among youngsters with diabetes in Tikur Anbesa Specialized Hospital, Ethiopia. Journal of Diabetes and Metabolic Disorders. 2020;19(2):805-812<br>74. Vardar et al. Incidence of lipohypertrophy in diabetic patients and a study of influencing factors. Dabetes Research and Clinical Practice. 2007;77:231-236<br>75. Yu et al. Detection sensitivity of ultrasound scanning vs. clinical examination for insulin injection-related lipohypertrophy. Chin Med J (Engl). 2021;135(3):353-355
Outcomes	Lack of LH+ and LH- comparisons for defined endpoints	76. Adhikari et al. Assessment of Insulin Injection Practice of Nurses Working in a Tertiary Healthcare Center of Nepal. Nurs Res Pract. 2018;2018:9375067<br>77. Al Hayek et al. Efficacy of i-Port Advance system on patients satisfaction and glycemic control among patients with type 1 diabetes in Saudi Arabia. Diabetes and Metabolic Syndrome: Clinical Research and Reviews. 2021;15:747-751<br>78. Al Hayek et al. Evaluating the User Preference and Level of Insulin Self-Administration Adherence in Young Patients With Type 1 Diabetes: Experience With Two Insulin Pen Needle Lengths. Cureus. 2020;12(6):e8673<br>79. Anderson et al. A multicentre study of the BD (Becton Dickinson) Pen as a delivery system for human insulin. Practical Diabetes. 1994;11:36-38<br>80. Arendt-Nielsen et al. Pain following controlled cutaneous insertion of needles with different diameters. Somatosensory and Motor Research. 2006;23:37-43<br>81. Berard et al. A Coloured Pen Needle Education System Improves Insulin Site Rotation Habits: Results of a Randomized Study. Diabetes Ther. 2020;11(12):2979-2991<br>82. Bergenstal et al. Safety and efficacy of insulin therapy delivered via a 4mm pen needle in obese patients with diabetes. Mayo Clin Proc. 2015;90(3):329-38<br>83. Blanchard et al. Design and Testing of a Smartphone Application for Real-Time Tracking of CSII and CGM Site Rotation Compliance in Patients With Type 1 Diabetes. Journal of Diabetes Science and Technology. 2022;19322968221145178<br>84. Bramlage et al. A comparison of the rapid-acting insulin analogue glulisine with lispro and aspart for the pump treatment of patients with type 1 diabetes. Acta Diabetol. 2022;59(11):1453-1460<br>85. Broz et al. Lipodystrophy related to insulin injection-often overlooked disorder. Wiener Klinische Wochenschrift. 2021;133:511-512

86. Bue-Valleskey et al. Long-Term Efficacy and Safety of Ultra Rapid Lispro (URLi) in Adults with Type 1 Diabetes: the PRONTO-T1D Extension. Diabetes therapy. 2021;12:569-580
87. Campinos et al. An Effective Intervention for Diabetic Lipohypertrophy: Results of a Randomized, Controlled, Prospective Multicenter Study in France. Diabetes Technol Ther. 2017 Nov;19(11):623-632
88. De Berardis et al. Efficacy, safety and acceptability of the new pen needle 34G x 3.5 mm: a crossover randomized non-inferiority trial; AGO 02 study. Curr Med Res Opin. 2018;34(9):1699-1704.
89. Demir et al. Partial lipodystrophy of the limbs in a diabetes clinic setting. Prim Care Diabetes. 2016 Aug;10(4):293-9.
90. Dicorpo et al. Type 2 Diabetes Partitioned Polygenic Scores Associate With Disease Outcomes in 454,193 Individuals Across 13 Cohorts. Diabetes Care. 2022;45:674-683
91. Gentile et al. Lipohypertrophy in Elderly Insulin-Treated Patients With Type 2 Diabetes. Diabetes Ther. 202;12(1):107-119
92. Gorska-Ciebiada et al. Improved insulin injection technique, treatment satisfaction and glycemic control: Results from a large cohort education study. J Clin Transl Endocrinol. 2020;19:100217
93. Hacene et al. Insulin injection technique in the western region of Algeria, Tlemcen. Pan Afr Med J. 2020;36:327
94. Hansen et al. Needle with a novel attachment versus conventional screw-thread needles: a preference and usability test among adults with diabetes and impaired manual dexterity. Diabetes technology & therapeutics. 2011;13:579-585
95. Hashem et al. Characteristics and morphology of lipohypertrophic lesions in adults with type 1 diabetes with ultrasound screening: an exploratory observational study. BMJ Open Diabetes Res Care. 2021;9(2):e002553
96. Herman et al. A clinical trial of continuous subcutaneous insulin infusion versus multiple daily injections in older adults with type 2 diabetes. Diabetes care. 2005;28:1568-1573
97. Hirsch et al. Comparative glycemic control, safety and patient ratings for a new 4 mm x 32G insulin pen needle in adults with diabetes. Curr Med Res Opin. 2010;26(6):1531-41
98. Hirsch et al. Glycemic control, reported pain and leakage with a 4mm x 32G pen needle in obese and non-obese adults with diabetes: a post hoc analysis Current medical research and opinion. 2012;28:1305-1311
99. Hirsch et al. Impact of a modified needle tip geometry on penetration force as well as acceptability, preference, and perceived pain in subjects with diabetes. Journal of diabetes science and technology. 2012;6:328-335
100. Jordan et al. The Tayside insulin management course: an effective education programme in type 1 diabetes. Int J Clin Pract. 2013;67(5):462–468
101. Klarskov et al. A New Medical Device for Improved Rotation of Insulin Injections in Type 1 Diabetes Mellitus: A Proof-of-Concept Study. J Diabetes Sci Technol. 2021;15(5):1111-1120
102. Kodikara et al. Assessment of distance from skin surface to muscle for evaluation of the risk of inadvertent intramuscular insulin injection at potential injection sites among patients attending a tertiary care children's hospital in Sri Lanka-an observational study. Arch Pediatr. 2020;27(5):244-249
103. Kreugel et al. Randomized trial on the influence of the length of two insulin pen needles on glycemic control and patient preference in obese patients with diabetes. Diabetes Technol Ther. 2011;13(7):737-41
104. Liu et al. Hypoglycemia Caused by Exogenous Insulin Antibody Syndrome: A Large Single-Center Case Series from China. J Clin Endocrinol Metab. 2022;dgac578
105. Mehrabbeik et al. Investigation of Association Between Insulin Injection Technique and Blood Glucose Control in Type 2 Diabetes Patients. International Journal of Endocrinology and Metabolism. 2022;20(4):e128392
106. Misnikova et al. A Randomized Controlled Trial to Assess the Impact of Proper Insulin Injection Technique Training on Glycemic Control. Diabetes Ther. 2017 Dec;8(6):1309-1318.
107. Nagai et al. Comparison between shorter straight and thinner microtapered insulin injection needles. Diabetes technology & therapeutics. 2013;15:550-555
108. Nath et al. Assessing risk of skin lesions among people with diabetes: A case-control study from Uttarakhand, India. Iranian Journal of Dermatology. 2022;25:111-116
109. Ngo et al. Knowledge, attitude, and practice concerning hypoglycaemia, insulin use, and insulin pens in Vietnamese diabetic outpatients: Prevalence and impact on safety and disease control. Journal of Evaluation in Clinical Practice. 2021;27:404-413
110. Olamoyegun et al. Audit of insulin prescription patterns and associated burden among diabetics in a tertiary health institution in Nigeria. African Health Sciences. 2018;18:852-864
111. Praestmark et al. Pen needle design influences ease of insertion, pain, and skin trauma in subjects with type 2 diabetes. BMJ open diabetes research and care. 2016;15(4):e000266
112. Ross et al. Evaluation of 8 mm insulin pen needles in people with type 1 and type 2 diabetes. Practical diabetes international. 1999;16:145-148

		113. Saxena et al. The common pathophysiologic threads between Asian Indian diabetic's 'Thin Fat Phenotype' and partial lipodystrophy: the peripheral adipose tissue transcriptomic evidences. Adipocyte. 2020;9:253-263<br>114. Selvadurai et al. Impact of pharmacist insulin injection re-education on glycemic control among type II diabetic patients in primary health clinics. Saudi Pharm J. 2021;29(7):670-676<br>115. Sharma et al. Effect of Reuse of Insulin Needle on Glycaemic Control and Related Complications in Children with Type 1 Diabetes Mellitus: A Prospective Observational Study. Indian J Endocrinol Metab. 2022;26(2):167-172<br>116. Siegmund et al. Comparison of usability and patient preference for insulin pen needles produced with different production techniques: "thin-wall" needles compared to "regular-wall" needles: An open-label study. Diabetes Technology and Therapeutics. 2009;11:523-528<br>117. Smith et al. UK lipohypertrophy interventional study. Diabetes Res Clin Pract. 2017;126:248-253<br>118. Wang et al. Study on effect of standardized insulin injection technique on mental status and blood glucose in patients with type 2 diabetes mellitus. Acta Medica Mediterranea. 2021;37:2433-2438<br>119. Wang et al. Values of ultrasound for diagnosis and management of insulin-induced lipohypertrophy: A prospective cohort study in China. Medicine (Baltimore). 2021;100(29):e26743<br>120. Yan et al. Comparable efficacy and safety between LY2963016 insulin glargine and insulin glargine (Lantus®) in Chinese patients with type 1 diabetes: A phase III, randomized, controlled trial. Diabetes, Obesity and Metabolism. 2021;23:2226-2233<br>121. Yuan et al. Can the upper inner side of the thigh become a new option for insulin injection? Curr Med Res Opin. 2016;32(7):1319-24<br>122. Zureik et al. Prevalence, severity stages, and risk factors of diabetic retinopathy in 1464 adult patients with type 1 diabetes. Graefe's Archive for Clinical and Experimental Ophthalmology. 2021;259:3613-3623
Outcomes	Lack of possibility to compare defined endpoints	123. Schuler et al. Is the reuse of needles for insulin injection systems associated with a higher risk of cutaneous complications? Diabetes Res Clin Pract. 1992;16(3):209-12
Outcomes	Data presented only as medians	124. Van Munster et al. Dermatological complications of insulin therapy in children with type 1 diabetes. European Diabetes Nursing. 2014; 11(3):79-84
Study type	Case report	125. Barola et al. Insulin-mediated lipohypertrophy: an uncommon cause of diabetic ketoacidosis. BMJ Case Rep. 2017;2017: bcr-2017-220387<br>126. Ben Abdelkrim et al. Insulin-induced lipodystrophy and interest of insulin injection ports. Therapie. 2019;74(6):680-682<br>127. Chakraborty et al. Distant site lipoatrophy: a rare complication of subcutaneous insulin therapy. Postgrad Med J. 2016;92(1083):57-8<br>128. Chakraborty et al. Injection site lipoatrophy: a rare complication of recombinant human insulin. J Assoc Physicians India. 2010;58:630<br>129. Chakraborty et al. Nonobese, nonketotic childhood-onset diabetes: Look for Lipodystrophies. Clinical Diabetes. 2017;35:257-261<br>130. Chowdhury et al. Lesson of the week: Poor glycaemic control caused by insulin induced lipohypertrophy. British Medical Journal. 2003;327:383-384<br>131. Chowdhury et al. Poor glycaemic control caused by insulin induced lipohypertrophy. BMJ. 2003 16;327(7411):383-4.<br>132. Felner et al. Human insulin-induced lipoatrophy. J Pediatr. 2003;142(4):448<br>133. Gentile et al. How to treat improper insulin injection-related lipohypertrophy: A 3-year follow-up of a monster case and an update on treatment. Diabetes Res Clin Pract. 2021;171:108534<br>134. Harsch et al. Extent of lipohypertrophy in diabetic patients-Sometimes much more than meets the eye. Clin Case Rep. 2019;7(9):1813-1814<br>135. Holstein et al. Severe Diabetic Ketoacidosis Associated with Abdominal Lipohypertrophy. Dtsch Arztebl Int. 2021;118(40):682<br>136. Kondo et al. Insulin-Induced Distant Site Lipoatrophy. Diabetes Care. 2017;40(6):e67-e68.<br>137. Krishna et al. Insulin induced lipoatrophy. Indian Journal of Dermatology, Venereology and Leprology. 2003;69:310-311<br>138. Landau et al. Insulin-induced lipohypertrophy. New England Journal of Medicine. 2012;366:e9<br>139. Ma et al. Severe lipoatrophy with insulin in type 1 diabetes. J Dermatol. 2012;39(6):578-9<br>140. Montenegro et al. Type 2 Congenital Generalized Lipodystrophy: The Diagnosis is in Your Hands. Journal of Pediatrics. 2019;207:257-257<br>141. Page et al. Human insulin and lipoatrophy. Diabet Med. 1992;9(8):779<br>142. Velayutham et al. Lipoatrophy at insulin injection site. J Assoc Physicians India. 2008;56:335<br>143. Verma et al. Insulin-Mediated Lipohypertrophy. N Engl J Med. 2017;377(6):573

Study type	Commentary	144. Di Bartolo et al. Hundred-year experience with insulin and lipohypertrophy: An unresolved issue. Diabetes Res Clin Pract. 2021;178:108924<br>145. Gentile et al. Comment on the article Determination of insulin-related lipohypertrophy frequency and risk factors in patients with diabetes. Endocrinol Diabetes Nutr (Engl Ed). 2022;69(5):387-388<br>146. Rosenbloom et al. Insulin injection lipoatrophy recidivus. Pediatr Diabetes. 2014;15(1):73-4.
Study type	Letter to editor	147. Gentile et al. Cost saving effects of a short-term educational intervention entailing lower hypoglycaemic event rates in people with type 1 diabetes and lipo-hypertrophy. Diabetes Research and Clinical Practice. 2018;143:320-321<br>148. Kordonouri et al. Lipohypertrophy in young patients with type 1 diabetes. Diabetes care. 2002;25:634<br>149. Oriot et al. Lipohypertrophy Effect on Glycemic Profile in an Adult With Type 1 Diabetes Using Scanned Continuous Glucose Monitoring. Journal of Diabetes Science and Technology. 2019;14(2):500–501<br>150. Strauss et al. An unexpected hazard of insulin injection (multiple letters). Practical Diabetes International. 2002;19:63
Study type	Publication language other than English	151. Castro et al. Reuse of discarded syringes in residences of children and teenagers with diabetes mellitus. Rev Esc Enferm USP. 2007;41(2):187-95<br>152. Diaz et al. Lipohypertrophy in type 1 diabetes mellitus patients of the Casa de la Diabetes, Cuenca, 2017-2018. Diabetes Internacional. 2018;10:29-33<br>153. Dorchy et al. High glycated haemoglobin levels influence injection pain in diabetic children and adolescents. Revue Medicale de Bruxelles. 2008;29:5-9<br>154. Fuentes Jiménez et al. Lipodystrophies. Medicina Clinica. 2002;119:390-395<br>155. Gentile et al. Metabolic consequences of incorrect insulin administration technique: Lypohypertrophy. Giornale Italiano di Diabetologia e Metabolismo. 2010;30:97-99<br>156. Guven at al. Cutaneous manifestations in children patients with type 1 diabetes mellitus. Turkderm Turkish Archives of Dermatology and Venereology. 2021;55:22-26<br>157. Halimi et al. A persistent problem: The reuse of needles and its link with lipohypertrophies. Medecine des Maladies Metaboliques. 2018;12:516-519<br>158. Huang et al. Multimedia Health Education on Insulin Injection Skills for Patients With Type 2 Diabetes. Hu li za zhi [Journal of nursing]. 2022;69:44–54<br>159. Klimontov et al. Lipodystrophy at the insulin injection sites: Current trends in epidemiology, diagnostics and prevention. Diabetes Mellitus. 2020;23:161-173<br>160. Lazarev et al. Complex ultrasound diagnostics of insulin-induced lipohypertropthy in patients with diabetes. Russian Electronic Journal of Radiology. 2019;9:143-154<br>161. Le Floch et al. Interest of the hospital use of security needles for pen devices: The BD AutoShield™ Duo needle as an example. Medecine des Maladies Metaboliques. 2014;8:285-292<br>162. Misnikova et al. The role of proper insulin injection technique training for achieving of good glycaemic control. Diabetes Mellitus. 2018;21:419-424<br>163. Molina et al. Insulin application techneque and its relationship with lipodystrophy in patients with type I diabetes mellitus. Archivos Venezolanos de Farmacologia y Terapeutica. 2019;38:1-5<br>164. Moulin et al. Lipohypertrophy at the left flank in a right-handed type 1 diabetic patient. Medecine des Maladies Metaboliques. 2015;9:45<br>165. Mussig et al. Lipohypertrophy. Dtsch Med Wochenschr. 2006;131(33):1807-8<br>166. Sauvanet et al. Insulin injection technique: What are the practices of diabetic patients, in France. Medecine des Maladies Metaboliques. 2010;4:428-437<br>167. Sauvanetet al. Insulin injection technique: How diabetic patients proceed in France? Medecine des Maladies Metaboliques. 2017;11:406-415<br>168. Strauss et al. Does education in patients with lipohypertrophy lead to improvements in glucose control? Medecine des Maladies Metaboliques. 2017;11:425-430<br>169. Sürücü et al. Analysis of the incidence of lipohypertrophy and risk factors in the children with type 1 diabetes. Turkiye Klinikleri Pediatri. 2018;27:39-45<br>170. Teixeira et al. Reutilization of disposable syringes: frequency and costs for the administration of insulin at home. Rev Lat Am Enfermagem. 2001;9(5):47-54<br>171. Teixeira et al. Use of lancets or needles in the blood glucose self-monitoring at home. Revista brasileira de enfermagem. 2012;65:601-606<br>172. Vitebskaya et a;. Dermatological complications of insulin therapy in children with type 1 diabetes: Cross-sectional study. Voprosy Sovremennoi Pediatrii - Current Pediatrics. 2020;19:26-34<br>173. Volkova et al. Clinical significance of lipohypertrophy without visual and palpable changes detected by ultrasonography of subcutaneous fat. Ter Arkh. 2019;91(4):62-66<br>174. Yalçin et al. Correcting insulin injection technical failures in the treatment of diabetic patients, how much effective? Gazi Medical Journal. 2019;30:60-62
Study type	Review	175. Chowdhury et al. Errors of insulin therapy: Real-life experiences from developing world. J Family Med Prim Care. 2017;6(4):724-729<br>176. Clarke et al. Dose accuracy of a reusable insulin pen using a cartridge system with an integrated plunger mechanism. Expert Opinion on Drug Delivery. 2006;3:677-683

		177. Cross et al. Make a point of preventing insulin pen errors. Pharmacy Times. 2018;84(10)<br>178. Des Jardins-Park. Leveraging Mechanical Forces to Target Insulin Injection-Induced Lipohypertrophy and Fibrosis. Diabetes Spectr. 2021;34(3):308-312<br>179. Di Bartolo et al. Hundred-year experience with insulin and lipohypertrophy: An unresolved issue. Diabetes Research and Clinical Practice. 2021;178:108924.<br>180. Dorfler et al. Lipoatrophic diabetes. Clin Investig. 1993;71(4):264-9.<br>181. Gentile et al. Insulin related lipodystrophic lesions and hypoglycemia: Double standards? Diabetes Metab Syndr. 2018;12(5):813-818<br>182. Gentile et al. Insulin-induced skin lipohypertrophies: A neglected cause of hypoglycemia in dialysed individuals with diabetes. Diabetes Metab Syndr. 2021;15(4):102145<br>183. Gold et al. Analysis: the impact of needle, syringe, and lancet disposal on the community. J Diabetes Sci Technol. 2011;5(4):848-50<br>184. Granda et al. Lipoatrophy, lipodystrophy, and insulin resistance. Ann Intern Med. 2000;133(4):304-6<br>185. Gupta et al. Managing lipohypertrophy in your practice. Journal of the Indian Medical Association. 2018;116:41-43<br>186. Hanas et al. Reducing injection pain in children and adolescents with diabetes: A review of indwelling catheters. Pediatric Diabetes. 2004;5:102-111<br>187. Hirsch et al. The Injection Technique Factor: What You Don't Know or Teach Can Make a Difference. Clin Diabetes. 2019;37(3):227-233<br>188. Hussar et al. Insulin degludec, Lixisenatide, and Patiromer sorbitex calcium. Journal of the American Pharmacists Association. 2016;56:691-694<br>189. Kadiyala et al. Insulin induced lipodystrophy. British Journal of Diabetes and Vascular Disease. 2014;14:131-133<br>190. Kalra et al. Prevention of lipohypertrophy. J Pak Med Assoc. 2016;66(7):910-911<br>191. King et al. Subcutaneous insulin injection technique. Nurs Stand. 2003;17(34):45-52<br>192. Korytkowski et al. FlexPen®: Addressing issues of confidence andconvenience in insulin delivery. Clinical Therapeutics. 2005;27:S89-S100<br>193. Lim et al. Lipodystrophy: a paradigm for understanding the consequences of overloading adipose tissue. Physiological Reviews. 2021;101:907-993<br>194. Madhu et al. The clinical relevance of lipohypertrophy. International Journal of Diabetes in Developing Countries. 2019;39:417-418<br>195. Norman et al. Improving patient acceptance of insulin therapy by improving needle design. J Diabetes Sci Technol. 2012;6(2):336-8<br>196. Pfützner et al. Insulin delivery with FlexPen®: Dose accuracy, patient preference and adherence. Expert Opinion on Drug Delivery. 2008;5:915-925<br>197. Samuel et al. Lipid-induced insulin resistance: unravelling the mechanism. The Lancet. 2010;375:2267-2277<br>198. Slawik et al. Adipose tissue expandability and the metabolic syndrome. Genes and Nutrition. 2007;2:41-45
Study type	Systematic review	199. Foss-Freitas et al. Diagnostic strategies and clinical management of lipodystrophy. Expert Rev Endocrinol Metab. 2020;15(2):95-114<br>200. Kordonouri et al. Lipoatrophy in children with type 1 diabetes: an increasing incidence? J Diabetes Sci Technol. 2015;9(2):206-8

Table 5. Methodology of the included studies

Study	Design (study type)	Centres	Location	Funding	Available endpoints	Dates of data collection	Quality assessment
Abujbara 2022	Cross-sectional	Single-center	Jordan	NA	HbA1c	XI 2020 – II 2021	JBI: 8/8
Al Ajlouni 2015	Cross-sectional	Single-center	Jordan	NA	HbA1c	X 2011 – I 2012	JBI: 6/8
Al Hayek 2016	Cross-sectional	Single-center	Saudi Arabia	Independent study	HbA1c, daily insulin dose, hypoglycemia	VI 2015 – IX 2015	JBI: 5/8
Al Jaber 2020	Cross-sectional	Multi-center	Saudi Arabia	Independent study	HbA1c, daily insulin dose	V 2017 – X 2017	JBI: 6/8
Arora 2021	Cross-sectional	Single-center	India	Independent study	HbA1c, daily insulin dose, hypoglycemia	I 2017 – X 2018	JBI: 6/8
Barola 2018	Cross-sectional	Single-center	India	Independent study	HbA1c, daily insulin dose	NA	JBI: 6/8
Baruah 2017	Cross-sectional	Single-center	India	Independent study	HbA1c, daily insulin dose, hypoglycemia	2006 – 2016	JBI: 7/8
Blanco 2013	Cross-sectional	Multi-center	Spain	Independent study	Daily insulin dose, hypoglycemia, glycemic variability	I 2012 – IX 2012	JBI: 6/8
Bochanen 2021	Prospective, quasi-experimental	Multi-center	Belgium	Independent study,, Becton Dickinson	HbA1c, daily insulin dose, hypoglycemia, glycemic variability	VI 2018 – XI 2018	JBI: 6/9
Cunningham 2013	Cross-sectional	Multi-center	Ireland	NA	Daily insulin dose	NA	JBI: 4/8
Frid 2016	Cross-sectional	Multi-center	World	Becton Dickinson	HbA1c, daily insulin dose, hypoglycemia, glycemic variability	II 2014 – VI 2015	JBI: 5/8
Gentile 2019	Cross-sectional	Multi-center	Italy	Nefrocenter Research Network	HbA1c, daily insulin dose, hypoglycemia, glycemic variability	NA	JBI: 6/8
Gentile 2020	Cross-sectional	Multi-center	Italy	Nefrocenter Research Network, NYX Startup	HbA1c, daily insulin dose, hypoglycemia, glycemic variability	NA	JBI: 6/8
Gentile 2021	Cross-sectional	Multi-center	Italy	Nefrocenter Research Network and NYX StartNAup	HbA1c, daily insulin dose, hypoglycemia, glycemic variability	NA	JBI: 8/8
Gentile 2022	Prospective, quasi-experimental	Multi-center	Italy	Independent study	HbA1c, daily insulin dose, hypoglycemia, FPG	NA	JBI: 5/9
Gunhan 2022	Cross-sectional	Single-center	Turkey	NA	HbA1c, daily insulin dose, FPG	V 2018 – VI 2019	JBI: 6/8
Gupta 2018	Cross-sectional	Single-center	India	NA	HbA1c, daily insulin dose, hypoglycemia, glycemic variability, CGM data	XI 2015 – III 2017	JBI: 6/8
Hajheydari 2011	Cross-sectional	Single-center	Iran	Mazandaran University of Medical Sciences	HbA1c	2007 – 2008	JBI: 4/8
Hauner 1996	Cross-sectional	Single-center	Germany	NA	HbA1c, daily insulin dose	XI 1991 – IV 1992	JBI: 5/8
Ji 2014	Cross-sectional	Multi-center	China	Becton Dickinson	HbA1c	X 2010 – XI 2010	JBI: 5/8
Ji 2017	Cross-sectional	Multi-center	China	Becton Dickinson	HbA1c, daily insulin dose, hypoglycemia	NA	JBI: 6/8
Kamrul-Hasan 2020	Cross-sectional	Multi-center	Bangladesh	Independent study	Daily insulin dose, hypoglycemia, hyperglycemia	I 2018 – XII 2018	JBI: 6/8
Korkmaz 2021	Cross-sectional	Single-center	Turkey	Independent study	HbA1c, daily insulin dose	NA	JBI: 6/8
Kumar 2021	Cross-sectional	Single-center	India	NA	HbA1c, daily insulin dose	III 2016 – II 2017	JBI: 6/8
Lin 2022	Cross-sectional	Single-center	China	Guide Project of FuJian	Daily insulin dose, CGM data	IV 2019 – XII 2019	JBI: 5/8

Study	Design (study type)	Centres	Location	Funding	Available endpoints	Dates of data collection	Quality assessment
				Province of China			
Luo 2021	Cross-sectional	Single-center	China	Jiangsu Provincial Medical Innovation Team (CXTDA2017019), the Drug Evaluation Wei Tai Young Scholars Diabetes Technology Research Fund (YPPJNA02NA17), and the National Natural Science Foundation of China	HbA1c, daily insulin dose	I 2018 – XII 2019	JBI: 8/8
Nawaz 2023	Cross-sectional	Single-center	Pakistan	NA	Hypoglycemia, hyperglycemia	VII 2020 – XII 2020	JBI: 4/8
Omar 2011	Cross-sectional	Single-center	Egypt	NA	HbA1c, daily insulin dose	NA	JBI: 4/8
Pahuja 2019	Cross-sectional	Single-center	India	NA	HbA1c, daily insulin dose	NA	JBI: 5/8
Pozzuoli 2018	Cross-sectional	Single-center	Italy	NA	HbA1c, daily insulin dose, hypoglycemia	NA	JBI: 6/8
Saeed 2022	Cross-sectional	Single-center	Pakistan	NA	Daily insulin dose	I 2022 – VI 2022	JBI: 6/8
Saez de Ibarra 1998	Cross-sectional	Single-center	Spain	NA	Glycemic variability	NA	JBI: 4/8
Singha 2021	Cross-sectional	Single-center	India	Independent study	HbA1c, daily insulin dose, hypoglycemia	VIII 2018 – VII 2019	JBI: 6/8
Strollo 2016	Cross-sectional	Multi-center	Italy	NA	HbA1c, daily insulin dose, hypoglycemia	NA	JBI: 6/8
Sürücü 2018	Cross-sectional	Single-center	Turkey	Independent study	HbA1c, hypoglycemia	VI 2016 – IV 2017	JBI: 5/8
Thewjitcharoen 2020	Cross-sectional	Single-center	Thailand	Theptarin Hospital	HbA1c, daily insulin dose, hypoglycemia	VI 2018 – III 2019	JBI: 6/8
Tsadik 2018	Cross-sectional	Single-center	Ethiopia	Addis Ababa University	Daily insulin dose, hypoglycemia	IV 2017 – VII 2017	JBI: 8/8

Table 6. Baseline characteristics of patients in the included studies

Study	Group	N	Men	Age, years*	Children	Adults	T1DM	T2DM	Duration of diabetes*, years	BMI*, m/kg^2	Overweight	Obesity	Educational status	Socio-economic status
Abujbara 2022	LH+	477	NA	NA	NA	NA	36%	64%	NA	NA	NA	NA	NA	NA
	LH-	374	NA	NA	NA	NA	34%	66%	NA	NA	NA	NA	NA	NA
	Total	851	48.5%	45.1 (20.8)	NA	NA	35%	65%	12.3 (8.1)	30.4 (7.1)	NA	NA	≤High school: 61%<br>University: 39%	NA
Al Ajlouni 2015	LH+	407	NA	NA	0%	100%	0%	100%	NA	NA	25%	69%	Illiterate: 17%<br>≤High school: 46%<br>>High school: 37%	NA
	LH-	683	NA	NA	0%	100%	0%	100%	NA	NA	25%	68%	Illiterate: 10%<br>≤High school: 42.5%<br>>High school:47.5%	NA
	Total	1090	47%	57.1 (10.3)	0%	100%	0%	100%	13.5 (9–20)†	NA	24%	68%	Illiterate: 12%<br>≤High school: 44%<br>>High school:44%	NA
Al Jaber 2020	LH+	80	37.5%	57.6 (10.6)	0%	100%	0%	100%	16.7 (8.7)	34.4 (7.1)	19%	74%	No school: 27.5%<br>Primary school: 20%<br>Secondary school: 19%<br>Tertiary school: 19%<br>University or collage: 15%	Monthly income <5.000: 39%<br>5–10.000 UC: 27.5%<br>10–15.000 UC: 21%<br>>15.000 UC: 12.5%
	LH-	122	48%	59.2 (11.7)	0%	100%	0%	100%	17.1 (8.5)	32.3 (7.0)	27%	61.5%	No school: 29%<br>Primary school: 23%<br>Secondary school: 7%<br>Tertiary school: 19%<br>University or collage: 22%	Monthly income <5.000: 39%<br>5–10.000: 25%<br>10–15.000: 23%<br>>15.000: 13%
	Total	202	44%	58.5 (11.3)	0%	100%	0%	100%	16.9 (8.5)	33.1 (7.1)	24%	66%	No school: 28%<br>Primary school: 22%<br>Secondary school: 12%<br>Tertiary school: 19%<br>University or collage: 19%	Monthly income <5.000: 39%<br>5–10.000: 26%<br>10–15.000: 22%<br>>15.000: 13%
Al Hayek 2016	LH+	83	52%	NA	100%	0%	100%	0%	NA	NA	49%		Primary: 13%<br>Secondary: 87%	NA
	LH-	91	52%	NA	100%	0%	100%	0%	NA	NA	13%		Primary: 51%<br>Secondary: 49%	NA
	Total	174	52%	15.4 (2.0)	100%	0%	100%	0%	6.1 (4.5)	NA	30.5%		Primary: 33%<br>Secondary: 67%	NA
Arora 2021	LH+	290	52%	NA	NA	NA	20%	74%	NA	NA	46%	NA	NA	NA
	LH-	210	58%	NA	NA	NA	26%	67%	NA	NA	42%	NA	NA	NA
	Total	500	54%	45.2 (16.5)	NA	NA	23%	71%	NA	NA	44%	NA	NA	NA
Barola 2018	LH+	231	60%	17.1 (7.8)	66%	34%	100%	0%	5.7 (5.5)	NA	10%	1%	NA	Upper: 6%<br>Upper middle: 38%<br>Lower middle: 29%<br>Upper lower: 27%
	LH-	141	46%	17.2 (6.7)	60%	40%	100%	0%	5.4 (5.1)	NA	13%	9%	NA	Upper: 8%<br>Upper middle: 47.5%<br>Lower middle: 25.5%<br>Upper lower: 19%
	Total	372	55%	17.1 (7.4)	64%	36%	100%	0%	5.6 (5.3)	NA	11%	4%	NA	Upper: 6.5%<br>Upper middle: 41%

Study	Group	N	Men	Age, years*	Children	Adults	T1DM	T2DM	Duration of diabetes*, years	BMI*, m/kg^2	Overweight	Obesity	Educational status	Socio-economic status
														Lower middle: 28%<br>Upper lower: 24%
Baruah 2017	LH+	94	NA	NA	NA	NA	NA	NA	NA	NA	NA	NA	NA	NA
	LH-	654	NA	NA	NA	NA	NA	NA	NA	NA	NA	NA	NA	NA
	Total	748	62%	54.6 (11.0)	2%	98%	0%	100%	12.2 (7.6)	25.1 (4.2)	68%		Illiterate: 2%<br>Undergraduate: 49%<br>Graduate: 49%	NA
Blanco 2013	LH+	277	51%	47.0 (23.8)	NA	NA	NA	NA	NA(6–15)‡	NA	NA	NA	NA	NA
	LH-	153	52%	54.0 (20.1)	NA	NA	NA	NA	NA(6–15)‡	NA	NA	NA	NA	NA
	Total	430	51%	49.0 (22.8)	11%	89%	41%	59%	NA(6–15)‡	NA	NA	NA	NA	NA
Bochanen 2021	LH+	92	60%	57.3 (14.8)	0%	100%	57%	43%	NA	NA	NA	NA	NA	NA
	LH-	54	52%	56.8 (14.7)	0%	100%	43%	57%	NA	NA	NA	NA	NA	NA
	Total	146	57%	57.1 (14.7)	0%	100%	51%	49%	NA	NA	NA	NA	NA	NA
Cunningham 2013	LH+	28	54%	59.5 (15.9)	NA	NA	NA	NA	NA	NA	NA	NA	NA	NA
	LH-	27	33%	50.8 (15.5)	NA	NA	NA	NA	NA	NA	NA	NA	NA	NA
	Total	55	44%	55.2 (16.6)	NA	NA	75%	25%	NA	NA	NA	NA	NA	NA
Frid 2016	LH+	3 855	NA	NA	NA	NA	NA	NA	NA	NA	NA	NA	NA	NA
	LH-	9 334	NA	NA	NA	NA	NA	NA	NA	NA	NA	NA	NA	NA
	Total	13 289	49%	51.9 (18.1)	9%	91%	34%	65%	13.2 (9.7)	26.6 (6.2)	NA	NA	NA	NA
Gentile 2019	LH+	169	NA	63.0 (5.0)	0%	100%	6%	94%	6.0 (5.0)	NA	NA	NA	NA	NA
	LH-	127	NA	64.0 (5.0)	0%	100%	6%	94%	7.0 (3.0)	NA	NA	NA	NA	NA
	Total	296	47%	64.0 (7.0)	0%	100%	6%	94%	7.0 (2.0)	NA	16%	0%	NA	NA
Gentile 2020	LH+	718	42%	61.0 (16.0)	0%	100%	0%	100%	11.0 (7.0)	29.0 (6.0)	NA	NA	Primary school: 42%<br>Middle school: 33%<br>High school: 18%<br>University: 7%	Employed: 21%<br>Unemployed: 19%<br>Retired: 60%
	LH-	509	44%	63.0 (12.0)	0%	100%	0%	100%	10.0 (9.0)	29.0 (6.0)	NA	NA	Primary school: 42%<br>Middle school: 33%<br>High school: 17%<br>University: 7%	Employed: 38%<br>Unemployed: 19%<br>Retired: 42%
	Total	1227	42%	61.8 (14.5)	0%	100%	0%	100%	10.6 (7.9)	29.0 (6.0)	NA	NA	Primary school: 42%<br>Middle school: 33%<br>High school: 18%<br>University: 7%	Employed: 28%<br>Unemployed: 19%<br>Retired: 53%
Gentile 2021	LH+	360	51%	61.0 (15.0)	0%	100%	33%	67%	20.0 (11.0)	29.0 (6.0)	NA	NA	NA	NA
	LH-	420	49%	63.0 (15.0)	0%	100%	25%	75%	17.0 (10.0)	29.0 (6.0)	NA	NA	NA	NA
	Total	780	50%	62.0 (15.0)	0%	100%	29%	71%	18.0 (11.0)	29.0 (6.0)	NA	NA	NA	NA
Gentile 2022	LH+	487	58%	61.9 (5.6)	0%	100%	0%	100%	14.5 (5.2)	31.4 (7.2)	NA	NA	NA	NA
	LH-	673	41%	60.1 (3.6)	0%	100%	0%	100%	15.6 (7.5)	32.7 (5.6)	NA	NA	NA	NA
	Total	1160	48%	60.6 (9.0)	0%	100%	0%	100%	15.8 (7.6)	32.4 (3.5)	NA	NA	NA	NA
Gunhan 2022	LH+	98	61%	57.0 (9.6)	0%	100%	0%	100%	16.0 (8.3)	37.0 (9.1)	NA	NA	NA	NA
	LH-	247	60%	57.0 (11.0)	0%	100%	0%	100%	14.0 (7.3)	37.0 (8.3)	NA	NA	NA	NA
	Total	345	60%	57.0 (10.6)	0%	100%	0%	100%	14.6 (7.6)	37.0 (8.5)	NA	NA	NA	NA
Gupta 2018	LH+	97	51.5%	23.0 (12.4)	38%	62%	100%	0%	9.1 (7.5)	20.1 (4.5)	NA	NA	NA	NA
	LH-	42	59.5%	18.7 (9.7)	57%	43%	100%	0%	7.9 (7.6)	19.4 (5.1)	NA	NA	NA	NA
	Total	139	54%	21.7 (11.8)	44%	56%	100%	0%	8.7 (7.5)	19.9 (4.7)	NA	NA	NA	NA
Hajheydari 2011	LH+	35	49%	34.6 (21.2)	NA	NA	63%	37%	12.7 (9.2)	25.0 (6.7)	29%	14%	Illiterate or elementary: 46%	NA

Study	Group	N	Men	Age, years*	Children	Adults	T1DM	T2DM	Duration of diabetes*, years	BMI*, m/kg^2	Overweight	Obesity	Educational status	Socio-economic status
													Guidance school: 23%<br>High school: 20%<br>University: 11%	
	LH-	185	23%	49.3 (16.3)	NA	NA	18%	82%	14.2 (8.4)	27.4 (5.3)	36%	29%	Illiterate or elementary: 71%<br>Guidance school: 6%<br>High school: 16%<br>University: 7%	NA
	Total	220	27%	49.0 (17.9)	NA	NA	25%	75%	14.0 (8.5)	27.0 (5.6)	35%	26%	Illiterate or elementary: 67%<br>Guidance school: 9%<br>High school: 17%<br>University: 8%	NA
Hauner 1996	LH+	66	NA	NA	NA	NA	97%	3%	NA	NA	NA	NA	NA	NA
	LH-	213	NA	NA	NA	NA	75%	25%	NA	NA	NA	NA	NA	NA
	Total	279	44%	40.2 (18.1)	NA	NA	80%	20%	14.1 (9.5)	24.3 (4.0)	NA	NA	NA	NA
Ji 2014	LH+	134	NA	NA	NA	NA	NA	NA	NA	NA	NA	NA	NA	NA
	LH-	246	NA	NA	NA	NA	NA	NA	NA	NA	NA	NA	NA	NA
	Total	380	50%	54.6 (8.7)	0%	100%	0%	100%	NA	23.2 (2.3)	17%	6%	NA	NA
Ji 2017	LH+	213	52%	59.8 (11.2)	0%	100%	9%	91%	12.4 (7.7)	26.0 (3.3)	49%	24%	NA	NA
	LH-	188	47%	59.3 (11.9)	0%	100%	4%	96%	11.3 (6.8)	24.8 (3.0)	53%	11%	NA	NA
	Total	401	50%	59.6 (11.5)	0%	100%	7%	93%	11.8 (7.3)	25.4 (3.2)	51%	18%	Primary school or below: 12%<br>Junior school: 31%<br>High school: 25%<br>Bachelor's degree: 29%<br>Master's degree: 1%<br>Other: <1%	No income: 8%<br><1000 RMB: 5%<br>1001NA3000 RMB: 50%<br>3001NA5001 RMB: 26%<br>5001NA10000 RMB: 8%<br>10001NA25000 RMB: 1%<br>>25000 RMB: 1%
Kamrul-Hasan 2020	LH+	78	NA	NA	NA	NA	NA	NA	NA	NA	NA	NA	NA	NA
	LH-	769	NA	NA	NA	NA	NA	NA	NA	NA	NA	NA	NA	NA
	Total	847	44%	49.9 (13.1)	NA	NA	2%	94%	9.8 (7.0)	NA	NA	NA	NA	NA
Korkmaz 2021	LH+	119	NA	57.0 (18.0)†	0%	100%	27%	73%	15.0 (13.0)†	30.6 (7.3)	NA	NA	NA	NA
	LH-	17	NA	55.5 (17.0)†	0%	100%	23.5%	76.5%	15.0 (11.0)†	30.5 (8.3)	NA	NA	NA	NA
	Total	136	40%	52.9 (14.9)	0%	100%	26.5%	73.5%	15.8 (9.2)	30.6 (7.4)	NA	NA	NA	NA
Kumar 2021¶	LH+	60 [79]	80%	NA	0%	100%	12%	88%	NA	NA	NA	65%	NA	NA
	LH-	28 [9]	57%	NA	0%	100%	29%	71%	NA	NA	NA	46%	NA	NA
	Total	88	73%	51.3 (15.4)	0%	100%	17%	83%	21.8 (9.9)	26.4 (5.1)	NA	59%	NA	NA
Lin 2022	LH+	83	58%	NA	0%	100%	0%	100%	NA	NA	42%	16%	NA	NA
	LH-	37	59%	NA	0%	100%	0%	100%	NA	NA	32%	13.5%	NA	NA
	Total	120	58%	59.2 (11.4)	0%	100%	0%	100%	NA	24.9 (3.2)	39%	15%	NA	NA
Luo 2021	LH+	270	56%	55.0 (16.1)	0%	100%	25%	75%	NA	24.5 (3.3)	58.5%	NA	Elementary school: 22%<br>Junior school: 32%<br>High school: 20%<br>University: 26%	NA
	LH-	46	52%	49.5 (17.9)	0%	100%	37%	63%	8.5 (3.4–15.1)†	22.5 (2.7)	24%	NA	Elementary school: 15%<br>Junior school: 28%<br>High school: 20%<br>University: 37%	NA
	Total	316	55%	55.5 (17.3)	0%	100%	27%	73%	12.8 (6.6–19.2)†	24.2 (3.3)	53.5%	NA	Elementary school: 21%	NA

Study	Group	N	Men	Age, years*	Children	Adults	T1DM	T2DM	Duration of diabetes*, years	BMI*, m/kg^2	Overweight	Obesity	Educational status	Socio-economic status
													Junior school: 32%<br>High school: 20%<br>University: 27%	
Nawaz 2023	LH+	83	29%	NA	NA	NA	NA	NA	NA	NA	NA	NA	NA	NA
	LH-	280	46%	NA	NA	NA	NA	NA	NA	NA	NA	NA	NA	NA
	Total	363	42%	49.7 (13.4)	1%	99%	NA	NA	7.9 (4.5)	NA	NA	NA	NA	NA
Omar 2011	LH+	62	NA	NA	100%	0%	100%	0%	NA	NA	NA	NA	NA	NA
	LH-	51	NA	NA	100%	0%	100%	0%	4.1 (3.7)	NA	NA	NA	NA	NA
	Total	119	46%	10 (0.7–21)‡	100%	0%	100%	0%	NA	NA	15%	3.5%	NA	NA
Pahuja 2019	LH+	65	65%	NA	0%	100%	0%	100%	21.7 (NA)	NA	NA	NA	No school: 5%<br>Up to 9th grade: 18%<br>10th-12th grade: 29%<br>Graduate/post graduate: 48%	Employed: 21.5%<br>Homemaker: 21.5%<br>Retired/disabled: 57%<br>Income <25000 INR: 28%<br>INR 25000-50000: 43%<br>INR >50 00: 29%
	LH-	31	58%	NA	0%	100%	0%	100%	15.7 (NA)	NA	NA	NA	No school: 10%<br>Up to 9th grade: 10%<br>10thNA12th grade: 35%<br>Graduate/post graduate: 45%	Employed: 19%<br>Homemaker: 35%<br>Retired/disabled: 45%<br>Income <25000 INR: 19%<br>INR 25000-50000: 45%<br>INR >50 00: 35%
	Total	96	62%	65.5 (NA)	0%	100%	0%	100%	19.8 (NA)	NA	NA	NA	No school: 6%<br>Up to 9th grade: 16%<br>10thNA12th grade: 31%<br>Graduate/post graduate: 47%	Employed: 21%<br>Homemaker: 26%<br>Retired/disabled: 53%<br>Income <25000 INR: 24%<br>INR 25000NA50000: 42%<br>INR >50 000: 34%
Pozzuoli 2018	LH+	151	44%	68.2 (12.6)	NA	NA	15%	85%	21.9 (10.2)	NA	NA	NA	NA	NA
	LH-	201	42%	67.3 (11.9)	NA	NA	8%	92%	19.2 (9.5)	NA	NA	NA	NA	NA
	Total	352	43%	67.7 (12.2)	NA	NA	11%	89%	20.4 (9.9)	NA	NA	NA	NA	NA
Saeed 2022	LH+	157	45%	NA	NA	NA	14%	86%	NA	NA	NA	NA	None: 54%<br>Primary: 21%<br>Middle: 15%<br>High school: 1%<br>University: 9%	NA
	LH-	203	39%	NA	NA	NA	8%	92%	NA	NA	NA	NA	None: 55%<br>Primary: 16%<br>Middle: 12%<br>High school: 13%<br>University: 4%	NA
	Total	360	41%	50.8 (11.0)	NA	NA	11%	89%	14.7 (7.6)	NA	NA	NA	None: 54%<br>Primary: 18%<br>Middle: 13%<br>High school: 8%<br>University: 6%	NA
Saez de Ibarra 1998	LH+	78	NA	NA	NA	NA	NA	NA	NA	NA	NA	NA	NA	NA
	LH-	72	NA	NA	NA	NA	NA	NA	NA	NA	NA	NA	NA	NA

Study	Group	N	Men	Age, years*	Children	Adults	T1DM	T2DM	Duration of diabetes*, years	BMI*, m/kg^2	Overweight	Obesity	Educational status	Socio-economic status
	Total	150	38%	36.9 (17.9)	NA	NA	75%	25%	13.3 (8.8)	24.5 (3.5)	NA	NA	NA	NA
Singha 2021	LH+	46	43%	NA	NA	NA	100%	0%	NA	NA	NA	NA	NA	NA
	LH-	45	31%	NA	NA	NA	100%	0%	NA	NA	NA	NA	NA	NA
	Total	95	36%	13.3 (4.1)	NA	NA	100%	0%	NA	NA	NA	NA	NA	NA
Strollo 2016	LH+	298	34%	61.0 (10.0)	0%	100%	20%	80%	20.0 (8.0)	29.0 (6.0)	NA	NA	NA	NA
	LH-	89	98%	63.0 (12.0)	0%	100%	22%	78%	17.0 (9.0)	29.0 (6.0)	NA	NA	NA	NA
	Total	387	45.5%	61.0 (16.0)	0%	100%	21%	79%	13.0 (9.0)	29.0 (8.0)	NA	NA	NA	NA
Sürücü 2018	LH+	191	37%	NA	0%	100%	0%	100%	NA	NA	44.5%	42%	Non-literate: 8%<br>Primary school: 36%<br>Secondary school: 21%<br>High school or university graduate: 35%	NA
	LH-	245	36%	NA	0%	100%	0%	100%	NA	NA	51%	29%	Non-literate: 7%<br>Primary school: 33%<br>Secondary school: 32%<br>High school or university graduate: 28%	NA
	Total	436	36.5%	NA	0%	100%	0%	100%	NA	NA	48%	35%	Non-literate: 8%<br>Primary school: 34%<br>Secondary school: 27%<br>High school or university graduate: 31%	NA
Thewjitcharoen 2020	LH+	149	52%	64.8 (14.2)	NA	NA	17%	83%	24.1 (8.9)	25.9 (4.8)	NA	NA	Less than high school: 37%<br>High school: 21%<br>Bachelor degree or college: 34%<br>Higher than bachelor degree: 8%	NA
	LH-	251	43%	66.0 (16.1)	NA	NA	12%	88%	22.4 (10.8)	26.3 (4.8)	NA	NA	Less than high school: 40%<br>High school: 22%<br>Bachelor degree or college: 26%<br>Higher than bachelor degree: 12%	NA
	Total	400	46.5%	65.6 (15.4)	NA	NA	14%	86%	23.0 (10.2)	26.2 (4.8)	NA	NA	Less than high school: 39%<br>High school: 21.5%<br>Bachelor degree or college: 29%<br>Higher than bachelor degree: 10.5%	NA
Tsadik 2018	LH+	103	51%	NA	100%	0%	100%	0%	NA	NA	0%	6%	No schooling: 1%<br>Primary: 51%<br>Secondary: 32%<br>Higher: 16%	NA
	LH-	73	45%	NA	100%	0%	100%	0%	NA	NA	0%	6%	No schooling: 1%<br>Primary: 44%<br>Secondary: 33%<br>Higher: 22%	NA
	Total	176	49%	11.4 (4.0)	100%	0%	100%	0%	NA	NA	0%	6%	No schooling: 1%<br>Primary: 48%<br>Secondary: 32%<br>Higher: 18%	NA

*Data are given as mean (SD) unless otherwise stated. †Median (IQR). ‡Median (range). §Data in square brackets refers to multiple daily injections patients. ¶Data in square brackets includes patients with subclinical lipohypetropy. NA – not available.

Table 7. Characteristics of the insulin therapy in the included studies

Study	Group	N	Duration of insulin therapy*, years	MDI	CSII	Syringes	Pens	Basal insulin	Bolus insulin	Human insulin	Analogs	Pre-mix	Daily insulin injections*	Reuse	Proper rotation	Needle size
Abujbara 2022	LH+	477	NA	NA	NA	NA	NA	NA	NA	NA	NA	NA	NA	NA	NA	NA
	LH-	374	NA	NA	NA	NA	NA	NA	NA	NA	NA	NA	NA	NA	NA	NA
	Total	851	7.0 (6.2)	100%	0%	NA	NA	NA	NA	NA	NA	NA	NA	94%	43%	4 mm: 8%<br>5 mm: 11%<br>6 mm:18%<br>8 mm: 63%
Al Ajlouni 2015	LH+	407	NA	100%	0%	NA	NA	NA	NA	NA	NA	NA	NA	NA	14%	8 mm: 82%<br>12 mm: 18%
	LH-	683	NA	100%	0%	NA	NA	NA	NA	NA	NA	NA	NA	NA	91%	8 mm: 95%<br>12 mm: 5%
	Total	1090	4.6 (5.0)	100%	0%	NA	NA	NA	NA	NA	NA	58%	NA	NA	62%	8 mm: 90%<br>12 mm: 10%
Al Hayek 2016	LH+	83	NA	100%	0%	NA	NA	100%	100%	NA	NA	NA	NA	69%	28%	4 mm: 18%<br>6 mm: 2%<br>8 mm: 80%
	LH-	91	NA	100%	0%	NA	NA	100%	100%	NA	NA	NA	NA	25%	85%	4 mm: 53%<br>6 mm: 40%<br>8 mm: 8%
	Total	174	NA	100%	0%	NA	NA	100%	100%	NA	NA	NA	NA	46%	57.5%	4 mm: 36%<br>6 mm: 22%<br>8 mm: 42%
Al Jaber 2020	LH+	80	8.8 (6.1)	100%	0%	34%	66%	42.5%	35%	NA	NA	57.5%	2.6 (1.0)	43%	69%	4 mm: 62.5%<br>5 mm: 6%<br>6 mm: 4%<br>8 mm: 4%<br>Don't know: 24%
	LH-	122	8.4 (5.7)	100%	0%	23%	77%	56%	40 .5%	NA	NA	44%	2.4 (1.2)	37.5%	60%	4 mm: 61%<br>5 mm: 9%<br>6 mm: 2%<br>8 mm: 2%<br>Don't know: 27%
	Total	202	8.5 (5.8)	100%	0%	27%	73%	50%	38%	NA	NA	49%	2.5 (1.1)	39%	64%	4 mm: 61%<br>5 mm: 8%<br>6 mm: 2.5%<br>8 mm: 2.5%<br>Don't know: 26%
Arora 2021	LH+	290	NA	100%	0%	58%	42%	NA	NA	80%	20%	NA	NA	42%	37%	NA
	LH-	210	NA	100%	0%	55%	45%	NA	NA	70%	30%	NA	NA	20%	72%	NA
	Total	500	3.0 (2.5–5.0)†	100%	0%	57%	43%	NA	NA	76%	24%	NA	NA	33%	52%	NA
Barola 2018	LH+	231	NA	100%	0%	33%	75%	82%	82%	55%	73%	18%	NA	73%	16%	4 mm: 56%<br>5 mm: 3%<br>6 mm: 33%<br>8 mm: 7%
	LH-	141	NA	100%	0%	16%	88%	96%	96%	31%	91%	4%	NA	69%	74%	4 mm: 65%<br>5 mm: 8%<br>6 mm: 23%<br>8 mm: 4%

Study	Group	N	Duration of insulin therapy*, years	MDI	CSII	Syringes	Pens	Basal insulin	Bolus insulin	Human insulin	Analogs	Pre-mix	Daily insulin injections*	Reuse	Proper rotation	Needle size
	Total	372	NA	100%	0%	26%	80%	87%	87%	40%	80%	13%	NA	72%	38%	4 mm: 59%<br>5 mm: 5%<br>6 mm: 30%<br>8 mm: 6%
Baruah 2017	LH+	94	3.7 (4.5)	NA	NA	NA	NA	NA	NA	NA	NA	NA	2.1 (0.7)	NA	NA	NA
	LH-	654	3.4 (4.1)	NA	NA	NA	NA	NA	NA	NA	NA	NA	2.1 (0.7)	NA	NA	NA
	Total	748	3.4 (4.2)	100%	0%	34%	68%	21%	51%	64%	41%	78%	2.1 (0.7)	72%	30%	NA
Blanco 2013	LH+	277	NA (6–13)‡	100%	0%	5%	95%	75%	60%	23%	77%	15%	3.4 (1.4)	61%	52%<br>(2%)§	8 mm: 69%
	LH-	153	NA (1–5)‡	100%	0%	1%	99%	78%	39%	20%	80%	12%	2.4 (1.5)	47%	93%<br>(65%)§	8 mm: 67%
	Total	430	NA (1–5)‡	100%	0%	4%	96%	76%	52%	22%	78%	14%	3.0 (1.5)	56%	67%<br>(25%)§	5 mm: 21%<br>6 mm: 4%<br>8 mm: 68%<br>12.7 mm: 7%
Bochanen 2021	LH+	92	NA	100%	0%	0%	100%	NA	NA	NA	NA	NA	NA	95%	53%	4 mm: 49%<br>5 mm: 23%<br>6 mm: 21%<br>8 mm: 8%
	LH-	54	NA	100%	0%	0%	100%	NA	NA	NA	NA	NA	NA	98%	80%	4 mm: 56%<br>5 mm: 24%<br>6 mm: 15%<br>8 mm: 6%
	Total	146	NA	100%	0%	0%	100%	NA	NA	NA	NA	NA	NA	96%	63%	4 mm: 51%<br>5 mm: 23%<br>6 mm: 18.5%<br>8 mm: 7%
Cunningham 2013	LH+	28	19.2 (13.6)	100%	0%	0%	100%	NA	NA	NA	NA	NA	NA	88%	23%	≤6 mm: 36%<br>>6 mm: 64%
	LH-	27	10.6 (10.0)	100%	0%	0%	100%	NA	NA	NA	NA	NA	NA	81%	85%	≤6 mm: 56%<br>>6 mm: 44%
	Total	55	15.0 (12.6)	100%	0%	0%	100%	NA	NA	NA	NA	NA	NA	15%	53%	≤6 mm: 45%<br>>6 mm: 55%
Frid 2016	LH+	3 855	NA	NA	NA	NA	NA	NA	NA	NA	NA	NA	NA	NA	NA	NA
	LH-	9 334	NA	NA	NA	NA	NA	NA	NA	NA	NA	NA	NA	NA	NA	NA
	Total	13 289	8.7 (8.9)	99%	1%	13%	89%	NA	NA	19%	62%	14%	3.0 (1.3)	53%	90%<br>(71%)§	4 mm: 21%<br>5 mm: 29%<br>6 mm: 15%<br>8 mm: 16%<br>12.7 mm: 1%
Gentile 2019	LH+	169	2.7 (1.3)	100%	0%	NA	NA	NA	NA	NA	NA	NA	NA	NA	78%	4 mm: 10%<br>5 mm: 15%<br>6 mm: 40%<br>8 mm: 35%
	LH-	127	3.1 (2.1)	100%	0%	NA	NA	NA	NA	NA	NA	NA	NA	NA	12%	4 mm: 32%<br>5 mm: 31%<br>6 mm: 30%<br>8 mm: 7%

Study	Group	N	Duration of insulin therapy*, years	MDI	CSII	Syringes	Pens	Basal insulin	Bolus insulin	Human insulin	Analogs	Pre-mix	Daily insulin injections*	Reuse	Proper rotation	Needle size
	Total	296	3.0 (1.0)	100%	0%	20%	80%	NA	NA	NA	NA	NA	3.7 (0.5)	68%	26%	4 mm: 11%<br>5 mm: 35%<br>6 mm: 36%<br>8 mm: 18%
Gentile 2020	LH+	718	8.0 (6.0)	100%	0%	0%	100%	100%	100%	0%	100%	0%	NA	98%	2%	4 mm: 13%<br>5 mm: 30%<br>6 mm: 38%<br>8 mm: 19%
	LH-	509	7.0 (6.0)	100%	0%	0%	100%	100%	100%	0%	100%	0%	NA	19%	70%	4 mm: 12.5%<br>5 mm: 30%<br>6 mm: 38%<br>8 mm: 17%
	Total	1227	7.6 (6.0)	100%	0%	0%	100%	100%	100%	0%	100%	0%	NA (3–4)‡	65%	24%	4 mm: 13%<br>5 mm: 30%<br>6 mm: 38%<br>8 mm: 18%
Gentile 2021	LH+	360	12.2 (2.1)	100%	0%	0%	100%	NA	NA	NA	NA	7%	3.7 (0.9)	32.5%	40%	4 mm: 27.5%<br>5 mm: 22%<br>6 mm: 24%<br>8 mm: 27%<br>12.7 mm: 1%
	LH-	420	8.8 (2.9)	100%	0%	0%	100%	NA	NA	NA	NA	8%	3.7 (3.5)	14.5%	87%	4 mm: 29%<br>5 mm: 38%<br>6 mm: 17%<br>8 mm: 15.5%<br>12.7 mm: 0%
	Total	780	10.1 (2.1)	100%	0%	0%	100%	NA	NA	NA	NA	7%	3.7 (2.6)	23%	65%	4 mm: 28.5%<br>5 mm: 31%<br>6 mm: 20%<br>8 mm: 21%<br>12.7 mm: <1%
Gentile 2022	LH+	487	7.2 (3.1)	100%	0%	0%	100%	NA	NA	NA	NA	NA	3.8 (0.5)	98.5%	6%	NA
	LH-	673	7.7 (2.5)	100%	0%	0%	100%	NA	NA	NA	NA	NA	3.3 (0.7)	9%	98%	NA
	Total	1160	7.6 (2.2)	100%	0%	0%	100%	NA	NA	NA	NA	NA	3.5 (0.7)	46%	57%	NA
Gunhan 2022	LH+	98	10.0 (6.2)	100%	0%	NA	NA	79%	71%	NA	NA	20%	3.7 (1.0)	NA	NA	4 mm: 54%<br>5 mm: 18%<br>6 mm: 20%<br>8 mm: 7%
	LH-	247	8.4 (5.5)	100%	0%	NA	NA	68%	56%	NA	NA	32%	3.2 (1.3)	NA	NA	4 mm: 61%<br>5 mm: 11%<br>6 mm: 17%<br>8 mm: 10%
	Total	345	8.9 (5.7)	100%	0%	NA	NA	71%	60%	NA	NA	29%	3.3 (1.2)	NA	NA	4 mm: 59%<br>5 mm: 13%<br>6 mm: 19%<br>8 mm: 9%
Gupta 2018	LH+	97	NA	100%	0%	60%	40%	NA	NA	78%	34%	17.5%	3.6 (0.8)	64%	2%	NA
	LH-	42	NA	100%	0%	81%	19%	NA	NA	76%	40%	17%	3.5 (0.8)	43%	93%	NA

Study	Group	N	Duration of insulin therapy*, years	MDI	CSII	Syringes	Pens	Basal insulin	Bolus insulin	Human insulin	Analogs	Pre-mix	Daily insulin injections*	Reuse	Proper rotation	Needle size
	Total	139	NA	100%	0%	66%	34%	NA	NA	78%	36%	17%	3.6 (0.8)	58%	29%	NA
Hajheydari 2011	LH+	35	8.4 (6.7)	NA	NA	NA	NA	NA	NA	100%	0%	NA	NA	NA	NA	NA
	LH-	185	4.9 (5.7)	NA	NA	NA	NA	NA	NA	100%	0%	NA	NA	NA	NA	NA
	Total	220	5.4 (6.0)	NA	NA	NA	NA	NA	NA	100%	0%	NA	NA	NA	NA	NA
Hauner 1996	LH+	66	NA	NA	NA	NA	NA	NA	NA	NA	NA	NA	NA	NA	NA	NA
	LH-	213	NA	NA	NA	NA	NA	NA	NA	NA	NA	NA	NA	NA	NA	NA
	Total	279	NA	NA	NA	NA	NA	NA	NA	83%	0%	NA	NA	NA	NA	NA
Ji 2014	LH+	134	NA	NA	NA	NA	NA	NA	NA	NA	NA	NA	NA	NA	NA	NA
	LH-	246	NA	NA	NA	NA	NA	NA	NA	NA	NA	NA	NA	NA	NA	NA
	Total	380	3.6 (4.1)	100%	0%	0%	100%	NA	NA	NA	NA	NA	2.2 (0.9)	91%	92%	5 mm: 75%<br>6 mm: 5%<br>8 mm: 22%<br>Other: 1%
Ji 2017	LH+	213	6.2 (5.0)	100%	0%	0%	100%	NA	NA	NA	NA	55%	2.3 (1.0)	97%	68%	4 mm: 10%<br>5 mm: 57%<br>6 mm: 18%<br>8 mm: 15%
	LH-	188	5.4 (4.0)	100%	0%	0%	100%	NA	NA	NA	NA	52%	1.9 (0.9)	93%	92%	4 mm: 9%<br>5 mm: 63%<br>6 mm: 17%<br>8 mm: 11%
	Total	401	5.8 (4.5)	100%	0%	0%	100%	NA	NA	NA	NA	54%	2.1 (1.0)	95%	79%	4 mm: 10%<br>5 mm: 60%<br>6 mm: 17%<br>8 mm: 13%
Kamrul-Hasan 2020	LH+	78	NA	NA	NA	NA	NA	NA	NA	NA	NA	NA	NA	NA	NA	NA
	LH-	769	NA	NA	NA	NA	NA	NA	NA	NA	NA	NA	NA	NA	NA	NA
	Total	847	3.8 (4.1)	100%	0%	68%	32%	42%	26%	77%	32%	56%	2.4 (NA)	99%	84.5%	4 mm: 8%<br>5 mm: 14%<br>6 mm: 32%<br>8 mm: 40.5%<br>12 mm: 5%
Korkmaz 2021	LH+	119	10.0 9.5)†	100%	0%	NA	NA	54%	54%	NA	NA	37%	4 (2)†	NA	NA	NA
	LH-	17	10.0 (10.3)†	100%	0%	NA	NA	29%	29%	NA	NA	71%	2 (2)†	NA	NA	NA
	Total	136	11.4 (8.3)	100%	0%	NA	NA	46%	46%	NA	NA	40%	NA	NA	NA	NA
Kumar 2021	LH+	60 [79]¦	NA	100%	0%	88%	15%	27%	27%	63%	37%	63%	NA	63%	3%	4 mm: 12%<br>6 mm: 78%<br>8 mm: 10%
	LH-	28 [9]¦	NA	100%	0%	82%	25%	18%	18%	61%	39%	57%	NA	41%	32%	4 mm: 18%<br>6 mm: 68%<br>8 mm: 14%
	Total	88	6.5 (6.6)	100%	0%	86%	18%	24%	24%	63%	37%	61%	NA	56%	12.5%	4 mm: 14%<br>6 mm: 75%<br>8 mm: 11%
Lin 2022	LH+	83	NA	100%	0%	NA	NA	NA	NA	NA	NA	NA	3.3 (1.1)	69%	48%	4 mm: 36%<br>6 mm: 37%<br>8 mm: 26.5%
	LH-	37	NA	100%	0%	NA	NA	NA	NA	NA	NA	NA	1.7 (1.0)	38%	68%	4 mm: 43%

Study	Group	N	Duration of insulin therapy*, years	MDI	CSII	Syringes	Pens	Basal insulin	Bolus insulin	Human insulin	Analogs	Pre-mix	Daily insulin injections*	Reuse	Proper rotation	Needle size
																6 mm: 43%<br>8 mm: 13.5%
	Total	120	6.6 (4.3)	100%	0%	NA	NA	NA	NA	NA	NA	NA	2.8 (1.3)	57%	55%	4 mm: 38%<br>6 mm: 39%<br>8 mm: 22.5%
Luo 2021	LH+	270	NA	100%	0%	0%	100%	NA	NA	NA	NA	NA	NA	61%	51%	4 mm: 54%<br>5 mm: 35%<br>≥6 mm: 4%
	LH-	46	3.4 (1.1–8.0)†	100%	0%	0%	100%	NA	NA	NA	NA	NA	1.0 (1.0–2.0)†	20%	70%	4 mm: 59%<br>5 mm: 33%<br>≥6 mm: 8%
	Total	316	6.2 (2.9–10.4)†	100%	0%	0%	100%	NA	NA	NA	NA	NA	2.0 (1.0–4.0)†	55%	53.5%	4 mm: 58%<br>5 mm: 37%<br>≥6 mm: 5%
Nawaz 2023	LH+	83	NA	100%	0%	93%	7%	NA	NA	NA	NA	NA	NA	100%	59%	NA
	LH-	280	NA	100%	0%	91%	9%	NA	NA	NA	NA	NA	NA	100%	96%	NA
	Total	363	6.5 (3.7)	100%	0%	91%	9%	NA	NA	NA	NA	NA	2.5 (0.8)	100%	87%	NA
Omar 2011	LH+	51	NA	100%	0%	NA	NA	NA	NA	NA	NA	NA	NA	NA	NA	NA
	LH-	62	NA	100%	0%	NA	NA	NA	NA	NA	NA	NA	NA	NA	NA	NA
	Total	119	NA	100%	0%	87%	13%	NA	NA	NA	6%	NA	NA	NA	NA	8 mm: 69%
Pahuja 2019	LH+	65	8.2 (NA)	100%	0%	28%	72%	35%	40%	NA	NA	51%	NA	20%	82%	NA
	LH-	31	3.7 (NA)	100%	0%	13%	87%	35%	32%	NA	NA	52%	NA	16%	89%	NA
	Total	96	6.8 (NA)	100%	0%	23%	77%	35%	37.5%	NA	NA	51%	2.5 (NA)	19%	84.5%	NA
Pozzuoli 2018	LH+	151	11.6 (9.6)	100%	0%	9%	93%	NA	NA	7%	95%	NA	3.4 (1.0)	11%	46%	4 mm: 16%<br>5 mm: 33%<br>6 mm: 35%<br>8 mm: 15%
	LH-	201	7.2 (7.3)	100%	0%	3.5%	96.5%	NA	NA	2.5%	99%	NA	2.9 (1.3)	7%	60%	4 mm: 26%<br>5 mm: 38%<br>6 mm: 30.5%<br>8 mm: 5%
	Total	352	9.1 (8.6)	100%	0%	6%	95%	NA	NA	4%	98%	NA	3.1 (1.2)	9%	54%	4 mm: 22%<br>5 mm: 36%<br>6 mm: 33%<br>8 mm: 10%
Saeed 2022	LH+	157	NA	100%	0%	87%	13%	18%	18%	73%	27%	82%	NA	100%	91%	4 mm: 5%<br>5 mm: 36%<br>6 mm: 60%
	LH-	203	NA	100%	0%	97%	3%	22%	22%	83%	17%	78%	NA	97%	97%	4 mm: 0%<br>5 mm: 14%<br>6 mm: 86%
	Total	360	8.5 (6.1)	100%	0%	92%	8%	20%	20%	79%	21%	80%	NA	98%	94%	4 mm: 2%<br>5 mm: 23%<br>6 mm: 75%
Saez de Ibarra 1998	LH+	78	NA	NA	NA	NA	NA	NA	NA	NA	NA	NA	NA	NA	NA	NA
	LH-	72	NA	NA	NA	NA	NA	NA	NA	NA	NA	NA	NA	NA	NA	NA
	Total	150	11.4 (7.9)	100%	0%	NA	NA	NA	NA	NA	NA	NA	2.6 (0.5)	NA	79% (29%)§	NA

Study	Group	N	Duration of insulin therapy*, years	MDI	CSII	Syringes	Pens	Basal insulin	Bolus insulin	Human insulin	Analogs	Pre-mix	Daily insulin injections*	Reuse	Proper rotation	Needle size
Singha 2021	LH+	46	5.3 (2.7)	100%	0%	100%	0%	100%	0%	100%	0%	0%	NA	NA	39%	6 mm: 100%
	LH-	45	5.5 (2.8)	100%	0%	100%	0%	100%	0%	100%	0%	0%	NA	NA	64%	6 mm: 100%
	Total	95	NA	100%	0%	100%	0%	100%	0%	100%	0%	0%	NA (3–4)‡	NA	51%	6 mm: 100%
Strollo 2016	LH+	298	12.0 (6.0)	100%	0%	0%	100%	NA	NA	NA	NA	NA	3.7 (9.0)	89%	NA	NA
	LH-	89	7.0 (9.0)	100%	0%	0%	100%	NA	NA	NA	NA	NA	3.7 (3.5)	69%	43%	NA
	Total	387	10.0 (9.0)	100%	0%	0%	100%	91%	93%	3%	NA	9%	3.8 (1.2)	84%	90%	4 mm: 15%<br>5 mm: 16%<br>6 mm: 30%<br>8 mm: 38%<br>12.7 mm: 2%
Sürücü 2018	LH+	191	NA	100%	0%	0%	100%	55%	31%	NA	NA	45%	2.5 (1.2)	70%	42%	4 mm: 33%<br>5 mm: 25%<br>6 mm: 31%<br>8 mm: 12%
	LH-	245	NA	100%	0%	0%	100%	62%	27%	NA	NA	38%	2.2 (1.1)	59%	80%	4 mm: 29%<br>5 mm: 42%<br>6 mm: 24%<br>8 mm: 5%
	Total	436	NA	100%	0%	0%	100%	59%	28%	NA	NA	41%	2.3 (1.2)	64%	63.5%	4 mm: 30.5%<br>5 mm: 35%<br>6 mm: 27%<br>8 mm: 8%
Thewjitcharoen 2020	LH+	149	13.4 (9.1)	100%	0%	9%	93%	57%	18%	41%	64%	41%	NA	97%	40%	4 mm: 3%<br>5 mm: 20%<br>6 mm: 50%<br>8 mm: 25.5%<br>Others: 1%
	LH-	251	10.3 (8.1)	100%	0%	5%	96%	59%	24%	28%	76%	37%	NA	93%	95%	4 mm: 6%<br>5 mm: 21%<br>6 mm: 50%<br>8 mm: 22%<br>Others: 2%
	Total	400	11.4 (8.7)	100%	0%	6%	95%	58%	22%	33%	72%	39%	NA	94.5%	74.5%	4 mm: 5%<br>5 mm: 20.5%<br>6 mm: 50%<br>8 mm: 23%<br>Others: 1%
Tsadik 2018	LH+	103	NA	100%	0%	100%	0%	100%	NA	100%	0%	0%	2.0 (0)	85%	22%	8 mm: 100%
	LH-	73	NA	100%	0%	100%	0%	100%	NA	100%	0%	0%	2.0 (0)	75%	42%	8 mm: 100%
	Total	176	NA	100%	0%	100%	0%	100%	98%	100%	0%	0%	2.0 (0)	83%	31%	8 mm: 100%

*Data are given as mean (SD) unless otherwise stated. †Median (IQR). ‡Median (range). §Claimed (correct). ¶ Including injection ports. |Data in square brackets refers to patients with subclinical lipohypetropy. **Data in square brackets refers to multiple daily injections patients. NA – not available. UC – unknown currency

Table 8. Detailed risk of bias in cross-sectional studies (JBI questionnaire)

Study	Were the criteria for inclusion in the sample clearly defined?	Were the study subjects and the setting described in detail?	Was the exposure measured in a valid and reliable way?	Were objective, standard criteria used for measurement of the condition?	Were confounding factors identified?	Were strategies to deal with confounding factors stated?	Were the outcomes measured in a valid and reliable way?	Was appropriate statistical analysis used?
Abujbara 2022	Yes	Yes	Yes	Yes	Yes	Yes	Yes	Yes
Al Ajlouni 2015	No	No	Yes	Yes	Yes	Yes	Yes	Yes
Al Hayek 2016	Yes	No	Yes	Yes	No	No	Yes	Yes
Al Jaber 2020	Yes	Yes	Yes	Yes	No	No	Yes	Yes
Arora 2021	Yes	Yes	Yes	Yes	No	No	Yes	Yes
Barola 2018	Yes	Yes	Yes	Yes	No	No	Yes	Yes
Baruah 2017	Yes	Yes	Yes	Yes	Yes	No	Yes	Yes
Blanco 2013	Yes	Yes	Yes	Yes	No	No	Yes	Yes
Cunningham 2013	No	No	Yes	Yes	No	No	Yes	Yes
Frid 2016	No	Yes	Yes	Yes	No	No	Yes	Yes
Gentile 2019	Yes	Yes	Yes	Yes	No	No	Yes	Yes
Gentile 2020	Yes	Yes	Yes	Yes	No	No	Yes	Yes
Gentile 2021	Yes	Yes	Yes	Yes	Yes	Yes	Yes	Yes
Gunhan 2022	Yes	Yes	Yes	Yes	No	No	Yes	Yes
Gupta 2018	Yes	Yes	Yes	Yes	No	No	Yes	Yes
Hajheydari 2011	No	No	Yes	Yes	No	No	Yes	Yes
Hauner 1996	Yes	No	Yes	Yes	No	No	Yes	Yes
Ji 2014	Yes	No	Yes	Yes	No	No	Yes	Yes
Ji 2017	Yes	Yes	Yes	Yes	No	No	Yes	Yes
Kamrul-Hasan 2020	Yes	Yes	Yes	Yes	No	No	Yes	Yes
Korkmaz 2021	Yes	Yes	Yes	Yes	No	No	Yes	Yes
Kumar 2021	Yes	Yes	Yes	Yes	No	No	Yes	Yes
Lin 2022	Yes	Yes	Yes	Yes	No	No	No	Yes
Luo 2021	Yes	Yes	Yes	Yes	Yes	Yes	Yes	Yes
Nawaz 2023	Yes	Yes	No	Yes	No	No	No	Yes
Omar 2011	No	No	Yes	Yes	No	No	Yes	Yes
Pahuja 2019	No	Yes	Yes	Yes	No	No	Yes	Yes
Pozzuoli 2018	Yes	Yes	Yes	Yes	No	No	Yes	Yes

Study	Were the criteria for inclusion in the sample clearly defined?	Were the study subjects and the setting described in detail?	Was the exposure measured in a valid and reliable way?	Were objective, standard criteria used for measurement of the condition?	Were confounding factors identified?	Were strategies to deal with confounding factors stated?	Were the outcomes measured in a valid and reliable way?	Was appropriate statistical analysis used?
Saeed 2022	Yes	Yes	Yes	Yes	No	No	Yes	Yes
Saez de Ibarra 1998	No	No	Yes	Yes	No	No	Yes	Yes
Singha 2021	Yes	Yes	Yes	Yes	No	No	Yes	Yes
Strollo 2016	Yes	Yes	Yes	Yes	No	No	Yes	Yes
Sürücü 2018	Yes	No	Yes	Yes	No	No	Yes	Yes
Thewjitcharoen 2020	Yes	Yes	Yes	Yes	No	No	Yes	Yes
Tsadik 2018	Yes	Yes	Yes	Yes	Yes	Yes	Yes	Yes

Table 9. Detailed risk of bias in quasi-experimental studies (JBI questionnaire)

Study	Is it clear in the study what is the 'cause' and what is the 'effect' (i.e. there is no confusion about which variable comes first)	Were the participants included in any comparisons similar?	Were the participants included in any comparisons receiving similar treatment/care, other than the exposure or intervention of interest?	Was there a control group?	Were there multiple measurements of the outcome both pre and post the intervention/exposure?	Was follow up complete and if not, were differences between groups in terms of their follow up adequately described and analyzed?	Were the outcomes of participants included in any comparisons measured in the same way?	Were outcomes measured in a reliable way?	Was appropriate statistical analysis used?
Bochanen 2021	No	Yes	Yes	No	No	Yes	Yes	Yes	Yes
Gentile 2022	No	Yes	Yes	No	No	No	Yes	Yes	Yes

Table 10. Definitions of exposure (lipohypertrophy) in the included studies

Study	Type of measurement	Lipohypertrophy definition
Abujbara 2022	Clinical (visual inspection and palpation)	The researchers examined the site of insulin injection for the presence of LH (grade 1, grade 2, or grade 3). Grade 1 lipohypertrophy was defined as visible hypertrophy of fat tissue but palpably normal, while grade 2 as a massive thickening of fat tissue with firm consistency and grade 3 as lipoatrophy.
Al Ajlouni 2015	Clinical (visual inspection and palpation)	The presence of LH was determined by inspection and palpation of insulin injection sites and was classified into grades 0 through 3: grade 0, no changes; grade 1, visible hypertrophy of fat tissue but palpably normal consistency; grade 2, massive thickening of fat tissue with higher consistency; and grade 3, lipoatrophy
Al Hayek 2016	Clinical (visual inspection and palpation)	LH values were distinguished as follows: grade 0 = no change; grade 1 = visible hypertrophy of fat tissue but with normal consistency on palpation; grade 2 = intensive fat tissue thickening but with firm consistency; and grade 3 = lipoatrophy
Al Jaber 2020	Clinical (visual inspection and palpation)	Palpable LH or a noticeable mass at the insulin injection sites indicated the presence of LH, and the absence of LH indicated a normal injection site.2 LH grades were defined as follows: grade 0, no change; grade 1, visible hypertrophy of fat tissue, but with normal consistency on palpation; grade 2, intensive fat tissue thickening, but with firm consistency; and grade 3, lipoatrophy
Arora 2021	USG (visual inspection and palpation, USG in all patients)	The method consisted of the inspection of each interested area and a thorough palpation technique (slow circular and vertical finger tip movements followed by repeated horizontal attempts on the same spot). Gel was used to facilitate palpation. Lipohypertrophy was graded as following: grade 0, no changes (normal fat texture); grade 1, visible hypertrophy of fat tissue but palpably normal texture; grade 2, massive thickening off at tissue with firm texture compatible with palpable LH; and grade3, lipoatrophy. All 500 patients who were injecting insulin for two years or more were ultrasonographically evaluated. Normal areas near the affected ones were considered as control. The affected areas were examined for their thickness, echogenicity, connective tis-sue distortion, capsule and vascularity. Any abnormally increased subcutaneous thickness associated with heterogenous echotexture was defined as lipohypertrophy. Gentle compression was made throughout examination with no intention to deform and in orderto appreciate echotexture and thickness of tissue abnormalities. We used a diagnostic ultrasound imaging equipment iU 22 (Philipsmedical systems, Netherland) and a 5−17 MHz linear micro array transducer
Barola 2018	Clinical (visual inspection and palpation)	Presence of lipodystrophy at injection sites was ascertained with inspection and palpation techniques. Visible or palpable lump was indicative of lipohypertrophy, while depression at the injection site was suggestive of lipoatrophy. Lipohypertrophy was further confirmed with pinch maneuverer showing non-symmetric bilateral skin folds. Interviewer was not aware about the prior status of lipodystrophy because injection sites were checked only after the collection of all other data
Baruah 2017	Clinical (visual inspection and palpation)	LH vide visual examination and manual palpation
Blanco 2013	Partial USG (visual inspection and palpation, USG in some patients)	Presence, type and location of lipodystrophy was observed by the nurse. In 78 patients with and without clinically detectable lipodystrophy, an ultrasound examination was made of the skin and SC tissue. A portable NanoMaxx® (SonoSite, Bothell,WA, USA) ultrasound unit was used, and a single ultrasound team performed all sonographic procedures. A specific LH ‘echo signature’ was identified, and used to diagnose and describe the lesions
Bochanen 2021	Clinical (visual inspection and palpation)	Participants were thoroughly examined, and all the regions were checked (in sitting and supine position) by visual assessment and palpation (e.g. pinching maneuver).
Cunningham 2013	Clinical (visual inspection and palpation)	Lipohypertrophy was assessed as “present” or “not present”. The presence of a noticeable or palpable lump at the injection site indicated that lipohypertrophy was present
Frid 2016	Clinical (visual inspection and palpation)	To assess the presence of lipohypertrophy (LH), patients were asked: “Do you have any swelling or lumps under the skin at your usual injection sites that have been there for some time (weeks, months, or years)?” Overall, 29.0% answered yes. Nurses were asked to examine all the injection sites for LH both visually and by palpation.
Gentile 2019	Clinical (visual inspection and palpation)	Two trained physicians for each dialysis unit separately identified LH at all sites utilized by insulin-treated patients and, in case of any discrepancy, did it again until final agreement. The LH identification procedure referred to a structured observation and palpation method taking into account further suggestions derived from previously published experience. Briefly, the method consisted of the inspection of each area of interest through direct and tangential light against a dark background and of careful palpation implying slow circular and vertical, progressively deeper finger tip movements followed by repeated horizontal attempts on the same spot. In the present study only LH lesions were considered.

Study	Type of measurement	Lipohypertrophy definition
Gentile 2020	USG (visual inspection and palpation, USG in all patients)	Only trained HCPs with at least 3 years of specific experience performed the protocol, using a US jelly to enhance fingertip sensitivity as previously described and validated. It consisted of (1) the inspection of each area of interest using direct and tangential light against a dark background, taking into account the patient body position during injection, and (2) a thorough palpation technique involving slow circular and vertical fingertip movements followed by repeated horizontal attempts at the same spot. For abdominal examination. patients were lying down and stood up afterward; for thigh examination, they sat with bent legs and feet on the floor. HCPs gently touched the skin at the beginning and then progressively increased the finger pressure. When perceiving harder skin, they performed a pinching maneuver to compare the thickness of suspected LHs to that of the surrounding areas and repeated all maneuvers mentioned above in case of smaller and flatter lesions . High-frequency B-mode US skin scans were performed at all injection sites using the linear 20-MHz probe (Philips HD3) to define single LH features, including size, thickness, and texture, as described elsewhere, and to check palpation results as a secondary goal. Briefly, in our above-mentioned validation studies involving two different blinded operators on the same patients, a 100% consistency in LH identification was already apparent from the intra-operator, inter-operator, and day-to-day operator variation, independently of LH location, volume, extension, texture, or thickness. According to US features, we classified LH areas as described elsewhere into– hyper-type A: iso-hyperechoic with a prevailing fibrotic component; – iso-type B: isoechoic associated with small edema-like islands bordered by fibrous strips; – iso-hypo type C: iso-hypoechoic fiber-free
Gentile 2021	Partial USG (visual inspection and palpation, USG in some patients)	The validated LH identification method was described previously. It consisted of inspecting each area of interest through direct and tangential light against a dark background and a thorough palpation technique implying slow circular and vertical fingertip movements followed by repeated horizontal attempts on the same spot. Healthcare professionals had to touch the skin gently initially and progressively increase finger pressure. They also confirmed the clinical diagnosis through a pinching maneuver to distinguish contiguous areas by thickness and hardness. When needed, smaller and flatter lesions were further identified by ultrasound (US) scanning.
Gentile 2022	USG (visual inspection and palpation, USG in all patients)	All patients underwent accurate evaluation at all their insulin injection sites by expert nurses or doctors certified as experts in the detection, grading, and measurement of LH, including visualization and palpation of the adipose tissue. Emphasis was placed on the need for oblique lighting to aid the visual detection of LH lesions, a warm environment, and a supine position with knees up to relax abdominal muscles. The location and size of LH were confirmed or identified by physical examination by two independent observers (one performed the physical examination and the other reviewed its results). LH presence was confirmed by ultrasound reevaluation as well. High-frequency B-mode ultrasound skin scans took advantage of linear 20 MHz probes (HD3; Philips NV, Amsterdam, the Netherlands) at all injection sites.
Gunhan 2022	Clinical (visual inspection and palpation)	Patients underwent a structured physical examination visual inspection and palpation of all injection sites. Examinations were made in warm rooms (to avoid shivering) with oblique lighting to aid visual inspection, and examiners' hands were washed and warmed. For abdominal examinations, patients were in supine position on the examination table with knees drawn up to relax abdominal musculature. Light to moderate pressure with small sweeps of the finger tips was used to expose LH lesions. When found, lesion dimensions (longest diameter and perpendicular length) were marked with a pen, measured and recorded. The number of lesions in an area was noted, but dimensions only recorded for the largest lesion. For the thigh, patients were examined sitting with knees bent and feet on the floor. The arm and buttock were similarly evaluated (patient standing for the latter), if the patient injected at such sites. Obtained data was evaluated according to presence (LD+) and absence (LD-) of lipodystrophy.
Gupta 2018	Clinical (visual inspection and palpation)	Observation and palpation techniques were used in assessing LH rather than only visual examination. To feel subtle skin thickening, a gel lubricant was used and the hand was stroked firmly over the injection sites with sweeping and undulating movements. LH was graded as grade 0, no changes (normal fat texture); grade 1, visible hypertrophy of fat tissue but palpably normal texture; grade 2, massive thickening of fat tissue with firm texture compatible with palpable LH; and grade 3, lipoatrophy
Hajheydari 2011	Clinical (visual inspection and palpation)	For the evaluation of the lipodystrophy in the injection site, all of the subjects were examined by one specialist physician, through observation and palpation technique
Hauner 1996	Clinical (visual inspection and palpation)	Skin areas of previous and present sites of insulin injection were carefully examined by a trained physician. Lipohypertrophy was defined as visible and/or palpable fatty swelling of varying consistency at the sites of insulin injection. Lipohypertrophy was classified as discrete when tissue swellings did not exceed a size of 3 cm in diameter and of 0.5 cm in height above skin level at one of the injection sites (for both dimensions a rule with a mm scale was used). Marked lipohypertrophy was defined as a skin elevation of larger size
Ji 2014	Clinical (visual inspection and palpation)	The data on lipohypertrophy were obtained through visual examination and palpation by nurses. Nurses from the centers were trained on how to assess bleeding, bruising, and lipohypertrophy (by both observation and palpation) at injection sites before participating in the study.
Ji 2017	Clinical (visual inspection and palpation)	Visual inspection and palpation of all injection sites. Study staff who performed examinations were trained to detect LH, first with models or mannequins, and then with patients known to have LH lesions. For abdominal examinations, patients lay supine in their underwear on the examination table with knees drawn up to relax abdominal musculature. Ultrasound gel was applied to examiners' fingers and the subject's abdomen, and palpation of injection areas performed. Light-to-moderate pressure with small sweeps of the finger tips was used to detect LH lesions. When found, lesion dimensions (longest diameter and perpendicular length) were marked with a pen, measured, and recorded. The number of lesions in an area was noted, but dimensions only recorded for the largest lesion. For the thigh, patients were examined sitting with knees bent and feet on the floor. The arm and buttock were similarly evaluated (patient standing for the latter), if the patient injected at such sites.

Study	Type of measurement	Lipohypertrophy definition
Kamrul-Hasan 2020	Undefined	Undefined
Korkmaz 2021	Only USG (patients with clinical lipohypertrophy were excluded)	The two researchers received clinical training for SSU from a radiologist specialising in SSU. One researcher examined the body parts (legs, arms and abdomen) using the Esaote My Lab60 linear probe with multiple frequencies (6---18 MHz) and recorded the SSU images of each region mentioned above. Both researchers took part in the SSU analysis of the participants to reduce inter-operator variability via video recording. Because of the heterogeneity of the appearance of subcutaneous tissues among individuals, the mid-axis line for the abdomen, just above the elbow for the arm and just above the knee for the leg were examined as non-injected personal control areas. The lesions of patients with LH were compared with lesions described by Kapetulo et al [41]
Kumar 2021	USG (visual inspection and palpation, USG in all patients)	The presence of lipohypertrophy was assessed in patients by clinical examination of the abdomen by inspection and palpation and were graded from 0 to 2 as follows: grade 0: no changes, grade 1: visible hypertrophy of the fat tissue but palpably normal consistency, grade 2: massive thickening of the fat tissue with a higher consistency. All subjects underwent an ultrasound screening of the dermis and subcutaneous tissue of the abdomen for evidence of lipohypertrophy by the radiologist who was blinded to the patient's clinical findings. Ultrasonography was performed using a Philips EPIQ 5G machine, transducer L18-5 broadband linear array working on 18 to 5MHz extended operating frequency. On the ultrasonography, the normal dermis is homogenously hyperechoic when compared with the subdermal fatty tissue and ranges between 1 and 4 mm in thickness. There is a well-defined and regular demarcation between dermis and the subcutis. The subcutaneous tissue offers a hypoechoic background secondary to the fat lobules and a hyperechoic connective web with very thin septa between the lobules. The hyperechoic muscularis fasciae are seen beneath the subcutis layer. Based on the thickness, echogenity, echotexture, delineation between dermis, subcutis and muscularis layers, and subcutis vasculature on ultrasound, the lipohypertrophy was further classified based on the system suggested by Kapeluto and colleagues [41]
Lin 2022	USG (visual inspection and palpation, USG in all patients)	Clinical palpation assessment of LHT: Three senior nurses with a long history of diabetes education was trained to assess each patient for the presence of abdominal LHT. Each patient was randomly palpated by the above three nurses, and the mean of LHT was calculated afterwards. The assessment was performed as follows: (1) the patient was placed in a lying position and instructed to fully relax their abdominal muscles, bend their knees, relax their quadriceps muscles, cross their arms over their chest, and relax their arm muscles, while the examiner took a sitting position for the examination; (2) the examiner used a light source to fully illuminate the area under examination, adjusting the angle so that any subtle elevations or depressions on the skin surface could be seen and using a marker to mark the center of the area with bumps or changes in skin color or hair distribution; (3) when palpation initially revealed a soft, elastic, subcutaneous fatty tissue that eventually changed to tough, rubbery, or inelastic tissue, the exact location of the lesion was marked using a safe skin marker. Ultrasonography to assess LHT: Ultrasound examination of the abdominal skin tissues was performed by two senior ultrasonographers who had been practicing superficial tissue ultrasound for a significant period of time, using a multi-frequency linear probe (L8-18I, 8–18MHz). The examination was performed as follows: (1) the patient was examined in the same position as that used for clinical palpation, and (2) the ultrasound signs at the examination site and the thickness of the subcutaneous adipose tissue in the lesion area and that of the surrounding normal subcutaneous adipose tissues were recorded, and the exact location of the lesion was marked using a safe skin marker. LHT was considered to be present when the specific criteria for the ultrasound diagnosis of LHT were met: the presence of echogenically heterogeneous nodules in the hyperplastic area with differences in echotexture from the surrounding normal tissue and interstitial edema around the hyperplastic nodules, continuous thick fascial tissue or interrupted and distorted thin connective tissue around the hyperplastic nodules, and little or absent neovascularized echogenicity in the hyperplastic nodules.
Luo 2021	USG (visual inspection and palpation, USG in all patients)	Each participant was examined for lipohypertrophy on all injection areas through clinical examination and ultrasound scanning sequentially. Lipohypertrophy was assessed as “clinical lipohypertrophy present,” “clinical lipohypertrophy present,” or “lipohypertrophy absent.” Clinical lipohypertrophy present means that a noticeable or palpable/unpalpable lump at the injection site was found through inspection and palpation. Examinations took place in warm rooms, and the examiner's hands were washed and warmed. The inspection of each injection site was performed using direct and tangential light against a dark background. After a thorough inspection, injection sites' systematic palpation was performed: “slow circular and vertical finger-tip movements followed by repeated horizontal attempts on the same spot.” Examiner was also suggested to perform the pinch maneuver and compare the suspected site's thickness to surrounding areas when perceiving a more hardened skin. Both inspection and palpation were performed in the upright and the lying position. After clinical examination, all participants underwent an ultrasound assessment by a single radiologist. The operator was blinded to participants' responses to survey questions and the clinical examination findings. Ultrasound scanning was performed invariably using the Esaote My Lab60 linear probe multifrequency (6–18MHz). At each injection site, the thickness, echo, and blood flowof subcutaneous tissue and the boundary between subcutaneous tissue and dermis were scrutinized. For each participant, additional scans were performed at non-injection sites, which were symmetric with the injection sites through the mid-axial line, as a person-specific control. The presence of lipohypertrophy on ultrasonography was determined using the criteria described by Kapeluto et al [41]. A lesion was considered as lipohypertrophy if it was located in the subcutaneous tissue and met at least four of the following five criteria: “1) well-circumscribed either by hyperechoic fociwith defined borders or a nodular shapewith a hypoechoic halo, 2) heterogeneous in echotextur The existence of a lesion meeting ultrasonic criteria for lipohypertrophy that was not detected on clinical examination indicated that subclinical lipodystrophy was present. Participants were divided into three groups based on the results of clinical examination and ultrasound scanning: “clinical lipohypertrophy group”, “subclinical lipohypertrophy group”, or “lipohypertrophy absent group”. Clinical lipohypertrophy group: patients had one or more clinical lipohypertrophy lesions and had no subclinical lipohypertrophy. Subclinical lipohypertrophy group: patients had one or more subclinical lipohypertrophy lesions and had no clinical lipohypertrophy. Lipohypertrophy absent group: patients had neither clinical lipohypertrophy nor subclinical lipohypertrophy.
Nawaz 2023	Undefined	Undefined

Study	Type of measurement	Lipohypertrophy definition
Omar 2011	Clinical (visual inspection and palpation)	Lipohypertrophy was graded as following: grade 0 = no changes; grade 1 = visible hypertrophy of fat tissue but palpably normal consistency; grade 2 = massive thickening of fat tissue with firm consistency; and grade 3 = lipoatrophy
Pahuja 2019	Clinical (visual inspection and palpation)	The trained interviewer also examined the patient for LH through observation and palpation of insulin injection sites
Pozzuoli 2018	Clinical (visual inspection and palpation)	The presence of LH was assessed by inspection and palpation of injection sites. Patients underwent a careful clinical examination of the skin areas corresponding to the sites of injection by well-trained healthcare professionals, following a standardized approach. Each interested area was inspected using direct and tangential light against a dark background, and a meticulous palpation was performed (slow circular and vertical finger tip movements followed by repeated horizontal attempts on the same spot). Gentle touching of the skin at the beginning was followed by progressive increase in finger pressure. The pinch maneuver was also performed when a harder skin was perceived. Small and flat lesions were investigated by repeating all above mentioned palpation maneuvers at least three times in a row
Saeed 2022	Clinical (visual inspection and palpation)	Lipohypertrophy was determined by clinical examination. All the injection sites were inspected from various angels using tangential light in both upright and supine position. The relevant site was also palpated and an area of skin suspected for LH was pinched to identify difference in thickness or texture from the surrounding skin.
Saez de Ibarra 1998	Clinical (visual inspection and palpation)	Lipohypertrophy was defined as a visible or palpable increase of subcutaneous fat at the sites of insulin injection. Evaluations were performed by a trained diabetes nurse
Singha 2021	USG (visual inspection and palpation, USG in all patients)	Insulin injection sites were examined independently by two different observers by inspection using direct and tangential lighting and palpation. In all participants with LH, its presence was further confirmed by ultrasonographic examination using a Samsung Sono Ace R7 5–12 MHz linear probe by a single operator. The presence of heterogeneously echogenic areas with diminished vascularity in the subcutaneous fat plane with corresponding deep deflection of the muscular band was used as the ultrasonographic criteria for LH
Strollo 2016	Clinical (visual inspection and palpation)	LH was looked for at all insulin injection sites using a previously described validated methodology: when filling in their questionnaires patients also indicated all sites they used and the medical staff checked them for the presence of skin lesions according to a structured protocol.
Sürücü 2018	Clinical (visual inspection and palpation)	The presence and location of lipohypertrophy was assessed by certified diabetes nurses using inspiration and palpation method. Lipohypertrophy evaluation requires both inspection and palpation examination of the injection sites. For this reason, the health care worker should first evaluate the injection site visually, then mass status in the swollen site with the thumb and forefinger.2,6 The mass is specified as “present” or “not present”
Thewjitcharoen 2020	Partial USG (visual inspection and palpation, USG in some patients)	Experienced diabetes nurse educators, skilled in performing observation and palpation techniques, evaluated the presence of LH or LA in all patients. The inspection of injection site was done carefully with using direct and tangential light and then a gentle palpation technique involving fingertip movements followed by pinching maneuver in the suspected LH area. A clinical grading of LH was applied (Grade 1: lipohypertrophy without visible skin lesion but increased palpable density of subcutaneous tissue; Grade 2: severe hypertrophy with increased density of the injection site). Lipoatrophy at any injection site was noted separately. Ultrasonographic studies were performed by an experienced radiologist in some patients with equivocal area of LH or some patients who had concurrent LH and LA.
Tsadik 2018	Clinical (visual inspection and palpation)	Observation and palpation techniques were used in assessing lipodystrophy in these diabetics. Lipodystrophy was assessed as “present” or “not present”. The presence of a noticeable or palpable lump at the injection site indicated that lipodystrophy was present. Accordingly, lipodystrophy was defined to have different grades based on morphology and pathogenesis. Grade 1 = a small protruding lipohypertrophy, grade 2 = large lipohypertrophy, and grade 3 = lipoatrophy characterized by subcutaneous fatty tissue atrophy. Lipodystrophy examination was performed by a nurse

Table 11. Definitions of hypoglycemia in the included studies

Study	Hypoglycemia definition
Al Hayek 2016	Hypoglycemia was defined as the occurrence of one or more symptoms of hypoglycemia (such palpitations, tiredness, sweating, strong hunger, dizziness, and tremor) and a confirmed blood glucose level of B60 mg/dL (3.3mM/L). Frequent unexplained hypoglycemia was defined as having a hypoglycemic episode one or more times a week in the absence of a definable precipitating event, such as a change in medication, diet, or activity
Arora 2021	Undefined
Baruah 2017	Hypoglycemia was defined as the occurrence of one or more typical symptoms, such as palpitations, tiredness, sweating, strong hunger, dizziness, and tremor, which were reverted by oral intake of carbohydrate or parenteral administration of glucose with or without a confirmed blood glucose reading of ≤70 mg/dL, by glucometer or incidental laboratory report
Blanco 2013	'Hypoglycaemia' was defined as the occurrence of one or more symptoms of hypoglycaemia (such as palpitations, tiredness, sweating, strong hunger, dizziness and tremor) and a confirmed blood glucose meter readingof ≤ 60 mg/dL (3.3 mM/L). 'Frequent unexplained hypogly-caemia' was defined as having a hypoglycaemic episode one or more times a week in the absence of a definable precipitating event, such as a change in medication, diet or activity
Bochanen 2021	Clinically important hypoglycaemia was defined as having symptoms of hypoglycaemia and confirmed blood glucose reading ≤54 mg/dl, in accordance with the international consensus. Moreover, to limit the risk of false low iCGM values, participants using iCGM were instructed to confirm the hypoglycaemic reading with a capillary blood sample (SMBG). Unexplained hypoglycaemia was defined as hypoglycaemia occurring in the absence of a definable precipitating event such as a change in medication, diet or activity. Severe hypoglycaemia was defined as a hypoglycaemic event requiring help from a third party. All hypoglycaemic events were collected from electronic medical records, digitally recorded in the SMBG or iCGM software (LibreView (Abbott Freestyle Libre isCGM))
Frid 2016	We defined hypoglycemia as the occurrence of at least 1 symptom of low blood sugar levels (eg, palpitations, tiredness, sweating, hunger, dizziness, or tremor) and a confirmed blood glucose meter reading of 60 mg/dL or less (to convert to mmol/L, multiply by 0.0555). Because there is no consistent definition of hypoglycemia across the board, we chose this strict one to increase specificity. Frequent unexplained hypoglycemia was defined as hypoglycemia occurring 1 or more times weekly in the absence of a definable precipitating event, such as a change in medication or dose, diet, or activity.
Gentile 2019	HYPO was defined according to American Diabetes Association (ADA) statements, i.e., the occurrence of one or more symptoms of hypoglycemia (including palpitations, tiredness, sweating, hunger, dizziness, and tremor) and a confirmed blood glucose (BG) reading B 70 mg/dL. Frequent unexplained hypoglycemia was defined as having HYPO at least once a week in the absence of any identified precipitating event including changes in insulin dosage, diet composition, or amount of physical activity. HYPOs were further defined as severe (SH; BG\50 mg/dL) and non-severe (NSH; 50[BG\70 mg/dL)
Gentile 2020	We defined (1) severe hypoglycemia (SeH) according to the 2019 ADA guideline as an episode leading to unconsciousness or requiring assistance by a third person or associated with blood glucose levels\54 mg/dl (3.0 mmol/l) or in the 56–70 mg/dl range; (2) symptomatic hypoglycemia (SyH) as an episode characterized by at least one of the following symptoms resolving with food or sugary drink ingestion: palpitations, tremors, sweating, shakiness, irritability, concentration troubles, dizziness, hunger, blurred vision, confusion, tachycardia, or difficulty moving; (3) frequent unexplained hypoglycemia (UH) as the occurrence of hypoglycemic episodes at weekly intervals or more, in the absence of any identified precipitating events, such as changes in insulin dosage, diet, or amount of physical activity
Gentile 2021	Hypoglycemia was defined as the occurrence of one or more symptoms of hypoglycemia (such as palpitations, tiredness, sweating, hunger, dizziness, and tremor) and a confirmed blood glucose meter reading of 60 mg/dL or less, as previously described several times. Frequent unexplained hypo was defined as having one or more hypos a week in the absence of any changes in medication, diet, or physical activity.
Gentile 2022	HYPOs were defined according to American Diabetes Association (ADA) statements. Briefly, a HYPO consisted of one or more symptoms (palpitations, tiredness, sweating, hunger, dizziness, and tremor) confirmed by a blood glucose (BG) reading B 70 mg/dL. It was further classified as SeH (BG B 50 mg/dL) and SyH (BG 50–70 mg/dL). Finally, a HYPO was defined as unexplained in the absence of any identified precipitating event, including changes in insulin dosage, diet composition, or amount of physical activity.
Gupta 2018	Hypoglycemia was defined as the occurrence of more than one symptom of low sugar (palpitations, tiredness, sweating, strong hunger, dizziness, tremor, etc.) and a confirmed BG meter reading of <70 mg/dL (3.9 mmol/L). Frequent UH was defined as hypoglycemia occurring one or more times a week in the absence of definable precipitating event such as change in medication, diet, or activity
Ji 2017	Hypoglycemia is self-reported within the past 6 months
Kamrul-Hasan 2020	Hypoglycemia was defined as plasma glucose <3.9 mmol/L with or without hypoglycaemic symptoms.
Nawaz 2023	Undefined
Pozzuoli 2017	Hypoglycemic episodes were classified as severe (unconsciousness or episode requiring assistance by third party), confirmed symptomatic (classical symptoms of hypoglycemia associated with a SMBG value < 70 mg/dL or relief of symptoms after ingestion of a drink containing glucose), asymptomatic (occasional detection of a SMBG value < 70 mg/dL in the absence of hypoglycemia symptoms). Patients were asked to report the occurrence of severe hypoglycemic episodes in the previous 12 months and the occurrence of symptomatic and asymptomatic episodes in the previous 3 months
Singha 2021	Hypoglycemia was defined as a documented capillary blood glucose <70 mg/dL, with or without hypoglycemic symptoms. The previous three months' SMBG readings were checked for documented hypoglycemia (with or without accompanying hypoglycemic symptoms). The number of hypoglycemic episodes that could not be explained by missed or unusually small meal and/or unaccustomed physical activity were recorded

Study	Hypoglycemia definition
Strollo 2016	Hypo was defined as the occurrence of one or more symptoms of hypoglycaemia (such as palpitations, tiredness, sweating, hunger, dizziness and tremor) and a confirmed blood glucose (BG) meter reading of ≤70 mg/dL, according to ADA statements. Frequent unexplained hypoglycaemia was defined as having a Hypo at least once a week in the absence of any identified precipitating event, such as changes in insulin dosage, diet composition or amount of physical activity. Hypos were further defined as severe (SH; BG<50 mg/dl), and non-severe (NSH; 50th>BG<70 mg/dl)
Sürücü 2018	Hypoglycemia and unexplained hypoglycemia were obtained from the patients' own statements. Hypoglycemia: Defined as presence of one or more symptoms associated with hypoglycemia (palpitations, fatigue, sweating, hunger, dizziness and tremor) and confirmation of blood glucose level of ≤ 60 mg/ dl on blood glucose meter. Unexplained recurrent hypoglycemia: Defined as the development of hypoglycemic episodes at least once or more than one in a week without an identifiable triggering event, such as drug treatment, dietary or activity changes
Thewjitcharoen 2020	Severe hypoglycemia was defined according to a joint position statement of the American Diabetes Association (ADA) and International Hypoglycemia Study Group (IHSG) as an episode leading to unconsciousness or requiring assistance by a third person. Only clinically important hypoglycemia (Level 2 hypoglycemia, a glucose level of <54 mg/dL with typical hypoglycemic symptoms) was collected. Unexplained hypoglycemia was defined as the occurrence of hypoglycemia not related to a mismatch of meal and activities
Tsadik 2018	Hypoglycemia was defined as the occurrence of one or more symptoms of hypoglycemia (such as poor concentration, irritability, palpitation, tiredness, sweating, strong hunger, dizziness, and tremor) and a confirmed blood glucose meter reading of less than or equal to 70 mg/dL. “Frequent unexplained hypoglycemia” was defined as having one or more hypoglycemic episode per week in the absence of a definable precipitating event, such as a change in medication, diet, or activity

Table 12. Definitions of glycemic variability (GV) in the included studies

Study	Glycemic variability definition
Blanco 2013	As ‘glycaemic variability’ has no universally agreed-upon definition, in our study patients were so classified if they had oscillations of blood glucose values from < 60 mg/dL (3.3 mM/L) to > 250 mg/dL (13.9 mM/L) at least three times a week in an unpredictable and unexplained fashion, and if there was evidence that such a pattern had been present for at least6 months previously
Bochanen 2021	Increased glucose variability was defined as the occurrence of at least three times a week glucose values evolving from <60 mg/dl (3.3 mmol/L) to >250 mg/ dl (13.9 mmol/L) or vice versa (i.e. a delta >190 mg/dl or 10.6 mmol/L) in an unpredictable and unexplained manner and evidence of such a pattern during the previous 6 months
Frid 2016	We defined glycemic variability as the presence of blood glucose oscillations from less than 60 mg/dL to more than 250 mg/dL at least 3 times a week in an unpredictable and unexplained manner and evidence that such a pattern has been present for at least the previous 6 months.
Gentile 2019	GV was investigated by a validated questionnaire already used in previous studies and, as a result of the lack of universally accepted criteria, was defined as unpredictable and unexplained shifts from \60 mg/dL to 250 mg/dL occurring one or more times on the same day or on different days within 1 week during the previous month. Since a large glycemic variability is typically observed between days on and off dialysis, the average of the widest glycemic variations occurring on three dialysis days and three dialysis-free days within 1 week was considered.
Gentile 2020	We defined glycemic variability (GV) as the mean blood glucose fluctuation occurring over the observation period in which patients controlled capillary pre- and 2-h-postprandial levels at each mealtime and midnight. They also had to check their capillary blood glucose (BG) level whenever feeling any symptoms suggestive of hypoglycemia. All electro-medical devices used to evaluate BG were ISO-directive certified and periodically validated and verified by the DCs’ diabetes nurses. In the absence of any user-friendly unanimously accepted clinical method, GV was investigated through a validated questionnaire and defined as high when BG levels swung consistently, inexplicably, and unpredictably from <60 to >250 mg/dl at least once a week over the 3 months preceding enrolment and at least 3 weeks within the first and second trimester of the study
Gentile 2021	GV was classified as unpredictable and unexplained self monitored blood glucose (SMBG)-based glucose oscillations ranging from less than 60 to more than 250 mg/dL at least three times a week for at least 6 months
Gupta 2018	GV was diagnosed in the presence of BG oscillations from <70 mg/dL (3.9 mmol/L) to >250 mg/dL (13.8 mmol/L), at least three times a week in an unpredictable and unexplained manner with such a pattern present for at least the previous 6 months.
Saez de Ibarra 1998	Glycaemic profile was considered irregular or unstable when more than two glycaemic fluctuations [hypo/hyperglycaernia] per week were unexplained

Table 13. Definitions of CGM data in the included studies

Study	Glycemic variability definition
Gentile 2022	Undefined
Gunhan 2022	Fasting plasma glucose levels (FPG) were measured using an enzymatic UV test (hexokinase method)
Gupta 2018	Ambulatory glucose profile (AGP) monitoring was performed on 10 subjects (*10%) with LH and age >18 years after obtaining their consent. Freestyle Libre Pro flash glucose monitoring system (Abbott Diabetes Care, Mumbai, India) was used to construct the profile. The Libre Pro technology uses an electrochemical sensor that measures interstitial glucose every 15 min for 14 days with a range of 40–500 mg%. The sensor was implanted in the left arm using aseptic techniques. Subjects were asked to inject their regular insulin doses into LH for the first 7 days and then into normal tissue sites (without LH) for the next 7 days. GV was studied by calculating the mean glucose, mean amplitude of glycemic excursions (MAGEs), and continuous overlapping net glycemic action (CONGA). MAGE is the arithmetic mean of differences between consecutive peaks and nadirs of glucose readings, which was manually calculated by taking 24-h glucose values from the 2nd day compared with 24-h glucose readings from the 14th day. First and 15th days were not considered, as they often have incomplete readings. Similarly, the CONGA metric calculates a difference between the current BG level and readings obtained in hours (days) earlier and then calculates the standard deviation of the differences
Lin 20222	CGMS (Medtronic, Dublin, Ireland) was used to measure the blood glucose levels in the morning after an overnight fast, 2 h after breakfast, before lunch, 2 h after lunch, before dinner, 2 h after dinner, and at 10:00 p.m. From here, the patients' blood glucose fluctuations were evaluated. The intra-day blood glucose fluctuation index was calculated as follows: largest amplitude of glycemic excursion, which is the difference between the maximum and minimum values of blood glucose in 1 d; mean blood glucose, which is the mean of the blood glucose values in 1 d; the standard deviation of blood glucose, which is the standard deviation of the blood glucose values in 1 d; and postprandial glucose excursion, which is the mean of the difference between the blood glucose values 2 h after three meals and the corresponding pre-meal blood glucose values

Table 14. Definitions of hyperglycemia in the included studies

Study	Hyperglycemia definition
Kamrul-Hasan 2020	Hyperglycemia was defined as plasma glucose >13.9 mmol/L)
Nawaz 2023	Undefined

Table 15. Definitions of HbA1c in the included studies

Study	HbA1c definition
Abujbara 2022	Glycosylated hemoglobin (HbA1c) level and anthropometric measurements were extracted from the medical records.
Al Jaber 2020	Undefined
Arora 2021	Undefined
Barola 2018	Glycated hemoglobin (HbA1c) values measured within past 2 weeks were documented
Baruah 2017	HbA1c level at registration or either before or after 3 weeks of the 1 point of contact
Bochanen 2021	Undefined
Frid 2016	Undefined
Gentile 2019	Undefined
Gentile 2020	Undefined
Gentile 2021	Undefined
Gentile 2022	Undefined
Gunhan 2022	Biochemical results measured in the last 3 months were recorded from the patients' files. HBA1c was analyzed with high-performance liquid chromatography in Premier Hb9210 (Trinity Biotech, USA)
Gupta 2018	Undefined
Hajheydari 2011	HbA1C was measured by HPLC (High Performance Liquid Chromatography) method with hb gold kit prepared by Drew Scientific Itd Company, UK
Hauner 1996	The laboratory variables selected for the study included glycated hemoglobin (HbAIC) measured by HPLC
Ji 2014	The demographic data and HbA1c were drawn from patients' medical records within 3 months
Ji 2017	Undefined
Kamrul-Hasan 2020	The most recent glycated haemoglobin (HbA1c) results (within the previous 3 months of data collection) were collected from their treatment records
Korkmaz 2022	Undefined
Kumar 2021	Hba1c measured by the high-performance liquid chromatography method (HPLC) within the past 3 months was taken for the analysis.
Luo 2021	Participants underwent HbA1c testing by a Clover A1c analyzer (D10 hemoglobin testing system Specifications)
Omar 2011	Average of the last 4 readings
Pahuja 2019	Undefined
Pozzuoli 2018	The most recent HbA1c (%) value in the previous 4 months was registered. HbA1c was measured by high performance liquid chromatography using diabetes control and complications trial-aligned methods
Singha 2021	HbA1c was measured using the ion-exchange high-performance liquid chromatography method using the Bio-Rad D 10 program
Strollo 2016	Undefined
Sürücü 2018	A1C values of the patient for the last three months were obtained from the medical records
Thewjitcharoen 2020	Clinical parameters including the most recent glycated hemoglobin (A1C) value in the previous 3 months, were all recorded and analyzed

Table 16. Definitions of uncontrolled glycemia (HbA1c ≥7 or >7) in the included studies

Study	Uncontrolled glycemia definition

Study	Uncontrolled glycemia definition
Abujbara 2022	HbA1c ≥7%
Al Ajlouni 2015	HbA1c ≥ 7%
Al Hayek 2016	HbA1c ≥ 7%
Al Jaber 2020	HbA1c ≥ 7%
Arora 2021	HbA1c ≥7%
Ji 2017	HbA1c ≥7%
Korkmaz 2022	HbA1c >7%
Kumar 2021	HbA1c >7%
Luo 2021	HbA1c ≥ 7%%
Pahuja 2019	HbA1c ≥7%
Sürücü 2018	HbA1c ≥7%

Table 17. Definitions of daily insulin dose in the included studies

Study	Uncontrolled glycemia definition
Al Jaber 2020	Undefined
Al Hayek 2016	Undefined
Arora 2021	Undefined
Barola 2018	Undefined
Baruah 2017	Undefined
Blanco 2013	Undefined
Bochanen 2021	Undefined
Cunningham 2013	Undefined
Frid 2016	Undefined
Gentile 2019	Undefined
Gentile 2020	Undefined
Gentile 2021	Undefined
Gentile 2022	Undefined
Gunhan 2022	Undefined
Gupta 2018	Undefined
Hauner 1996	Undefined
Ji 2017	Undefined
Korkmaz 2021	Undefined
Kumar 2021	Undefined
Lin 2022	Undefined
Luo 2021	Undefined
Omar 2011	Undefined
Pahuja 2019	Undefined
Pozzuoli 2017	Undefined
Saeed 2022	Undefined
Singha 2021	Undefined
Strollo 2016	Undefined
Thewjitcharoen 2020	Undefined
Tsadik 2018	Undefined

Table 18. Results in individual studies – unexplained hypoglycemia

Study	LH+, n/N (%)	LH-, n/N (%)	pOR (95% CI)	p
Al Hayek 2016	28/83 (34%)	12/91 (13%)	3.35 (1.57–7.16)	<0.05
Blanco 2013	108/277 (39%)	9/153 (6%)	10.22 (5.00–20.91)	<0.01
Bochanen 2021	51/92 (55%)	17/54 (32%)	2.71 (1.34–5.49)	0.045
Frid 2016	NA	NA	NA	<0.05
Gentile 2020	595/718 (83%)	109/509 (21%)	17.75 (13.32–23.66)	0.0001
Gentile 2021	157/360 (44%)	60/420 (14%)	4.64 (3.29–6.54)	<0.001
Gupta 2018	93/97 (96%)	23/42 (55%)	19.21 (5.96–61.94)	<0.0001
Strollo 2016	290/298 (97%)	27/89 (30%)	83.24 (36.11–191.90)	<0.0001
Sürücü 2018	80/191 (42%)	26/245 (11%)	6.07 (3.69–9.99)	<0.001
Thewjitcharoen 2020	4/149 (3%)	1/251 (<1%)	6.90 (0.76–62.29)	0.045
Tsadik 2018	29/103 (28%)	27/73 (37%)	0.67 (0.35–1.27)	>0.05

NA – not available

Table 19. Results in individual studies – symptomatic hypoglycemia

Study	LH+, n/N (%)	LH-, n/N (%)	pOR (95% CI)	p
Baruah 2017	8/94 (9%)	49/654 (7%)	1.15 (0.53–2.51)	>0.05
Gentile 2019	35/169 (21%)	110/127 (87%)	0.04 (0.02–0.08)	0.00016
Gentile 2020	701/718 (98%)	339/509 (67%)	20.68 (12.35–34.61)	0.001
Ji 2017	118/213 (55%)	119/188 (63%)	0.72 (0.48–1.08)	0.108
Pozzuoli 2017	105/151 (70%)	128/201 (64%)	1.30 (0.83–2.04)	0.31
Sürücü 2018	118/191 (62%)	74/245 (30%)	3.74 (2.51–5.57)	<0.001

Table 20. Results in individual studies – severe hypoglycemia

Study	LH+, n/N (%)	LH-, n/N (%)	pOR (95% CI)	p
Bochanen 2021	14/92 (15%)	9/54 (17%)	0.90 (0.36–2.24)	>0.05
Pozzuoli 2017	13/151 (9%)	7/201 (3%)	2.61 (1.02–6.71)	0.15

Table 21. Results in individual studies – overall hypoglycemia

Study	LH+, n/N (%)	LH-, n/N (%)	pOR (95% CI)	p
Gentile 2022	289/487 (59%)	46/673 (7%)	19.89 (14.02–28.23)	0.0001
Kamrul-Hasan 2020	36/78 (46%)	275/769 (36%)	1.54 (0.96–2.46)	0.084
Nawaz 2023	58/83 (70%)	55/280 (20%)	9.49 (5.45–16.51)	<0.05

Table 22. Results in individual studies – episodes of hypoglycemia per month

Study	LH+		LH-		MD (95% CI)	p
	N	Mean (SD)	N	Mean (SD)		
Gentile 2019	169	2.0 (3.0)	127	12.0 (5.0)	-10.00 (-10.98–-9.02)	0.0057
Singha 2021	46	4.2 (2.3)	45	2.2 (1.4)	2.00 (1.22–2.78)	<0.001

Table 23. Results in individual studies – non-symptomatic hypoglycemia

Study	LH+, n/N (%)	LH-, n/N (%)	pOR (95% CI)	p
Pozzuoli 2017	55/151 (36%)	45/201 (22%)	1.99 (1.24–3.17)	0.02

Table 24. Results in individual studies – unexplained or severe hypoglycemia

Study	LH+, n/N (%)	LH-, n/N (%)	pOR (95% CI)	p
Arora 2021	NA	NA	NA	0.048

NA – not available

Table 25. Results in individual studies – HbA1c (%)

Study		LH+		LH-		MD (95% CI)	p
		N	Mean (SD)	N	Mean (SD)		
Al Jaber 2020		80	9.53 (1.60)	122	9.14 (1.70)	0.39 (-0.07–0.86)	0.103
Arora 2021		290	10.53 (1.73)	210	8.95 (1.53)	1.58 (1.29–1.86)	<0.001
Barola 2018		231	10.00 (2.70)	141	9.20 (2.40)	0.80 (0.27–1.33)	0.007
Baruah 2017		94	9.34 (2.28)	654	9.08 (2.10)	0.26 (-0.23–0.75)	>0.05
Bochanen 2021		92	7.50 (1.10)	54	7.40 (1.20)	0.10 (-0.29–0.49)	NA
Frid 2016		2205	8.85 (2.70)	4795	8.30 (1.90)	0.55 (0.43–0.67)	<0.001
Gentile 2019		169	9.20 (1.60)	127	8.10 (2.30)	1.10 (0.63–1.57)	0.0073
Gentile 2020		718	9.60 (1.10)	509	7.50 (1.10)	2.10 (1.98–2.22)	NA
Gentile 2021		360	8.00 (1.20)	420	7.70 (1.30)	0.30 (0.12–0.48)	<0.001
Gentile 2022		487	8.60 (1.60)	673	7.30 (1.00)	1.30 (1.14–1.46)	0.01
Gunhan 2022		98	8.50 (1.70)	247	9.30 (7.80)	-0.80 (-1.83–0.23)	0.23
Gupta 2018		97	9.62 (2.13)	42	9.84 (2.66)	-0.22 (-1.13–0.69)	0.643
Hajheydari 2011		35	9.50 (2.20)	185	8.70 (1.90)	0.80 (0.02–1.58)	<0.03
Hauner 1996		66	8.90 (1.80)	213	8.80 (1.70)	0.10 (-0.39–0.59)	>0.05
Ji 2014		134	7.87 (1.53)	246	8.17 (4.41)	-0.30 (-0.91–0.31)	0.602
Ji 2017		213	8.20 (1.80)	188	7.70 (1.50)	0.50 (0.18–0.82)	0.003
Kamrul-Hasan 2020		78	10.00 (2.20)	769	9.50 (2.00)	0.50 (-0.01–1.01)	<0.001
Korkmaz 2022		119	8.70 (3.30)*	17	10.70 (5.00)*	NA	0.113
Kumar 2021	Clinical	60	9.52 (2.14)	28	8.46 (1.99)	1.06 (0.15–1.97)	0.02
	+ Subclinical	79	9.12 (2.53)	8	8.68 (1.39)	0.44 (-0.67–1.55)	0.03
Luo 2021	Clinical LH	207	8.65 (1.78)	46	7.42 (1.73)	1.23 (0.67–1.79)	NA
	Subclinical LH (USG)	63	8.55 (1.70)	46	7.42 (1.73)	1.13 (0.48–1.78)	NA
	Both	270	8.63 (1.76)	46	7.42 (1.73)	1.21 (0.67–1.75)	NA
Omar 2011		62	8.50 (2.00)	51	8.50 (2.30)	0.00 (-0.80–0.80)	0.837
Pahuja 2019		65	8.30 (NA)	31	7.90 (NA)	0.40 (-0.26–1.06)	0.236
Pozzuoli 2018		151	7.70 (1.40)	202	7.70 (1.30)	0.00 (-0.29–0.29)	0.83
Singha 2021		46	9.00 (2.60)	45	7.70 (1.10)	1.30 (0.48–2.12)	<0.001
Strollo 2016		298	8.30 (1.20)	89	7.50 (1.10)	0.80 (0.53–1.07)	<0.001
Thewjitcharoen 2020		149	7.80 (1.50)	251	7.90 (1.70)	-0.10 (-0.42–0.22)	0.545

*Median (IQR). NA – not available.

Table 26. Results in individual studies – uncontrolled glycemia (HbA1c >7% or ≥7%)

Study		LH+, n/N (%)	LH-, n/N (%)	pOR (95% CI)	p
Abujbara 2022		352/415 (85%)	370/433 (85%)	0.95 (0.65–1.39)	NA
Al Ajlouni 2015		389/408 (95%)	593/681 (87%)	3.04 (1.82–5.07)	<0.001
Al Hayek 2016		72/83 (87%)	49/91 (54%)	5.61 (2.63–11.96)	<0.05
Al Jaber 2020		76/80 (95%)	108/122 (89%)	2.46 (0.78–7.77)	0.114
Arora 2021		246/248 (99%)	173/185 (94%)	8.53 (1.89–38.60)	<0.001
Ji 2017		160/213 (75%)	116/190 (61%)	1.93 (1.26–2.95)	0.024
Korkmaz 2022		98/119 (86%)	16/17 (94%)	0.29 (0.04–2.32)	0.697
Kumar 2021		50/60 (83%)	23/28 (82%)	1.09 (0.33–3.54)	NA
Luo 2021	Clinical LH	171/207 (83%)	24/46 (52%)	4.35 (2.20–8.60)	<0.001
	Subclinical LH (USG)	56/63 (89%)	24/46 (52%)	7.33 (2.76–19.46)	
	Both	227/270 (84%)	24/46 (52%)	4.84 (2.49–9.40)	
Pahuja 2019		57/65 (88%)	23/31 (74%)	2.48 (0.83–7.39)	0.251

Study	LH+, n/N (%)	LH-, n/N (%)	pOR (95% CI)	p
Sürücü 2018	187/191 (98%)	184/245 (75%)	15.50 (5.52–43.49)	<0.001

Table 27. Results in individual studies – glycemic variability (dich.)

Study	LH+, n/N (%)	LH-, n/N (%)	pOR (95% CI)	p
Blanco 2013	136/277 (49%)	10/153 (7%)	13.79 (6.97–27.31)	<0.01
Bochanen 2021	65/92 (71%)	29/54 (54%)	2.08 (1.03–4.17)	>0.05
Frid 2016	NA	NA	NA	<0.05
Gentile 2021	224/360 (62%)	140/420 (33%)	3.29 (2.45–4.42)	<0.001
Gupta 2018	91/97 (94%)	24/42 (57%)	11.38 (4.07–31.79)	<0.0001
Saez de Ibarra 1998	41/69 (59%)	14/59 (24%)	4.71 (2.18–10.15)	<0.001

NA – not available.

Table 28. Results in individual studies – glycemic variability (mg/dL)

Study	LH+		LH-		MD (95% CI)	p
	N	Mean (SD)	N	Mean (SD)		
Gentile 2019	169	310 (88)	127	166 (32)	144.00 (129.61–158.39)	0.0068
Gentile 2020	718	287 (76)	509	198 (54)	89.00 (81.73–96.27)	0.001

Table 29. Results in individual studies – daily insulin dose (insulin units)

Study		LH+		LH-		MD (95% CI)	p
		N	Mean (SD)	N	Mean (SD)		
Al Jaber 2020		80	77.33 (32.70)	122	58.95 (35.00)	18.38 (8.90–27.86)	<0.0001
Barola 2018		231	38.30 (16.90)	141	44.80 (19.20)	-6.50 (-10.35–-2.65)	0.001
Baruah 2017		94	35.51 (18.45)	654	32.95 (18.21)	2.56 (-1.42–6.54)	>0.05
Blanco 2013		277	56.00 (26.90)	153	41.00 (24.10)	15.00 (10.04–19.96)	<0.001
Bochanen 2021		92	69.00 (44.20)	54	50.80 (30.80)	18.20 (5.99–30.41)	0.039
Cunningham 2013		28	54.30 (33.10)	27	44.90 (18.40)	9.40 (-4.69–23.49)	0.197
Frid 2016		2192	55.20 (33.00)	4889	45.10 (31.50)	10.10 (8.46–11.74)	<0.001
Gentile 2019		169	35.50 (8.80)	127	32.80 (9.70)	2.70 (0.55–4.85)	0.237
Gentile 2020		718	54.90 (8.20)	509	42.30 (10.00)	12.60 (11.54–13.66)	0.001
Gentile 2021		360	49.00 (24.00)	420	43.00 (27.00)	6.00 (2.42–9.58)	<0.001
Gentile 2022		487	65.50 (10.60)	673	53.60 (9.50)	11.90 (10.72–13.08)	<0.05
Gunhan 2022		98	119.00 (96.00)	247	95.00 (61.00)	24.00 (3.53–44.47)	0.0063
Gupta 2018		97	42.87 (18.57)	42	40.37 (14.11)	2.50 (-3.15–8.15)	0.401
Hauner 1996		66	49.00 (16.00)	213	45.00 (15.00)	4.00 (-0.35–8.35)	>0.05
Ji 2017		213	38.10 (20.10)	188	27.10 (14.30)	11.00 (7.61–14.39)	<0.0001
Korkmaz 2021		119	64.00 (38.00)*	17	46.00 (28.00)*	NA	0.018
Kumar 2021	Clinical	60	61.60 (23.13)	28	41.89 (17.93)	19.71 (10.86–28.56)	0.01
	+Subclinical	79	58.82 (25.16)	9	44.78 (23.45)	14.04 (-2.25–30.33)	0.05
Luo 2021	Clinical LH	207	34.70 (15.62)	46	30.43 (13.35)	4.27 (-0.14–8.86)	NA
	Subclinical LH (USG)	63	27.81 (14.44)	46	30.43 (13.35)	-2.62 (-7.87–2.63)	NA
	Both	270	33.09 (15.60)	46	30.43 (13.35)	2.66 (-1.62–6.94)	NA
Pahuja 2019		64	43.97 (NA)	29	33.55 (NA)	10.42 (1.50–19.34)[c]	0.022
Pozzuoli 2017		151	48.80 (25.90)	202	37.80 (21.40)	11.00 (5.92–16.08)	<0.0001
Singha 2021		46	39.00 (28.00)*	45	38.00 (28.00)*	NA	0.751
Strollo 2016		298	49.00 (18.00)	89	41.00 (19.00)	8.00 (3.55–12.45)	NA
Thewjitcharoen 2020		149	43.00 (24.00)	251	41.00 (25.00)	2.00 (-2.94–6.94)	0.439

*Median (IQR). NA – not available.

Table 30. Results in individual studies – daily insulin dose adjusted to body weight (insulin units/kg)

Study		LH+		LH-		MD (95% CI)	p
		N	Mean (SD)	N	Mean (SD)		
Barola 2018		231	0.95 (0.37)	141	0.99 (0.38)	-0.04 (-0.12–0.04)	0.402
Baruah 2017		94	0.55 (0.25)	654	0.51 (0.27)	0.04 (-0.01–0.09)	>0.05
Gentile 2019		169	0.48 (0.36)	127	0.40 (0.41)	0.08 (-0.01–0.17)	0.293
Gunhan 2022		98	1.30 (0.98)	247	0.99 (0.59)	0.31 (0.10–0.52)	0.0058
Ji 2017		213	0.54 (0.28)	188	0.41 (0.21)	0.13 (0.08–0.18)	<0.0001
Luo 2021	Clinical LH	207	0.53 (0.25)	46	0.49 (0.22)	0.04 (-0.03–0.11)	NA
	Subclinical LH	63	0.43 (0.23)	46	0.49 (0.22)	-0.06 (-0.15–0.03)	NA
	Both	270	0.51 (0.25)	46	0.49 (0.22)	0.02 (-0.05–0.09)	NA
Omar 2011		14	0.99 (0.52)	51	0.75 (0.21)	0.24 (-0.04–0.52)	-
Singha 2021		46	1.26 (0.93)*	45	0.89 (0.56)*	NA	<0.001
Thewjitcharoen 2020		149	0.60 (0.30)	251	0.60 (0.30)	0.00 (-0.06–0.06)	0.441

*Median (IQR). NA – not available.

Table 31. Results in individual studies – daily insulin dose >60 insulin units/day

Study	LH+, n/N (%)	LH-, n/N (%)	pOR (95% CI)	p
Al Jaber 2020	51/80 (64%)	47/122 (39%)	2.81 (1.57–5.03)	<0.0001
Lin 2022	13/83 (16%)	6/37 (16%)	0.96 (0.33–2.76)	NA

NA – not available.

Table 32. Results in individual studies – daily insulin dose >40 insulin units/day

Study	LH+, n/N (%)	LH-, n/N (%)	pOR (95% CI)	p
Arora 2021	166/290 (57%)	90/210 (43%)	1.78 (1.25–2.56)	<0.001
Lin 2022	39/83 (47%)	15/37 (41%)	1.30 (0.59–2.85)	NA
Saeed 2022	151/157 (96%)	166/203 (82%)	5.61 (2.30–13.66)	<0.001

NA – not available.

Table 33. Results in individual studies – daily insulin dose >0.7 insulin units/kg/day

Study	LH+, n/N (%)	LH-, n/N (%)	pOR (95% CI)	p
Al Hayek 2016	77/83 (93%)	67/91 (74%)	4.60 (1.77–11.92)	<0.05
Tsadik 2018	69/103 (67%)	36/73 (49%)	2.09 (1.13–3.86)	<0.05

Table 34. Results in individual studies – daily insulin dose adjusted to HbA1c (%)

Study	LH+		LH-		MD (95% CI)	p
	N	Mean (SD)	N	Mean (SD)		
Barola 2018	231	13.7 (3.1)	141	13.2 (2.8)	0.50 (-0.11–1.11)	0.097

Table 35. Results in individual studies – hyperglycemia

Study	LH+, n/N (%)	LH-, n/N (%)	pOR (95% CI)	p
Kamrul-Hasan 2020	63/78 (81%)	508/769 (66%)	2.16 (1.21–3.86)	0.008
Nawaz 2023	66/83 (80%)	181/280 (65%)	2.12 (1.18–3.82)	0.01

Table 36. Results in individual studies – CGM data

Endpoint	Study	LH+		LH-		MD (95% CI)	p
		N	Mean (SD)	N	Mean (SD)		
Mean glucose level, mg/dL	Gupta 2018	10	224.8 (76.19)	10	165.40 (49.56)	59.40 (3.07–115.73)	0.027
Coefficient of variation, %	Gupta 2018	10	33.9%	10	29.96%	NA	NA
CONGA, mg/dL	Gupta 2018	10	196.78 (67.60)	10	98.44 (35.58)	98.34 (50.99–145.69)	0.002

FPG, mg/dL	Gentile 2022	487	165.70 (22.40)	673	133.60 (18.70)	32.10 (29.66–34.54)	<0.05
FPG, mg/dL	Gunhan 2022	98	177.00 (73.00)	247	191.00 (9.30)	-14.00 (-28.50–0.50)	0.2
LAGE, mmol/L	Lin 2022	83	5.70 (1.74)	83	4.21 (1.29)	1.49 (1.02–1.96)	NA
MAGE, mg/dL	Gupta 2018	10	216.3 (62.97)	10	173.7 (63.52)	42.60 (-12.84–98.04)	<0.0001
MBG, mmol/L	Lin 2022	83	10.13 (0.92)	83	9.43 (1.16)	0.70 (0.38–1.02)	NA
PPBG, mmol/L	Lin 2022	83	3.44 (1.24)	83	1.99 (0.64)	1.45 (1.15–1.75)	NA
SDBG, mmol/L	Lin 2022	83	1.96 (0.58)	83	1.39 (0.39)	0.57 (0.42–0.72)	NA

CONGA – continuous overlapping net glycemic control, FPG – fasting plasma glucose, LAGE – largest amplitude of glycemic excursions, MAGE – mean amplitude of glycemic excursions, MBG – mean blood glucose, PPBG – postprandial blood glucose, SDBG -standard deviation of blood glucose

Supplementary figures

Figure 1. PRISMA flow diagram

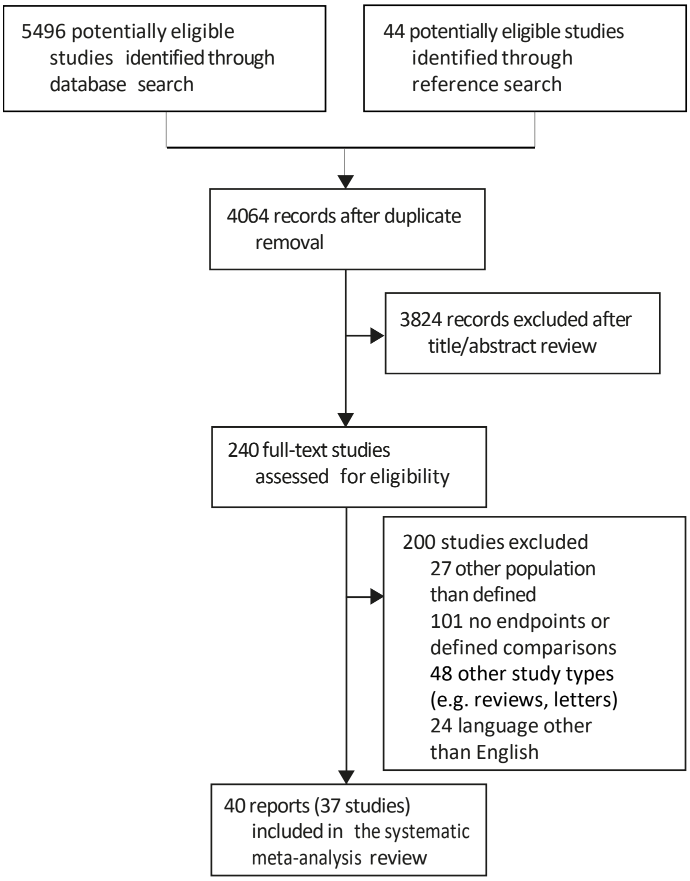

Figure 2. Forest plot for symptomatic hypoglycemia

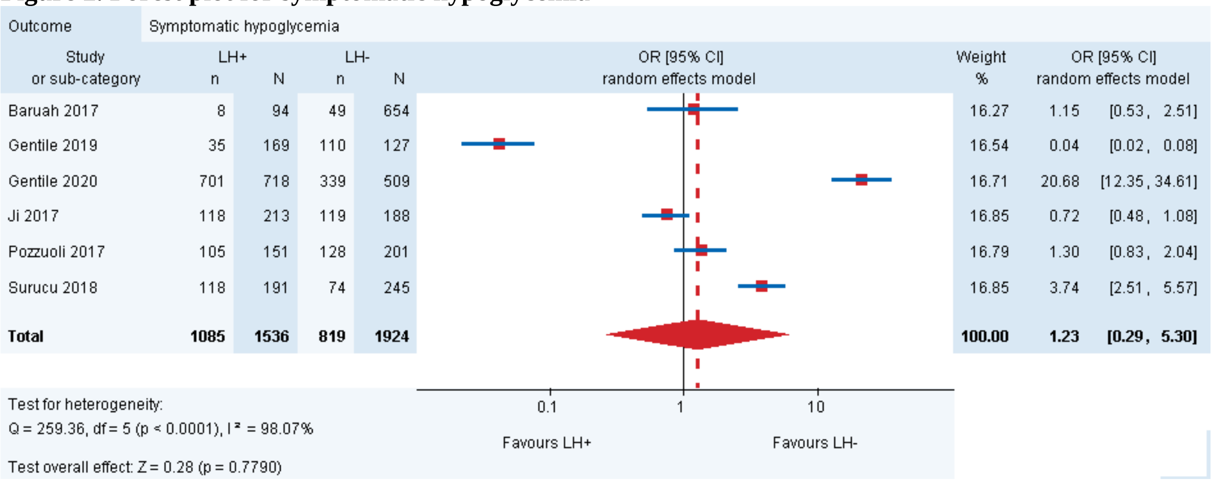

Figure 3. Forest plot for severe hypoglycemia

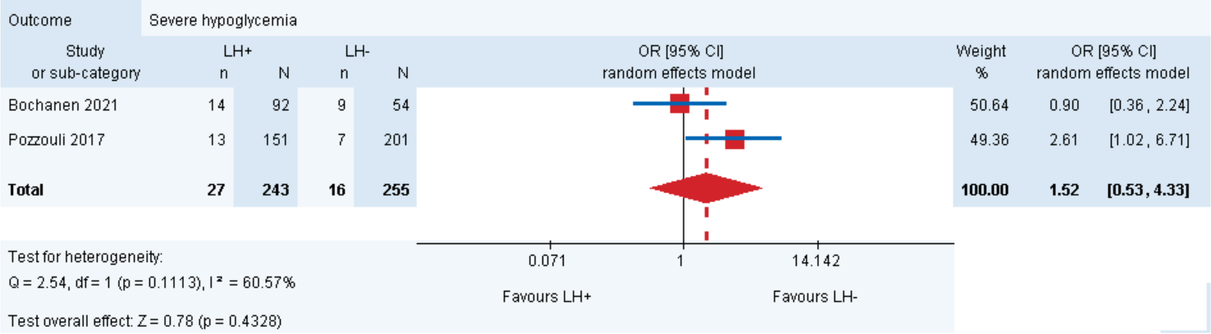

Figure 4. Forest plot for episodes of hypoglycemia

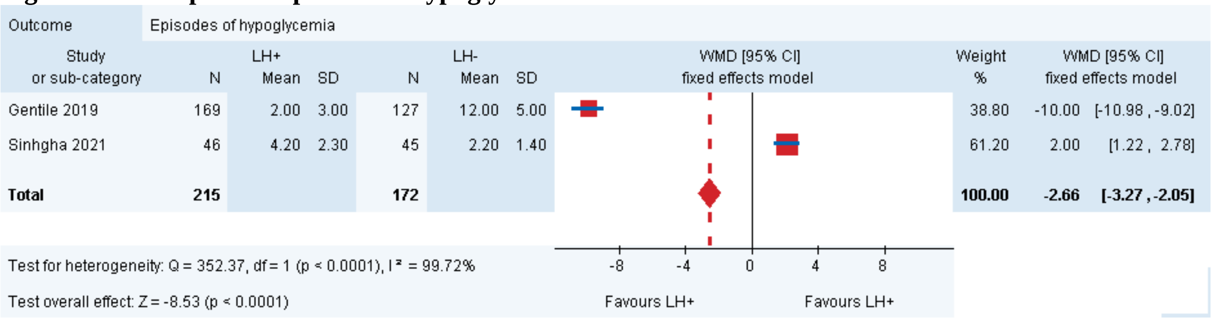

Figure 5. Forest plot for glycemic variability (mg/dl)

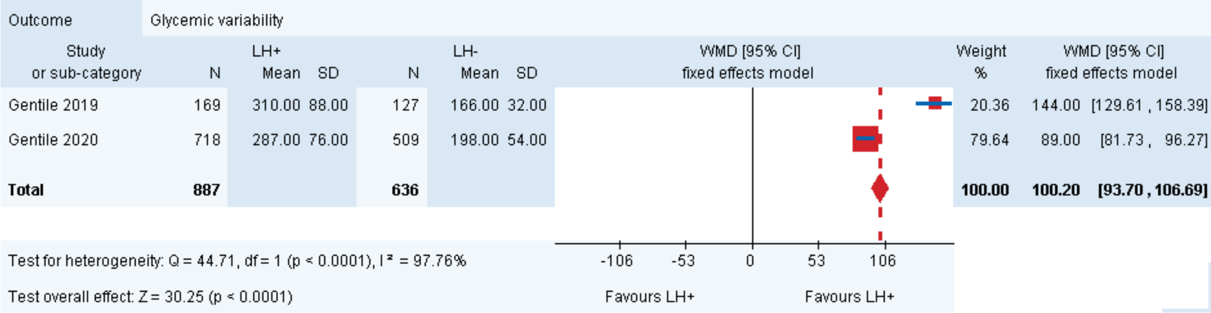

Figure 6. Forest plot for daily insulin dose adjusted to weight (insulin units/kg)

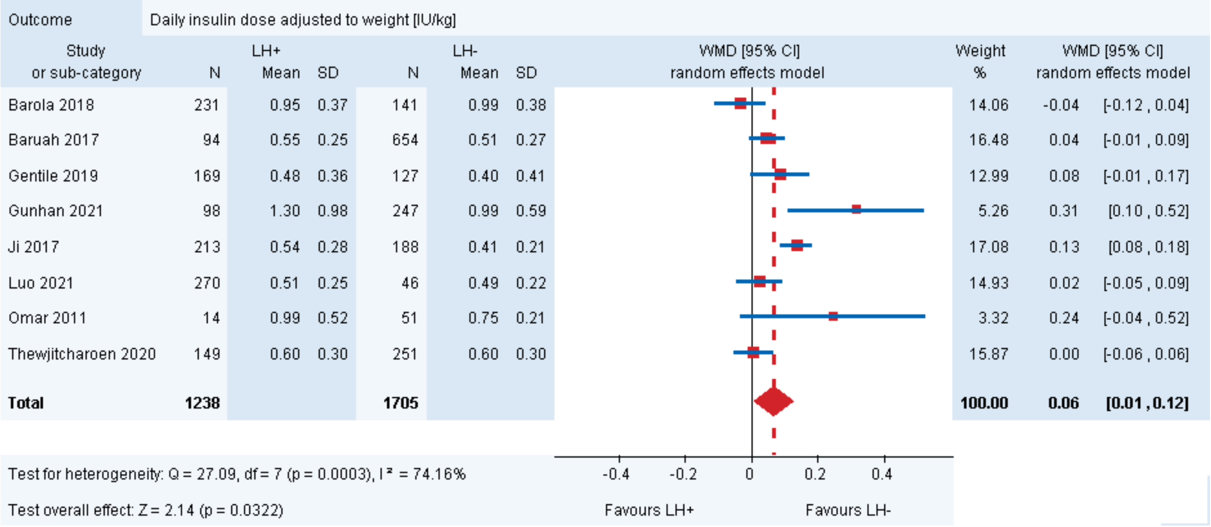

Outcome: Daily insulin dose adjusted to weight [IU/kg]

Study or sub-category	LH+ N	LH+ Mean	LH+ SD	LH- N	LH- Mean	LH- SD	Weight %	WMD [95% CI] random effects model
Barola 2018	231	0.95	0.37	141	0.99	0.38	14.06	-0.04 [-0.12 , 0.04]
Baruah 2017	94	0.55	0.25	654	0.51	0.27	16.48	0.04 [-0.01 , 0.09]
Gentile 2019	169	0.48	0.36	127	0.40	0.41	12.99	0.08 [-0.01 , 0.17]
Gunhan 2021	98	1.30	0.98	247	0.99	0.59	5.26	0.31 [0.10 , 0.52]
Ji 2017	213	0.54	0.28	188	0.41	0.21	17.08	0.13 [0.08 , 0.18]
Luo 2021	270	0.51	0.25	46	0.49	0.22	14.93	0.02 [-0.05 , 0.09]
Omar 2011	14	0.99	0.52	51	0.75	0.21	3.32	0.24 [-0.04 , 0.52]
Thewjitcharoen 2020	149	0.60	0.30	251	0.60	0.30	15.87	0.00 [-0.06 , 0.06]
**Total**	**1238**			**1705**			**100.00**	**0.06 [0.01 , 0.12]**

Test for heterogeneity: Q = 27.09, df = 7 (p = 0.0003), I² = 74.16%

Test overall effect: Z = 2.14 (p = 0.0322)

Figure 7. Forest plot for daily insulin dose >60 IU/day

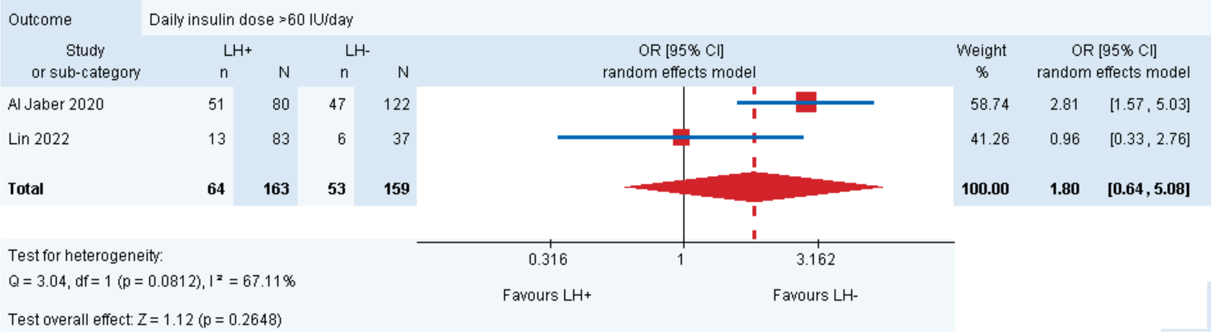

Outcome: Daily insulin dose >60 IU/day

Study or sub-category	LH+ n	LH+ N	LH- n	LH- N	Weight %	OR [95% CI] random effects model
Al Jaber 2020	51	80	47	122	58.74	2.81 [1.57 , 5.03]
Lin 2022	13	83	6	37	41.26	0.96 [0.33 , 2.76]
**Total**	**64**	**163**	**53**	**159**	**100.00**	**1.80 [0.64 , 5.08]**

Test for heterogeneity: Q = 3.04, df = 1 (p = 0.0812), I² = 67.11%

Test overall effect: Z = 1.12 (p = 0.2648)

Figure 8. Forest plot for daily insulin dose >40 IU/day

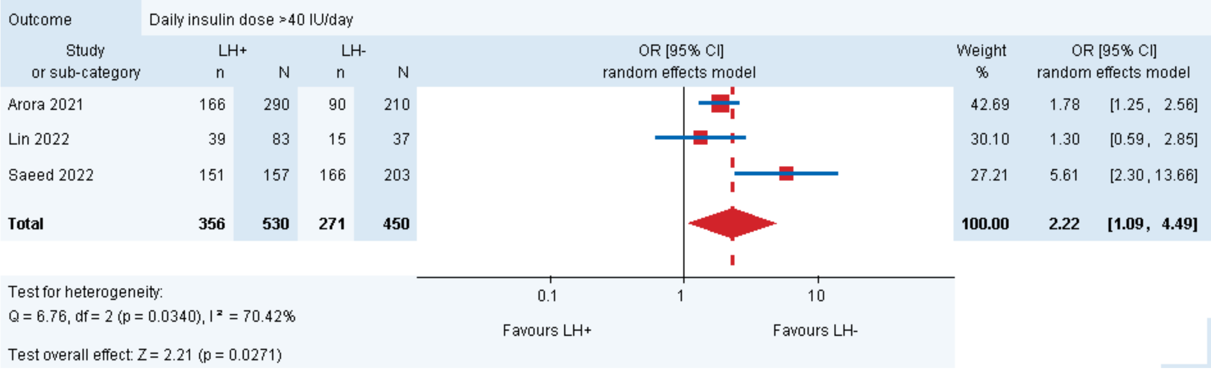

Outcome: Daily insulin dose >40 IU/day

Study or sub-category	LH+ n	LH+ N	LH- n	LH- N	Weight %	OR [95% CI] random effects model
Arora 2021	166	290	90	210	42.69	1.78 [1.25, 2.56]
Lin 2022	39	83	15	37	30.10	1.30 [0.59, 2.85]
Saeed 2022	151	157	166	203	27.21	5.61 [2.30, 13.66]
**Total**	**356**	**530**	**271**	**450**	**100.00**	**2.22 [1.09, 4.49]**

Test for heterogeneity: Q = 6.76, df = 2 (p = 0.0340), I² = 70.42%

Test overall effect: Z = 2.21 (p = 0.0271)

Figure 9. Forest plot for daily insulin dose >0.7 IU/kg/day

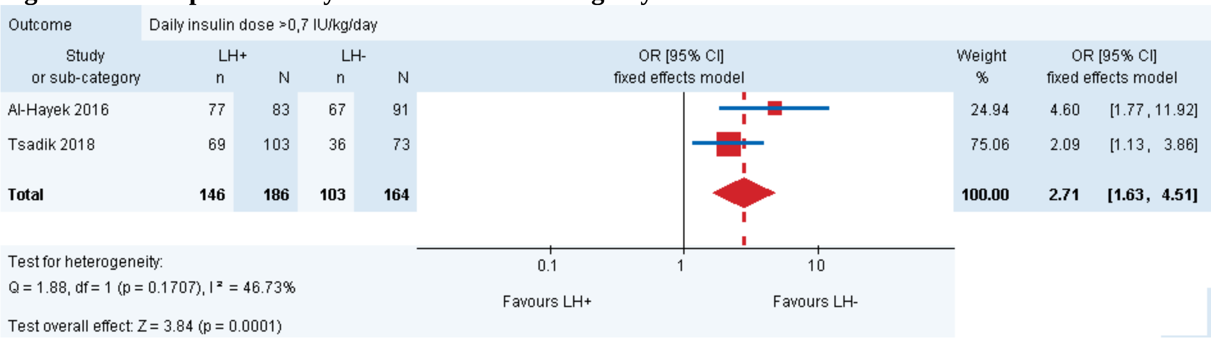

Outcome: Daily insulin dose >0,7 IU/kg/day

Study or sub-category	LH+ n	LH+ N	LH- n	LH- N	Weight %	OR [95% CI] fixed effects model
Al-Hayek 2016	77	83	67	91	24.94	4.60 [1.77, 11.92]
Tsadik 2018	69	103	36	73	75.06	2.09 [1.13, 3.86]
**Total**	**146**	**186**	**103**	**164**	**100.00**	**2.71 [1.63, 4.51]**

Test for heterogeneity: Q = 1.88, df = 1 (p = 0.1707), I² = 46.73%

Test overall effect: Z = 3.84 (p = 0.0001)

Figure 10. Forest plot for hyperglycemia

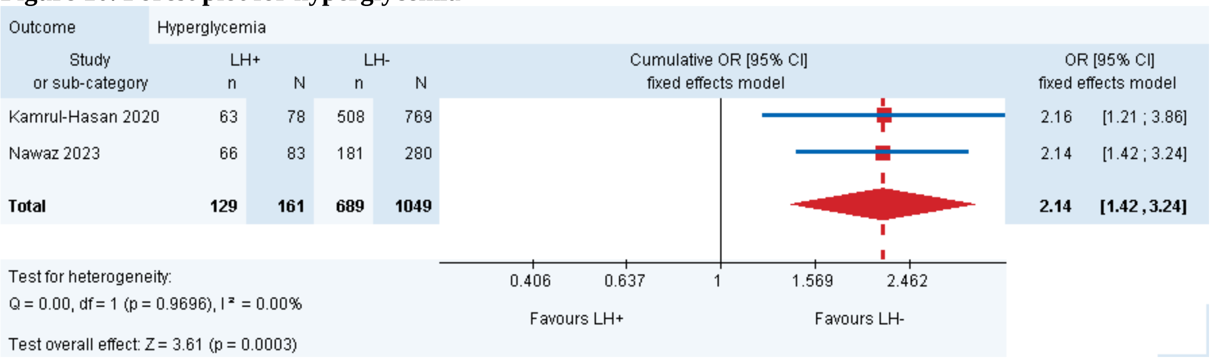

Figure 11. Forest plot for fasting plasma glucose (mg/dl)

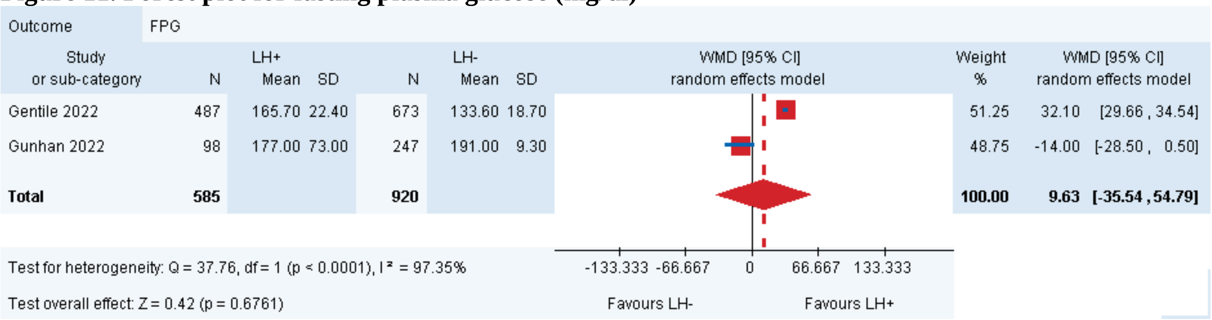

Figure 12. Subgroup analysis for unexplained hypoglycemia – types of diabetes

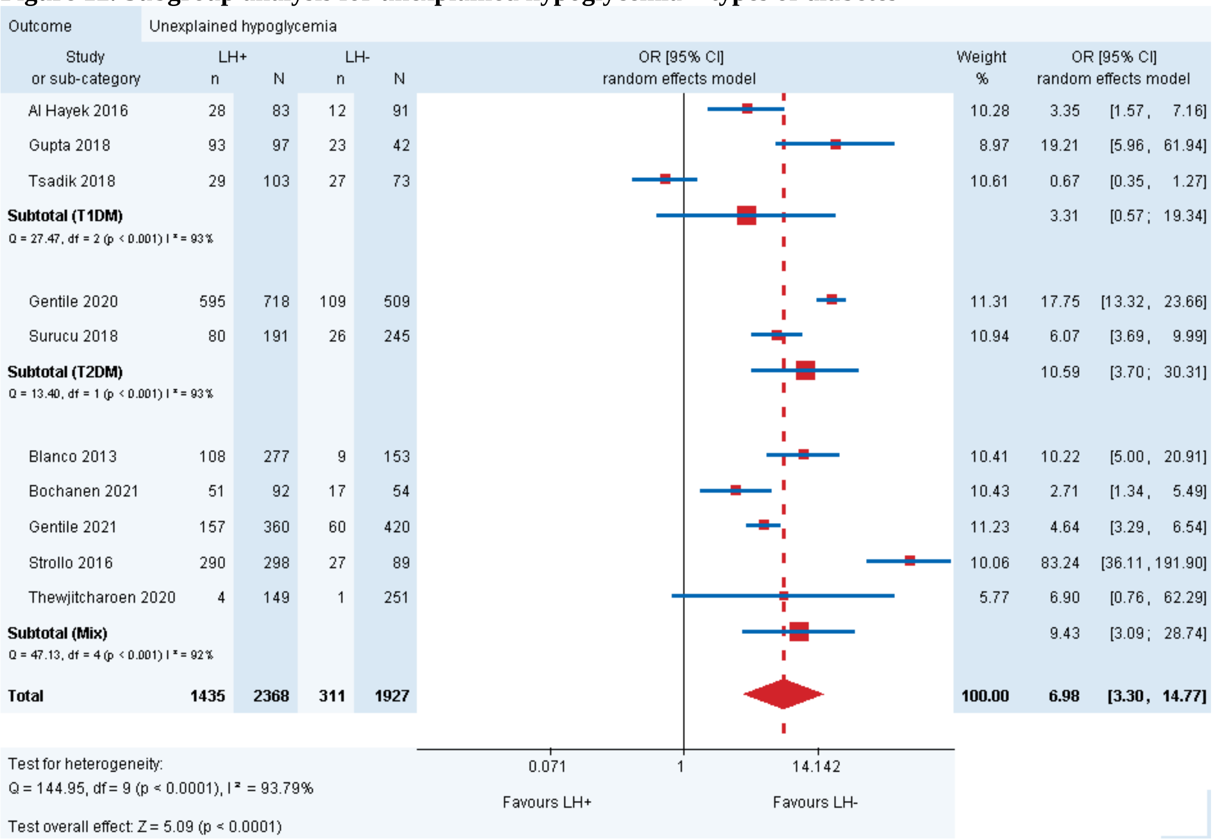

P-value for interaction: 0.523

Figure 13. Subgroup analysis for unexplained hypoglycemia –% of patients with T1DM

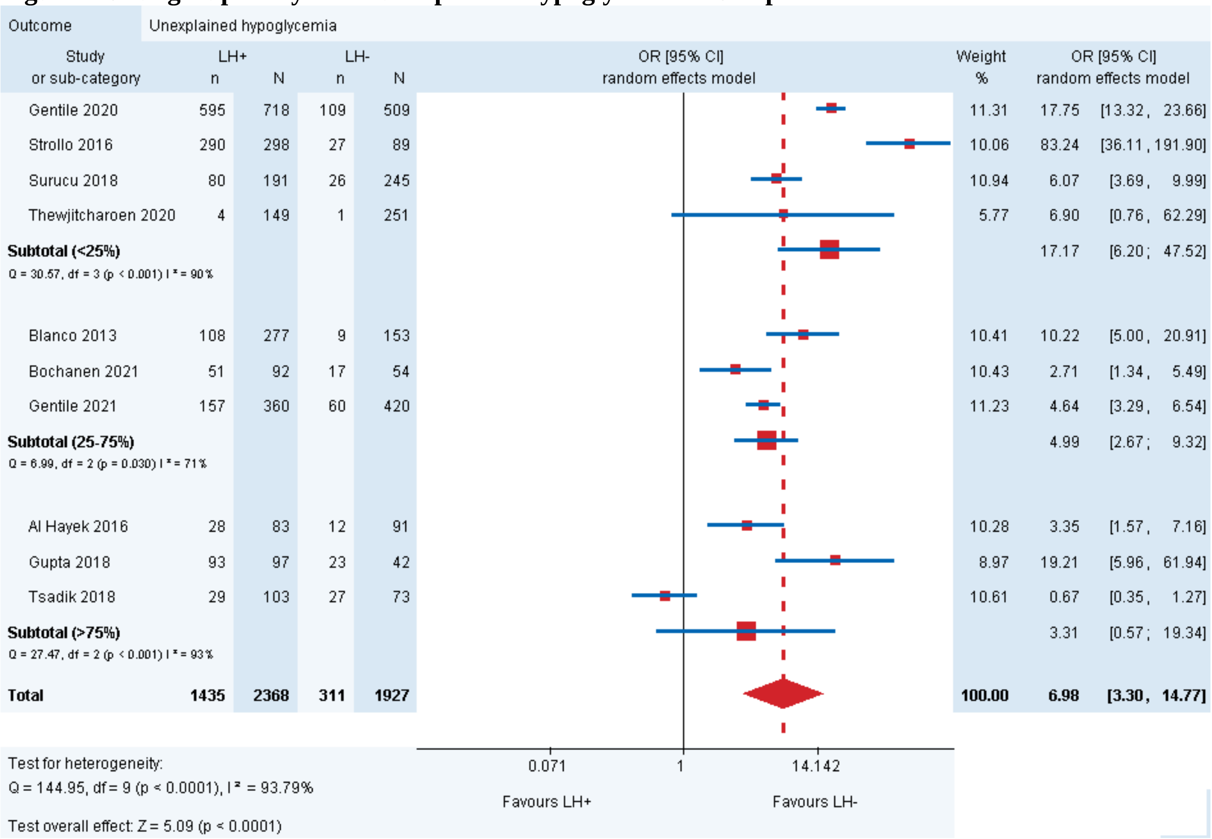

P-value for interaction: 0.093

Figure 14. Subgroup analysis for unexplained hypoglycemia – geographical region (general)

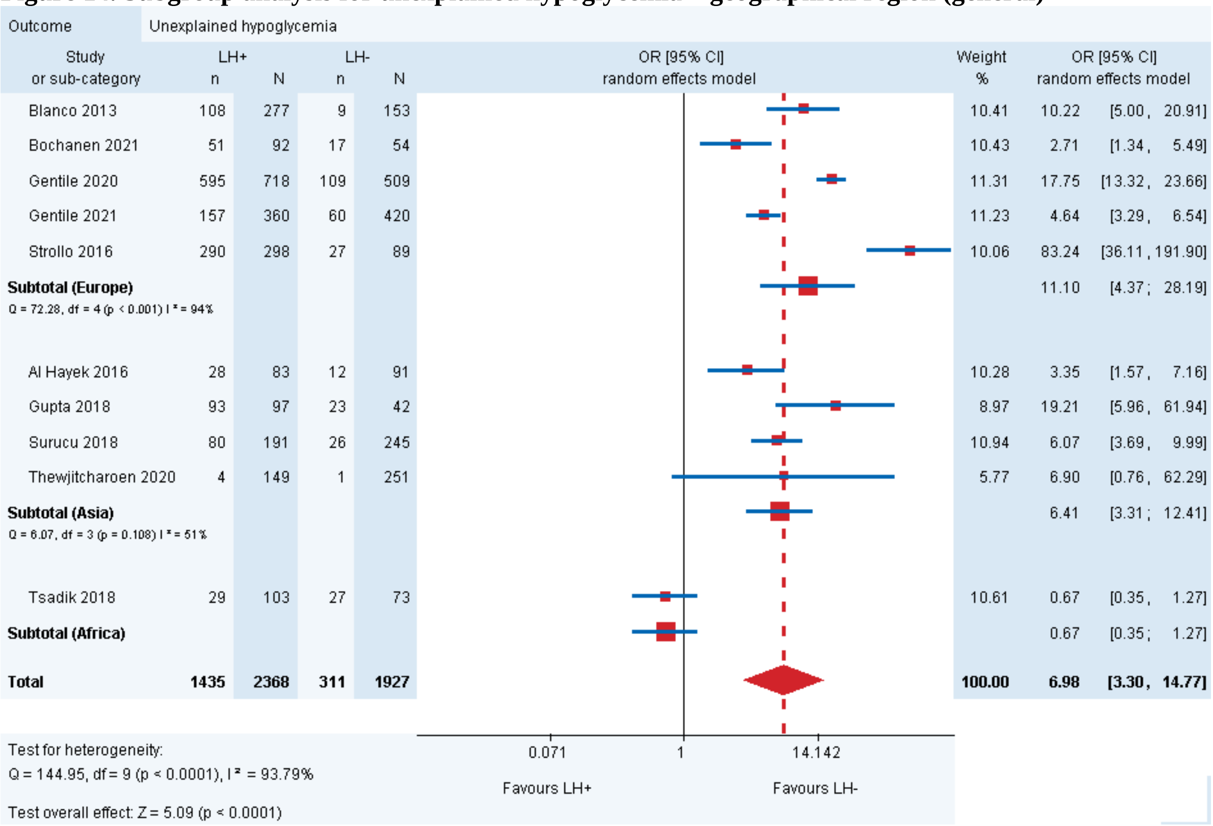

P-value for interaction: <0.0001

Figure 15. Subgroup analysis for unexplained hypoglycemia – geographical region (including Western vs Eastern Asia)

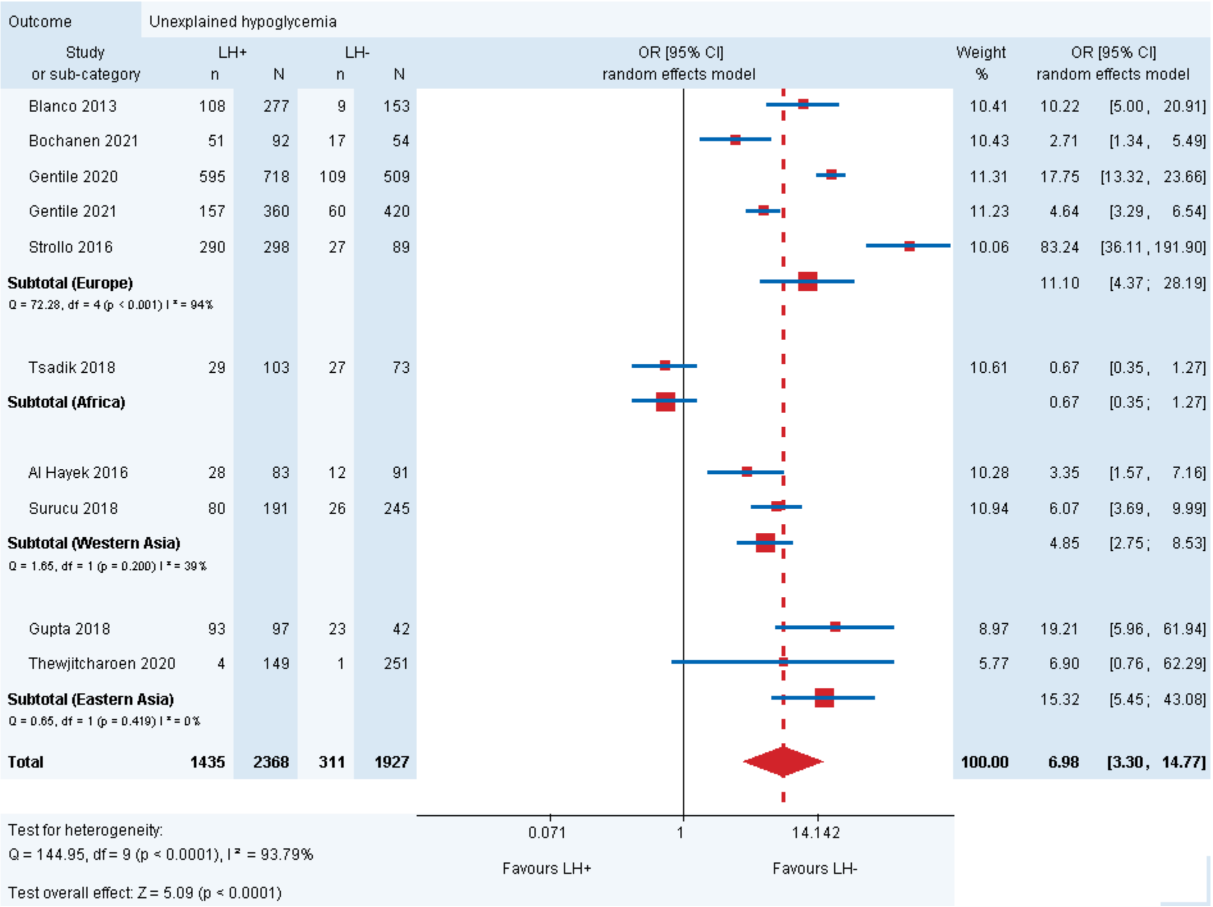

P-value for interaction: <0.0001

Figure 16. Subgroup analysis for unexplained hypoglycemia – duration of diabetes in years

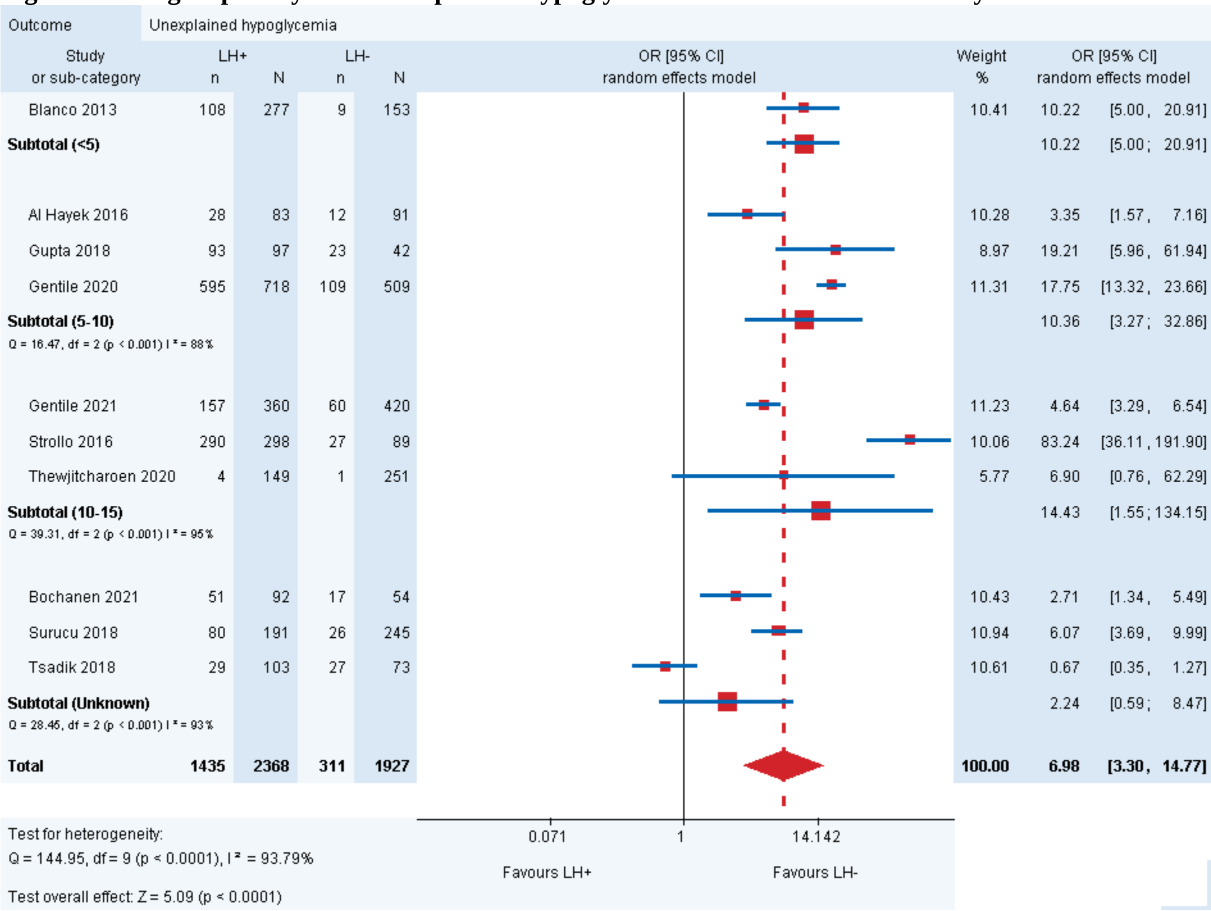

P-value for interaction: 0.219

Figure 17. Subgroup analysis for unexplained hypoglycemia – a type of lipohypertrophy assessment

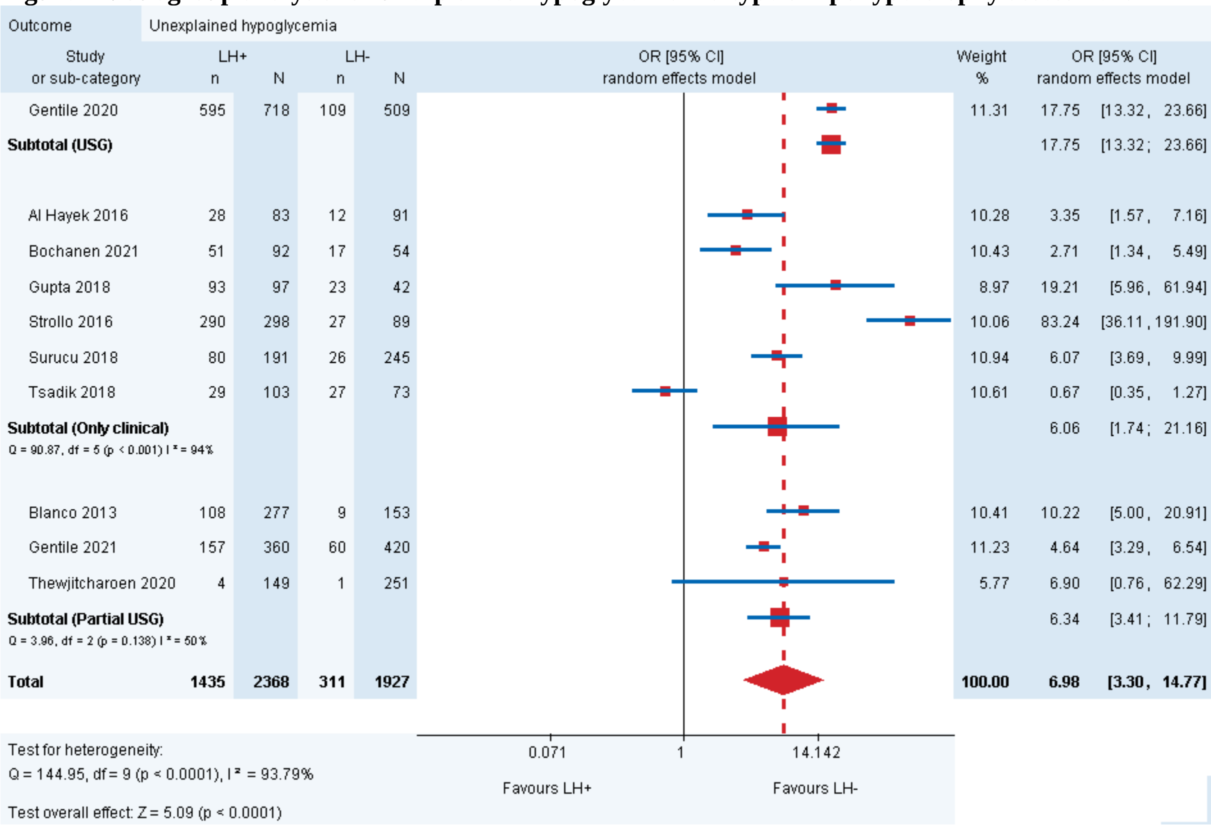

P-value for interaction: 0.005

Figure 18. Subgroup analysis for HbA1c (%) – types of diabetes

Outcome	HbA1c [%]								
Study or sub-category	LH+ N	LH+ Mean	LH+ SD	LH- N	LH- Mean	LH- SD	Weight %	WMD [95% CI] random effects model	
Barola 2018	231	10.00	2.70	141	9.20	2.40	4.03	0.80	[0.27 , 1.33]
Gupta 2018	97	9.62	2.13	42	9.84	2.66	3.32	-0.22	[-1.13 , 0.69]
Omar 2011	62	8.50	2.00	51	8.50	2.30	3.52	0.00	[-0.80 , 0.80]
Singha 2021	46	9.00	2.60	45	7.70	1.10	3.50	1.30	[0.48 , 2.12]
**Subtotal (T1DM)**								0.51	[-0.13 ; 1.14]
Q = 8.62, df = 3 (p = 0.035) I ² = 65 %									
Al Jaber 2020	80	9.53	1.60	122	9.14	1.70	4.13	0.39	[-0.07 , 0.85]
Baruah 2017	94	9.34	2.28	654	9.08	2.10	4.09	0.26	[-0.23 , 0.75]
Gentile 2020	718	9.60	1.10	509	7.50	1.10	4.48	2.10	[1.98 , 2.22]
Gentile 2022	487	8.60	1.60	673	7.30	1.00	4.46	1.30	[1.14 , 1.46]
Gunhan 2022	98	8.50	1.70	247	9.30	7.80	3.09	-0.80	[-1.83 , 0.23]
Ji 2014	134	7.87	1.53	246	8.17	4.41	3.89	-0.30	[-0.91 , 0.31]
Pahuja 2019	65	8.30	x	31	7.90	x	3.79	0.40	[-0.26 , 1.06]
**Subtotal (T2DM)**								0.55	[-0.12 ; 1.22]
Q = 201.01, df = 6 (p < 0.001) I ² = 97 %									
Arora 2021	290	10.53	1.73	210	8.95	1.53	4.36	1.58	[1.29 , 1.86]
Bochanen 2021	92	7.50	1.10	54	7.40	1.20	4.23	0.10	[-0.29 , 0.49]
Frid 2016	2205	8.85	2.70	4795	8.30	1.90	4.48	0.55	[0.43 , 0.67]
Gentile 2019	169	9.20	1.60	127	8.10	2.30	4.12	1.10	[0.63 , 1.57]
Gentile 2021	360	8.00	1.20	420	7.70	1.30	4.45	0.30	[0.12 , 0.48]
Hajheydari 2011	35	9.50	2.20	185	8.70	1.90	3.57	0.80	[0.02 , 1.58]
Hauner 1996	66	8.90	1.80	213	8.80	1.70	4.09	0.10	[-0.39 , 0.59]
Ji 2017	213	8.20	1.80	188	7.70	1.50	4.32	0.50	[0.18 , 0.82]
Kamrul-Hasan 2020	78	10.00	2.20	769	9.50	2.00	4.06	0.50	[-0.01 , 1.01]
Kumar 2021	79	9.12	2.53	8	8.68	1.39	2.93	0.44	[-0.67 , 1.55]
Luo 2021	270	8.63	1.76	46	7.42	1.73	4.00	1.21	[0.67 , 1.75]
Pozzouli 2018	151	7.70	1.40	202	7.70	1.30	4.36	0.00	[-0.29 , 0.29]
Strollo 2016	298	8.30	1.20	89	7.50	1.10	4.38	0.80	[0.53 , 1.07]
Thewjitcharoen 2020	149	7.80	1.50	251	7.90	1.70	4.32	-0.10	[-0.42 , 0.22]
**Subtotal (Mix)**								0.55	[0.31 ; 0.80]
Q = 109.65, df = 13 (p < 0.001) I ² = 88 %									
**Total**							**100.00**	**0.55**	**[0.23 , 0.87]**

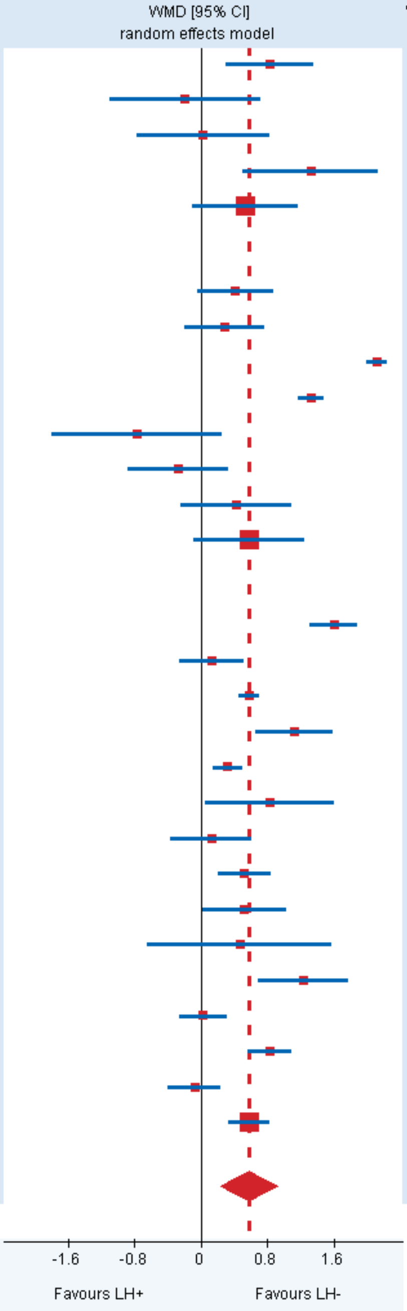

Test for heterogeneity: Q = 637.11, df = 24 (p < 0.0001), I ² = 96.23%

Test overall effect: Z = 3.35 (p = 0.0008)

P-value for interaction: 0.993

Figure 19. Subgroup analysis for HbA1c (%) – % of patients with T1DM

Outcome	HbA1c [%]								
Study or sub-category	LH+ N	LH+ Mean	LH+ SD	LH- N	LH- Mean	LH- SD	Weight %	WMD [95% CI] random effects model	
Al Jaber 2020	80	9.53	1.60	122	9.14	1.70	4.13	0.39	[-0.07 , 0.85]
Arora 2021	290	10.53	1.73	210	8.95	1.53	4.36	1.58	[1.29 , 1.86]
Baruah 2017	94	9.34	2.28	654	9.08	2.10	4.09	0.26	[-0.23 , 0.75]
Gentile 2019	169	9.20	1.60	127	8.10	2.30	4.12	1.10	[0.63 , 1.57]
Gentile 2020	718	9.60	1.10	509	7.50	1.10	4.48	2.10	[1.98 , 2.22]
Gentile 2022	487	8.60	1.60	673	7.30	1.00	4.46	1.30	[1.14 , 1.46]
Gunhan 2022	98	8.50	1.70	247	9.30	7.80	3.09	-0.80	[-1.83 , 0.23]
Ji 2014	134	7.87	1.53	246	8.17	4.41	3.89	-0.30	[-0.91 , 0.31]
Ji 2017	213	8.20	1.80	188	7.70	1.50	4.32	0.50	[0.18 , 0.82]
Kamrul-Hasan 2020	78	10.00	2.20	769	9.50	2.00	4.06	0.50	[-0.01 , 1.01]
Kumar 2021	79	9.12	2.53	8	8.68	1.39	2.93	0.44	[-0.67 , 1.55]
Luo 2021	270	8.63	1.76	46	7.42	1.73	4.00	1.21	[0.67 , 1.75]
Pahuja 2019	65	8.30	x	31	7.90	x	3.79	0.40	[-0.26 , 1.06]
Pozzouli 2018	151	7.70	1.40	202	7.70	1.30	4.36	0.00	[-0.29 , 0.29]
Strollo 2016	298	8.30	1.20	89	7.50	1.10	4.38	0.80	[0.53 , 1.07]
Thewjitcharoen 2020	149	7.80	1.50	251	7.90	1.70	4.32	-0.10	[-0.42 , 0.22]
**Subtotal (<25%)**								0.62	[0.18 ; 1.06]
Q = 442.82, df = 15 (p < 0.001) I ² = 97 %									
Bochanen 2021	92	7.50	1.10	54	7.40	1.20	4.23	0.10	[-0.29 , 0.49]
Frid 2016	2205	8.85	2.70	4795	8.30	1.90	4.48	0.55	[0.43 , 0.67]
Gentile 2021	360	8.00	1.20	420	7.70	1.30	4.45	0.30	[0.12 , 0.48]
Hajheydari 2011	35	9.50	2.20	185	8.70	1.90	3.57	0.80	[0.02 , 1.58]
**Subtotal (25-75%)**								0.40	[0.17 ; 0.62]
Q = 9.12, df = 3 (p = 0.028) I ² = 67 %									
Barola 2018	231	10.00	2.70	141	9.20	2.40	4.03	0.80	[0.27 , 1.33]
Gupta 2018	97	9.62	2.13	42	9.84	2.66	3.32	-0.22	[-1.13 , 0.69]
Hauner 1996	66	8.90	1.80	213	8.80	1.70	4.09	0.10	[-0.39 , 0.59]
Omar 2011	62	8.50	2.00	51	8.50	2.30	3.52	0.00	[-0.80 , 0.80]
Singha 2021	46	9.00	2.60	45	7.70	1.10	3.50	1.30	[0.48 , 2.12]
**Subtotal (>75%)**								0.41	[-0.09 ; 0.91]
Q = 11.03, df = 4 (p = 0.026) I ² = 64%									
**Total**							**100.00**	**0.55**	**[0.23 , 0.87]**

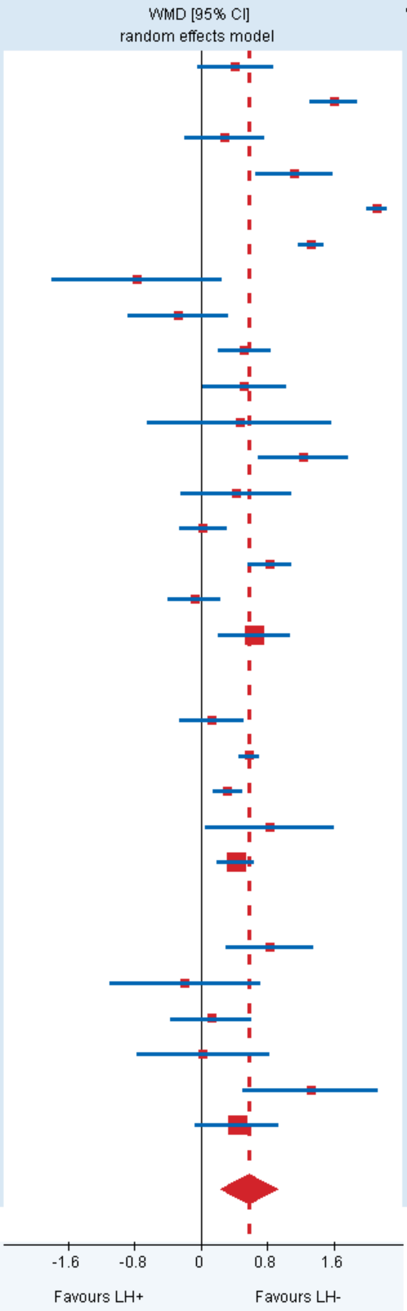

Test for heterogeneity: Q = 637.11, df = 24 (p < 0.0001), I ² = 96.23%

Test overall effect: Z = 3.35 (p = 0.0008)

P-value for interaction: 0.676

Figure 20. Subgroup analysis for HbA1c (%) – geographical region (general)

Outcome: HbA1c [%]

Study or sub-category	LH+ N	LH+ Mean	LH+ SD	LH- N	LH- Mean	LH- SD	Weight %	WMD [95% CI] random effects model
Al Jaber 2020	80	9.53	1.60	122	9.14	1.70	4.13	0.39 [-0.07 , 0.85]
Arora 2021	290	10.53	1.73	210	8.95	1.53	4.36	1.58 [1.29 , 1.86]
Barola 2018	231	10.00	2.70	141	9.20	2.40	4.03	0.80 [0.27 , 1.33]
Baruah 2017	94	9.34	2.28	654	9.08	2.10	4.09	0.26 [-0.23 , 0.75]
Gupta 2018	97	9.62	2.13	42	9.84	2.66	3.32	-0.22 [-1.13 , 0.69]
Gunhan 2022	98	8.50	1.70	247	9.30	7.80	3.09	-0.80 [-1.83 , 0.23]
Hajheydari 2011	35	9.50	2.20	185	8.70	1.90	3.57	0.80 [0.02 , 1.58]
Ji 2014	134	7.87	1.53	246	8.17	4.41	3.89	-0.30 [-0.91 , 0.31]
Ji 2017	213	8.20	1.80	188	7.70	1.50	4.32	0.50 [0.18 , 0.82]
Kamrul-Hasan 2020	78	10.00	2.20	769	9.50	2.00	4.06	0.50 [-0.01 , 1.01]
Kumar 2021	79	9.12	2.53	8	8.68	1.39	2.93	0.44 [-0.67 , 1.55]
Luo 2021	270	8.63	1.76	46	7.42	1.73	4.00	1.21 [0.67 , 1.75]
Pahuja 2019	65	8.30	x	31	7.90	x	3.79	0.40 [-0.26 , 1.06]
Singha 2021	46	9.00	2.60	45	7.70	1.10	3.50	1.30 [0.48 , 2.12]
Thewjitcharoen 2020	149	7.80	1.50	251	7.90	1.70	4.32	-0.10 [-0.42 , 0.22]
**Subtotal (Asia)**								0.48 [0.13 ; 0.83]
Q = 93.64, df = 14 (p < 0.001) I² = 85%								
Bochanen 2021	92	7.50	1.10	54	7.40	1.20	4.23	0.10 [-0.29 , 0.49]
Gentile 2019	169	9.20	1.60	127	8.10	2.30	4.12	1.10 [0.63 , 1.57]
Gentile 2020	718	9.60	1.10	509	7.50	1.10	4.48	2.10 [1.98 , 2.22]
Gentile 2021	360	8.00	1.20	420	7.70	1.30	4.45	0.30 [0.12 , 0.48]
Gentile 2022	487	8.60	1.60	673	7.30	1.00	4.46	1.30 [1.14 , 1.46]
Hauner 1996	66	8.90	1.80	213	8.80	1.70	4.09	0.10 [-0.39 , 0.59]
Pozzouli 2018	151	7.70	1.40	202	7.70	1.30	4.36	0.00 [-0.29 , 0.29]
Strollo 2016	298	8.30	1.20	89	7.50	1.10	4.38	0.80 [0.53 , 1.07]
**Subtotal (Europe)**								0.73 [0.10 ; 1.36]
Q = 427.83, df = 7 (p < 0.001) I² = 98%								
Omar 2011	62	8.50	2.00	51	8.50	2.30	3.52	0.00 [-0.80 , 0.80]
**Subtotal (Africa)**								0.00 [-0.80 ; 0.80]
Frid 2016	2205	8.85	2.70	4795	8.30	1.90	4.48	0.55 [0.43 , 0.67]
**Subtotal (Worldwide)**								0.55 [0.43 ; 0.67]
**Total**							**100.00**	**0.55 [0.23 , 0.87]**

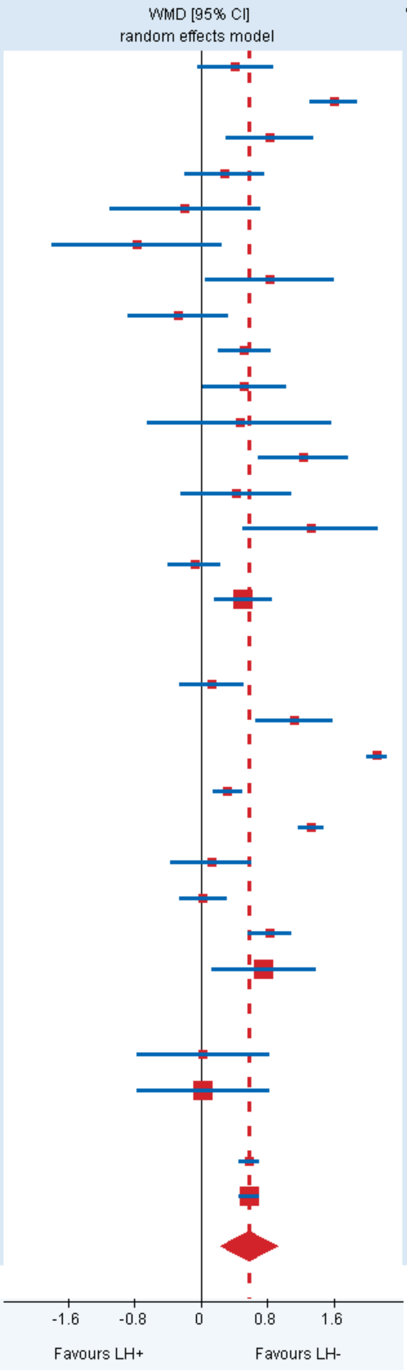

Test for heterogeneity: Q = 637.11, df = 24 (p < 0.0001), I² = 96.23%

Test overall effect: Z = 3.35 (p = 0.0008)

P-value for interaction: 0.524

Figure 21. Subgroup analysis for HbA1c (%) – geographical region (including Western vs Eastern Asia)

Outcome: HbA1c [%]

Study or sub-category	LH+ N	LH+ Mean	LH+ SD	LH- N	LH- Mean	LH- SD	Weight %	WMD [95% CI] random effects model	
Bochanen 2021	92	7.50	1.10	54	7.40	1.20	4.23	0.10	[-0.29 , 0.49]
Gentile 2019	169	9.20	1.60	127	8.10	2.30	4.12	1.10	[0.63 , 1.57]
Gentile 2020	718	9.60	1.10	509	7.50	1.10	4.48	2.10	[1.98 , 2.22]
Gentile 2021	360	8.00	1.20	420	7.70	1.30	4.45	0.30	[0.12 , 0.48]
Gentile 2022	487	8.60	1.60	673	7.30	1.00	4.46	1.30	[1.14 , 1.46]
Hauner 1996	66	8.90	1.80	213	8.80	1.70	4.09	0.10	[-0.39 , 0.59]
Pozzouli 2018	151	7.70	1.40	202	7.70	1.30	4.36	0.00	[-0.29 , 0.29]
Strollo 2016	298	8.30	1.20	89	7.50	1.10	4.38	0.80	[0.53 , 1.07]
**Subtotal (Europe)**								0.73	[0.10 ; 1.36]
Q = 427.83, df = 7 (p < 0.001) I ² = 98 %									
Omar 2011	62	8.50	2.00	51	8.50	2.30	3.52	0.00	[-0.80 , 0.80]
**Subtotal (Africa)**								0.00	[-0.80 ; 0.80]
Frid 2016	2205	8.85	2.70	4795	8.30	1.90	4.48	0.55	[0.43 , 0.67]
**Subtotal (Worldwide)**								0.55	[0.43 ; 0.67]
Arora 2021	290	10.53	1.73	210	8.95	1.53	4.36	1.58	[1.29 , 1.86]
Barola 2018	231	10.00	2.70	141	9.20	2.40	4.03	0.80	[0.27 , 1.33]
Baruah 2017	94	9.34	2.28	654	9.08	2.10	4.09	0.26	[-0.23 , 0.75]
Gupta 2018	97	9.62	2.13	42	9.84	2.66	3.32	-0.22	[-1.13 , 0.69]
Ji 2014	134	7.87	1.53	246	8.17	4.41	3.89	-0.30	[-0.91 , 0.31]
Ji 2017	213	8.20	1.80	188	7.70	1.50	4.32	0.50	[0.18 , 0.82]
Kamrul-Hasan 2020	78	10.00	2.20	769	9.50	2.00	4.06	0.50	[-0.01 , 1.01]
Kumar 2021	79	9.12	2.53	8	8.68	1.39	2.93	0.44	[-0.67 , 1.55]
Luo 2021	270	8.63	1.76	46	7.42	1.73	4.00	1.21	[0.67 , 1.75]
Pahuja 2019	65	8.30	x	31	7.90	x	3.79	0.40	[-0.26 , 1.06]
Singha 2021	46	9.00	2.60	45	7.70	1.10	3.50	1.30	[0.48 , 2.12]
Thewjitcharoen 2020	149	7.80	1.50	251	7.90	1.70	4.32	-0.10	[-0.42 , 0.22]
**Subtotal (Eastern Asia)**								0.54	[0.14 ; 0.94]
Q = 85.19, df = 11 (p < 0.001) I ² = 87 %									
Al Jaber 2020	80	9.53	1.60	122	9.14	1.70	4.13	0.39	[-0.07 , 0.85]
Gunhan 2022	98	8.50	1.70	247	9.30	7.80	3.09	-0.80	[-1.83 , 0.23]
Hajheydari 2011	35	9.50	2.20	185	8.70	1.90	3.57	0.80	[0.02 , 1.58]
**Subtotal (Western Asia)**								0.22	[-0.52 ; 0.96]
Q = 6.09, df = 2 (p = 0.047) I ² = 67 %									
**Total**							**100.00**	**0.55**	**[0.23 , 0.87]**

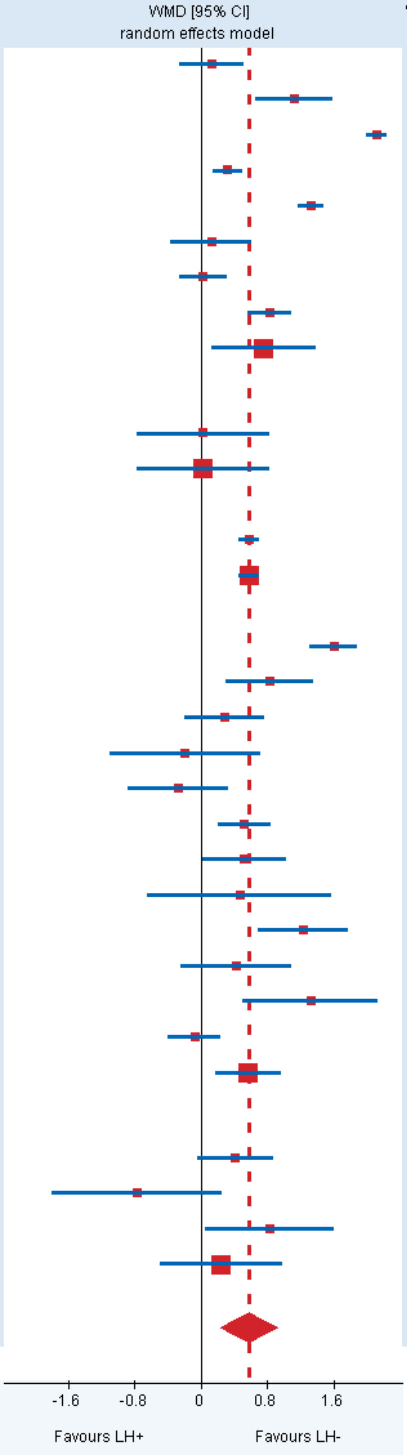

Test for heterogeneity: Q = 637.11, df = 24 (p < 0.0001), I ² = 96.23%

Test overall effect: Z = 3.35 (p = 0.0008)

P-value for interaction: 0.585

Figure 22. Subgroup analysis for HbA1c (%) – duration of diabetes in years

Outcome: HbA1c [%]

Study or sub-category	LH+ N	LH+ Mean	LH+ SD	LH- N	LH- Mean	LH- SD	Weight %	WMD [95% CI] random effects model
Arora 2021	290	10.53	1.73	210	8.95	1.53	4.36	1.58 [1.29 , 1.86]
Baruah 2017	94	9.34	2.28	654	9.08	2.10	4.09	0.26 [-0.23 , 0.75]
Gentile 2019	169	9.20	1.60	127	8.10	2.30	4.12	1.10 [0.63 , 1.57]
Ji 2014	134	7.87	1.53	246	8.17	4.41	3.89	-0.30 [-0.91 , 0.31]
Kamrul-Hasan 2020	78	10.00	2.20	769	9.50	2.00	4.06	0.50 [-0.01 , 1.01]
Omar 2011	62	8.50	2.00	51	8.50	2.30	3.52	0.00 [-0.80 , 0.80]
**Subtotal (<5)**								0.56 [-0.07 ; 1.19]
Q = 50.79, df = 5 (p < 0.001) I^2 = 90%								
Al Jaber 2020	80	9.53	1.60	122	9.14	1.70	4.13	0.39 [-0.07 , 0.85]
Frid 2016	2205	8.85	2.70	4795	8.30	1.90	4.48	0.55 [0.43 , 0.67]
Gentile 2020	718	9.60	1.10	509	7.50	1.10	4.48	2.10 [1.98 , 2.22]
Gentile 2022	487	8.60	1.60	673	7.30	1.00	4.46	1.30 [1.14 , 1.46]
Gupta 2018	97	9.62	2.13	42	9.84	2.66	3.32	-0.22 [-1.13 , 0.69]
Gunhan 2022	98	8.50	1.70	247	9.30	7.80	3.09	-0.80 [-1.83 , 0.23]
Hajheydari 2011	35	9.50	2.20	185	8.70	1.90	3.57	0.80 [0.02 , 1.58]
Ji 2017	213	8.20	1.80	188	7.70	1.50	4.32	0.50 [0.18 , 0.82]
Kumar 2021	79	9.12	2.53	8	8.68	1.39	2.93	0.44 [-0.67 , 1.55]
Luo 2021	270	8.63	1.76	46	7.42	1.73	4.00	1.21 [0.67 , 1.75]
Pahuja 2019	65	8.30	x	31	7.90	x	3.79	0.40 [-0.26 , 1.06]
Pozzouli 2018	151	7.70	1.40	202	7.70	1.30	4.36	0.00 [-0.29 , 0.29]
**Subtotal (5-10)**								0.61 [0.10 ; 1.11]
Q = 431.83, df = 11 (p < 0.001) I^2 = 97%								
Gentile 2021	360	8.00	1.20	420	7.70	1.30	4.45	0.30 [0.12 , 0.48]
Singha 2021	46	9.00	2.60	45	7.70	1.10	3.50	1.30 [0.48 , 2.12]
Strollo 2016	298	8.30	1.20	89	7.50	1.10	4.38	0.80 [0.53 , 1.07]
Thewjitcharoen 2020	149	7.80	1.50	251	7.90	1.70	4.32	-0.10 [-0.42 , 0.22]
**Subtotal (10-15)**								0.48 [0.05 ; 0.92]
Q = 23.90, df = 3 (p < 0.001) I^2 = 87%								
Barola 2018	231	10.00	2.70	141	9.20	2.40	4.03	0.80 [0.27 , 1.33]
Bochanen 2021	92	7.50	1.10	54	7.40	1.20	4.23	0.10 [-0.29 , 0.49]
Hauner 1996	66	8.90	1.80	213	8.80	1.70	4.09	0.10 [-0.39 , 0.59]
**Subtotal (Unknown)**								0.31 [-0.12 ; 0.74]
Q = 5.06, df = 2 (p = 0.079) I^2 = 61%								
**Total**							**100.00**	**0.55 [0.23 , 0.87]**

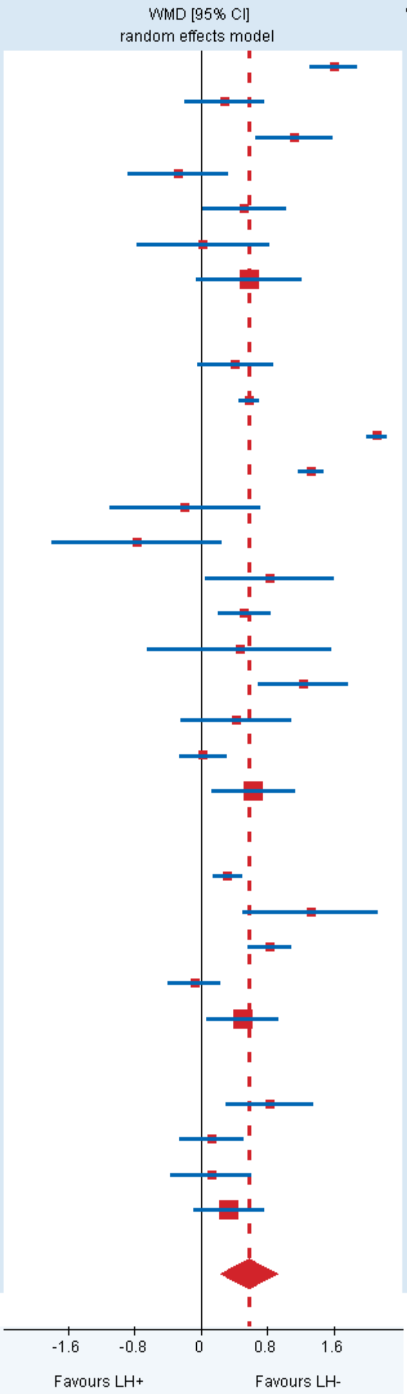

Test for heterogeneity: Q = 637.11, df = 24 (p < 0.0001), I^2 = 96.23%

Test overall effect: Z = 3.35 (p = 0.0008)

P-value for interaction: 0.822

Figure 23. Subgroup analysis for HbA1c (%) – a type of lipohypertrophy measurement

Outcome: HbA1c [%]

Study or sub-category	LH+ N	LH+ Mean	LH+ SD	LH- N	LH- Mean	LH- SD	Weight %	WMD [95% CI] random effects model	
Al Jaber 2020	80	9.53	1.60	122	9.14	1.70	4.13	0.39	[-0.07 , 0.85]
Barola 2018	231	10.00	2.70	141	9.20	2.40	4.03	0.80	[0.27 , 1.33]
Baruah 2017	94	9.34	2.28	654	9.08	2.10	4.09	0.26	[-0.23 , 0.75]
Bochanen 2021	92	7.50	1.10	54	7.40	1.20	4.23	0.10	[-0.29 , 0.49]
Frid 2016	2205	8.85	2.70	4795	8.30	1.90	4.48	0.55	[0.43 , 0.67]
Gentile 2019	169	9.20	1.60	127	8.10	2.30	4.12	1.10	[0.63 , 1.57]
Gupta 2018	97	9.62	2.13	42	9.84	2.66	3.32	-0.22	[-1.13 , 0.69]
Gunhan 2022	98	8.50	1.70	247	9.30	7.80	3.09	-0.80	[-1.83 , 0.23]
Hajheydari 2011	35	9.50	2.20	185	8.70	1.90	3.57	0.80	[0.02 , 1.58]
Hauner 1996	66	8.90	1.80	213	8.80	1.70	4.09	0.10	[-0.39 , 0.59]
Ji 2014	134	7.87	1.53	246	8.17	4.41	3.89	-0.30	[-0.91 , 0.31]
Ji 2017	213	8.20	1.80	188	7.70	1.50	4.32	0.50	[0.18 , 0.82]
Kamrul-Hasan 2020	78	10.00	2.20	769	9.50	2.00	4.06	0.50	[-0.01 , 1.01]
Omar 2011	62	8.50	2.00	51	8.50	2.30	3.52	0.00	[-0.80 , 0.80]
Pahuja 2019	65	8.30	x	31	7.90	x	3.79	0.40	[-0.26 , 1.06]
Pozzouli 2018	151	7.70	1.40	202	7.70	1.30	4.36	0.00	[-0.29 , 0.29]
Strollo 2016	298	8.30	1.20	89	7.50	1.10	4.38	0.80	[0.53 , 1.07]
**Subtotal (Only clinical)**								0.37	[0.19 ; 0.55]
Q = 49.04, df = 16 (p < 0.001) I² = 67%									
Gentile 2021	360	8.00	1.20	420	7.70	1.30	4.45	0.30	[0.12 , 0.48]
Thewjitcharoen 2020	149	7.80	1.50	251	7.90	1.70	4.32	-0.10	[-0.42 , 0.22]
**Subtotal (Partial USG)**								0.12	[-0.27 ; 0.51]
Q = 4.62, df = 1 (p = 0.032) I² = 78%									
Arora 2021	290	10.53	1.73	210	8.95	1.53	4.36	1.58	[1.29 , 1.86]
Gentile 2020	718	9.60	1.10	509	7.50	1.10	4.48	2.10	[1.98 , 2.22]
Gentile 2022	487	8.60	1.60	673	7.30	1.00	4.46	1.30	[1.14 , 1.46]
Kumar 2021	79	9.12	2.53	8	8.68	1.39	2.93	0.44	[-0.67 , 1.55]
Luo 2021	270	8.63	1.76	46	7.42	1.73	4.00	1.21	[0.67 , 1.75]
Singha 2021	46	9.00	2.60	45	7.70	1.10	3.50	1.30	[0.48 , 2.12]
**Subtotal (USG)**								1.43	[0.99 ; 1.88]
Q = 71.87, df = 5 (p < 0.001) I² = 93%									
**Total**							**100.00**	**0.55**	**[0.23 , 0.87]**

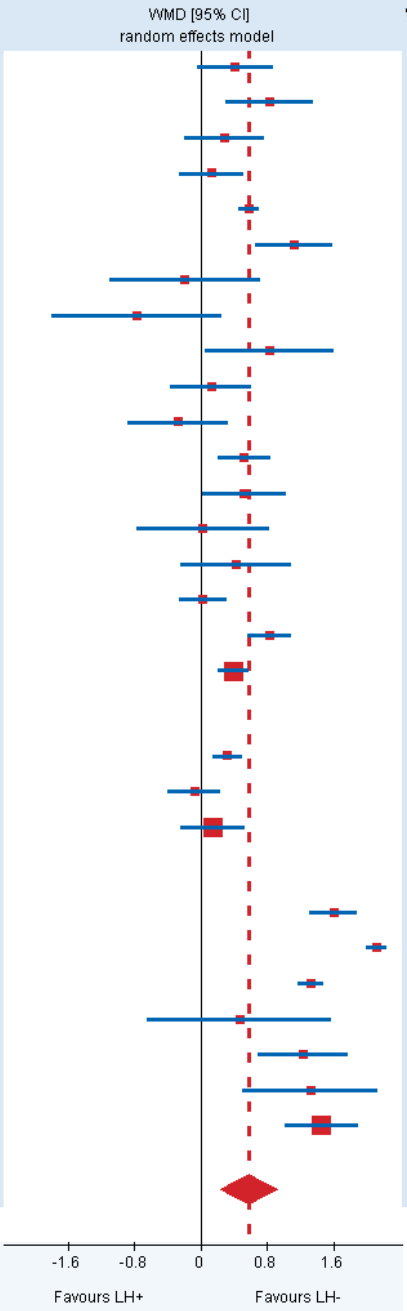

Test for heterogeneity: Q = 637.11, df = 24 (p < 0.0001), I² = 96.23%

Test overall effect: Z = 3.35 (p = 0.0008)

P-value for interaction: <0.0001

Figure 24. Subgroup analyses for uncontrolled glycemia – types of diabetes

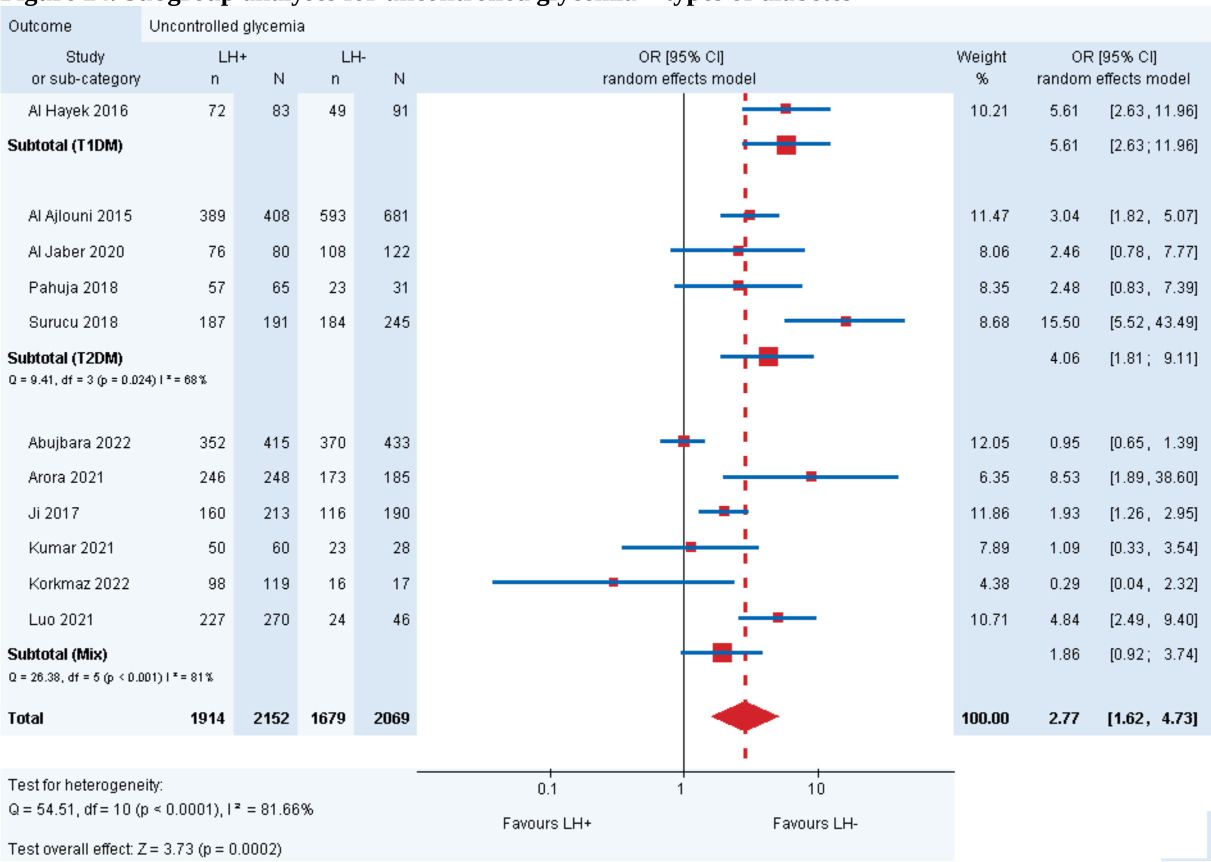

P-value for interaction: 0.187

Figure 25. Subgroup analyses for uncontrolled glycemia – % of patients with T1DM

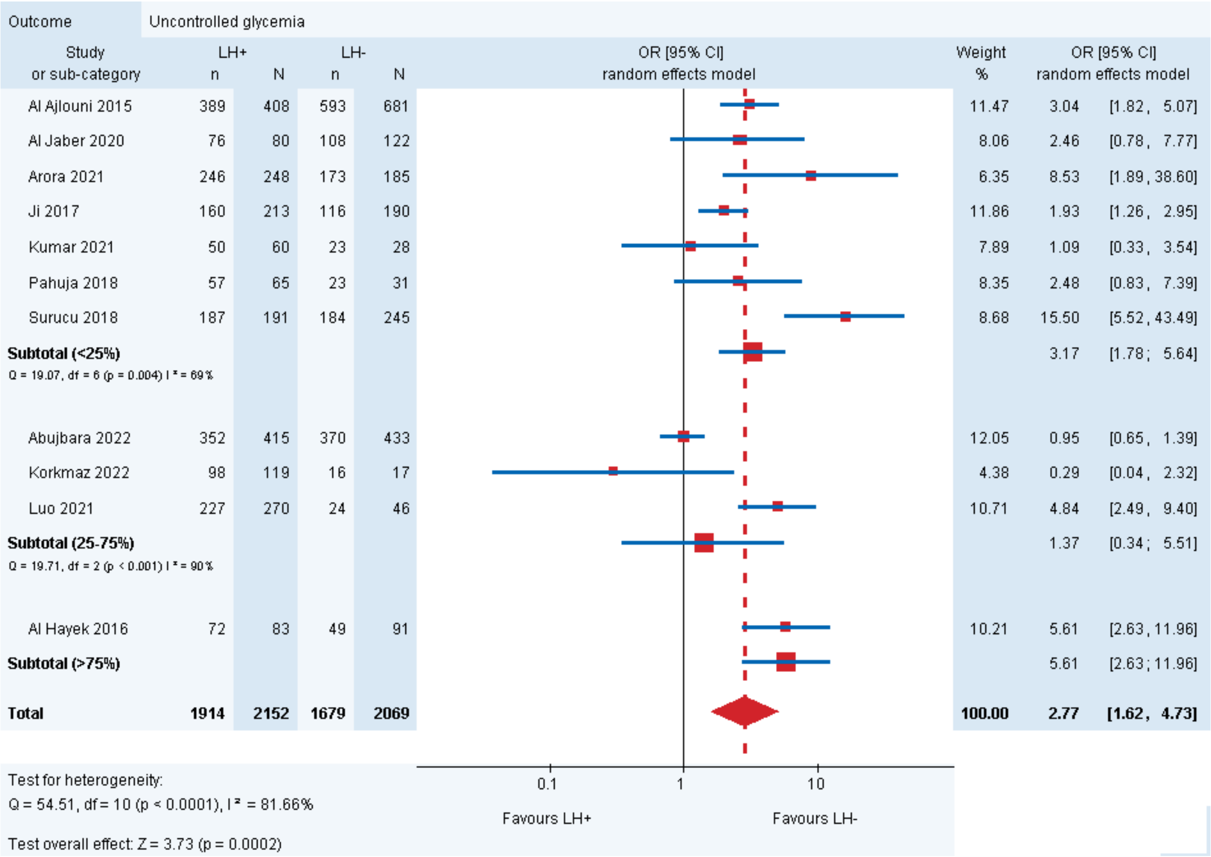

P-value for interaction: 0.094

Figure 26. Subgroup analyses for uncontrolled glycemia – geographical region (including Western vs Eastern Asia)

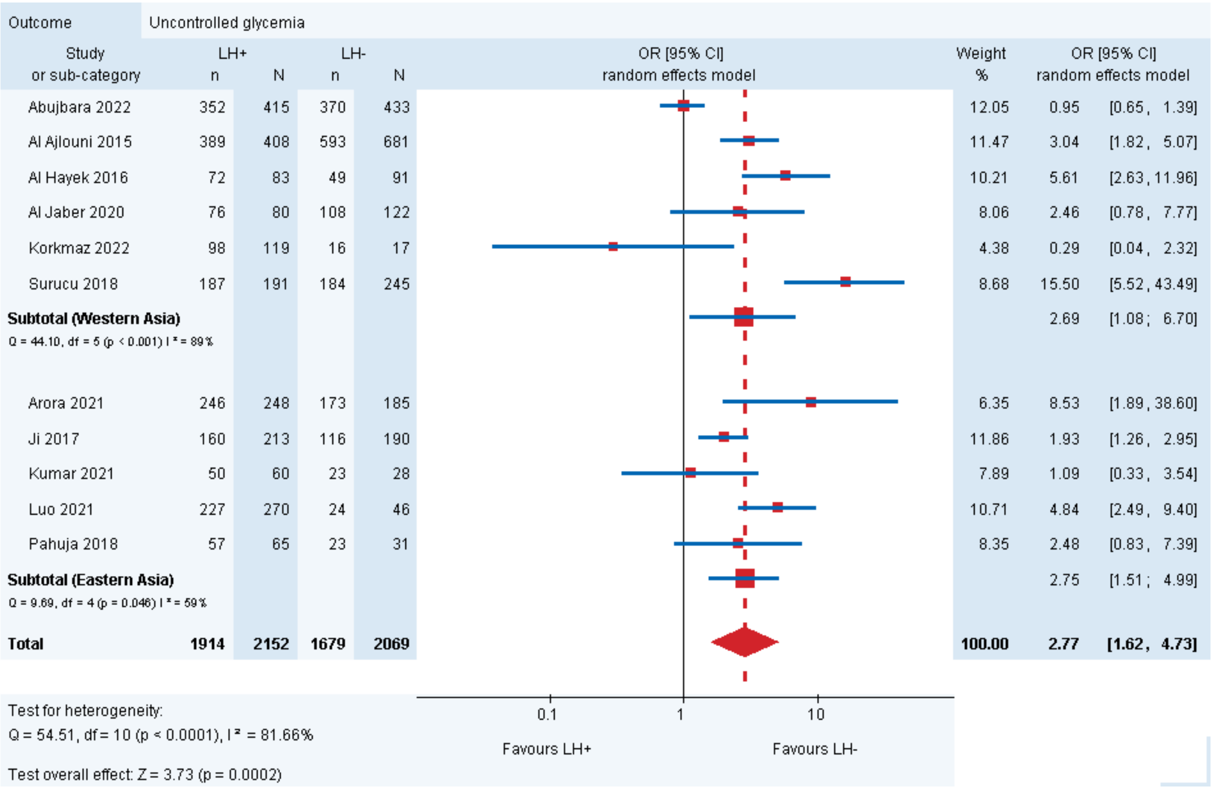

P-value for interaction: 0.968

Figure 27. Subgroup analyses for uncontrolled glycemia – duration of diabetes in years

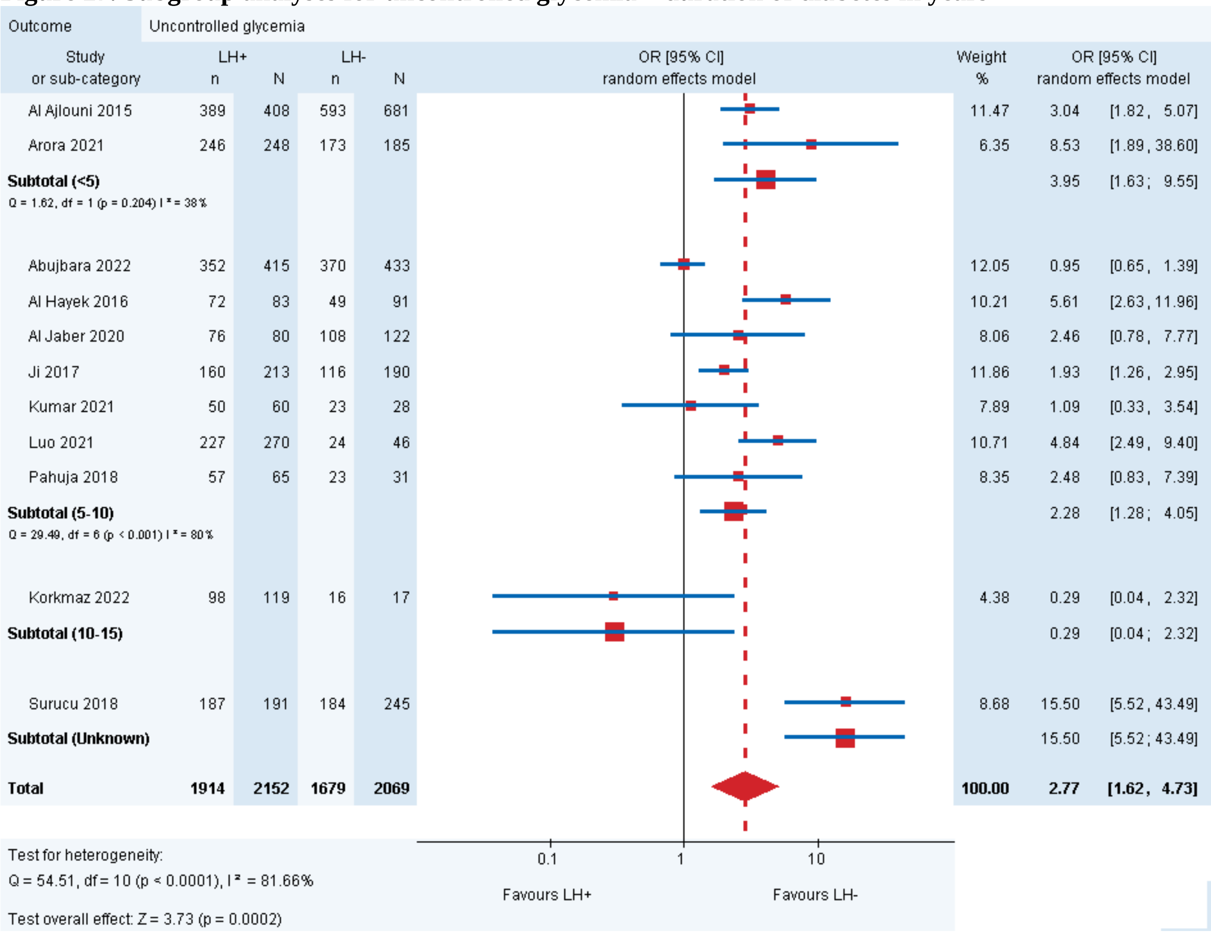

P-value for interaction: 0.001

Figure 28. Subgroup analyses for uncontrolled glycemia – a type of lipohypertrophy measurement

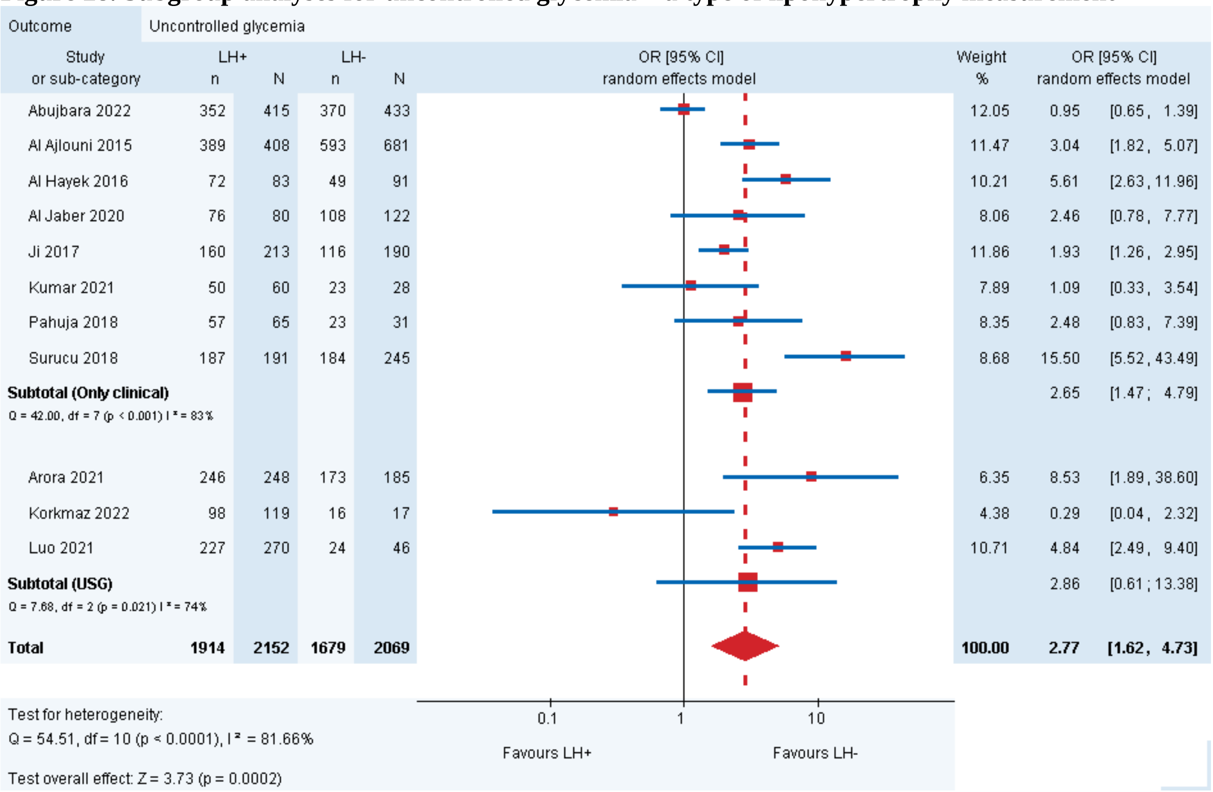

P-value for interaction: 0.928

Figure 29. Subgroup analyses for total daily insulin dose – types of diabetes

Outcome: Daily insulin dose

Study or sub-category	LH+ N	LH+ Mean	LH+ SD	LH- N	LH- Mean	LH- SD	Weight %	WMD [95% CI] random effects model	
Barola 2018	231	38.30	16.90	141	44.80	19.20	5.72	-6.50	[-10.35 , -2.65]
Gupta 2018	97	42.87	18.57	42	40.37	14.11	4.87	2.50	[-3.15 , 8.15]
**Subtotal (T1DM)**								-2.25	[-11.05 ; 6.56]
Q = 6.67, df = 1 (p = 0.010) I² = 85%									
Al-Jaber 2020	80	77.33	32.70	122	58.95	35.00	3.25	18.38	[8.90 , 27.86]
Baruah 2017	94	35.51	18.45	654	32.95	18.21	5.66	2.56	[-1.42 , 6.54]
Gentile 2020	718	54.90	8.20	509	42.30	10.00	6.64	12.60	[11.54 , 13.66]
Gentile 2022	487	65.50	10.60	673	53.60	9.50	6.62	11.90	[10.72 , 13.08]
Gunhan 2022	98	119.00	96.00	247	95.00	61.00	1.12	24.00	[3.53 , 44.47]
Pahuja 2019	64	43.97	x	29	33.55	x	3.45	10.42	[1.50 , 19.34]
**Subtotal (T2DM)**								10.81	[8.04 ; 13.58]
Q = 26.03, df = 5 (p < 0.001) I² = 81%									
Blanco 2013	277	56.00	26.90	153	41.00	24.10	5.20	15.00	[10.04 , 19.96]
Bochanen 2021	92	69.00	44.20	54	50.80	30.80	2.42	18.20	[5.99 , 30.41]
Cunningham 2013	28	54.30	33.10	27	44.90	18.40	2.00	9.40	[-4.69 , 23.49]
Frid 2016	2192	55.20	33.00	4889	45.10	31.50	6.52	10.10	[8.46 , 11.74]
Gentile 2019	169	35.50	8.80	127	32.80	9.70	6.38	2.70	[0.55 , 4.85]
Gentile 2021	360	49.00	24.00	420	43.00	27.00	5.83	6.00	[2.42 , 9.58]
Hauner 1996	66	49.00	16.00	213	45.00	15.00	5.49	4.00	[-0.35 , 8.35]
Kumar 2021	79	58.82	25.16	9	44.78	23.45	1.61	14.04	[-2.25 , 30.33]
Luo 2021	270	33.09	15.60	46	30.43	13.35	5.52	2.66	[-1.62 , 6.94]
Ji 2017	213	38.10	20.10	188	27.10	14.30	5.92	11.00	[7.61 , 14.39]
Pozzuoli 2017	151	48.80	25.90	202	37.80	21.40	5.14	11.00	[5.92 , 16.08]
Strollo 2016	298	49.00	18.00	89	41.00	19.00	5.44	8.00	[3.55 , 12.45]
Thewjitcharoen 2020	149	43.00	24.00	251	41.00	25.00	5.21	2.00	[-2.94 , 6.94]
**Subtotal (Mix)**								7.75	[5.17 ; 10.33]
Q = 60.09, df = 12 (p < 0.001) I² = 80%									
**Total**							**100.00**	**7.68**	**[5.31 , 10.06]**

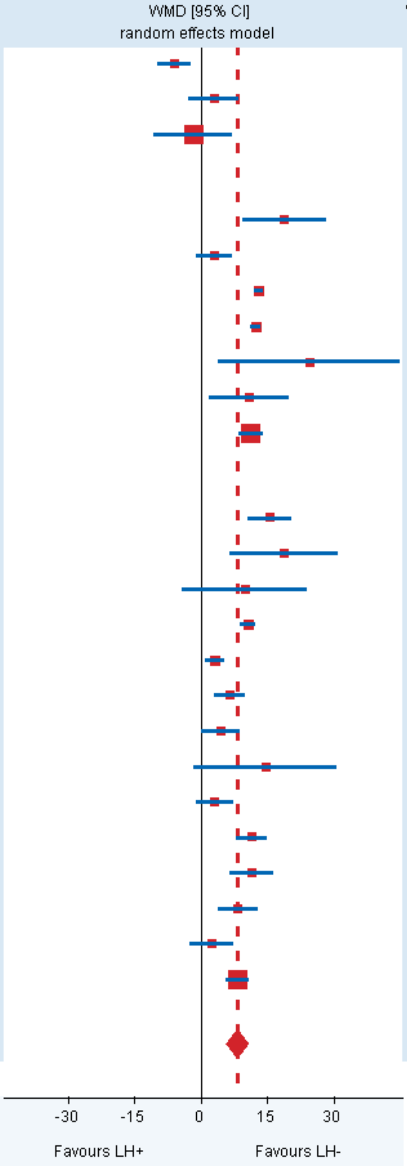

Test for heterogeneity: Q = 213.43, df = 20 (p < 0.0001), I² = 90.63%

Test overall effect: Z = 6.34 (p < 0.0001)

P-value for interaction: 0.013

Figure 30. Subgroup analyses for total daily insulin dose – % of patients with T1DM

Outcome: Daily insulin dose

Study or sub-category	LH+ N	LH+ Mean	LH+ SD	LH- N	LH- Mean	LH- SD	Weight %	WMD [95% CI] random effects model	
Al-Jaber 2020	80	77.33	32.70	122	58.95	35.00	3.25	18.38	[8.90 , 27.86]
Baruah 2017	94	35.51	18.45	654	32.95	18.21	5.66	2.56	[-1.42 , 6.54]
Gentile 2019	169	35.50	8.80	127	32.80	9.70	6.38	2.70	[0.55 , 4.85]
Gentile 2020	718	54.90	8.20	509	42.30	10.00	6.64	12.60	[11.54 , 13.66]
Gentile 2022	487	65.50	10.60	673	53.60	9.50	6.62	11.90	[10.72 , 13.08]
Gunhan 2022	98	119.00	96.00	247	95.00	61.00	1.12	24.00	[3.53 , 44.47]
Kumar 2021	79	58.82	25.16	9	44.78	23.45	1.61	14.04	[-2.25 , 30.33]
Ji 2017	213	38.10	20.10	188	27.10	14.30	5.92	11.00	[7.61 , 14.39]
Pahuja 2019	64	43.97	x	29	33.55	x	3.45	10.42	[1.50 , 19.34]
Pozzuoli 2017	151	48.80	25.90	202	37.80	21.40	5.14	11.00	[5.92 , 16.08]
Strollo 2016	298	49.00	18.00	89	41.00	19.00	5.44	8.00	[3.55 , 12.45]
Thewjitcharoen 2020	149	43.00	24.00	251	41.00	25.00	5.21	2.00	[-2.94 , 6.94]
**Subtotal (<25%)**								9.00	[6.20 ; 11.81]
Q = 103.49, df = 11 (p < 0.001) I² = 89%									
Blanco 2013	277	56.00	26.90	153	41.00	24.10	5.20	15.00	[10.04 , 19.96]
Bochanen 2021	92	69.00	44.20	54	50.80	30.80	2.42	18.20	[5.99 , 30.41]
Cunningham 2013	28	54.30	33.10	27	44.90	18.40	2.00	9.40	[-4.69 , 23.49]
Frid 2016	2192	55.20	33.00	4889	45.10	31.50	6.52	10.10	[8.46 , 11.74]
Gentile 2021	360	49.00	24.00	420	43.00	27.00	5.83	6.00	[2.42 , 9.58]
Luo 2021	270	33.09	15.60	46	30.43	13.35	5.52	2.66	[-1.62 , 6.94]
**Subtotal (25-75%)**								9.15	[5.32 ; 12.97]
Q = 20.52, df = 5 (p < 0.001) I² = 76%									
Barola 2018	231	38.30	16.90	141	44.80	19.20	5.72	-6.50	[-10.35 , -2.65]
Gupta 2018	97	42.87	18.57	42	40.37	14.11	4.87	2.50	[-3.15 , 8.15]
Hauner 1996	66	49.00	16.00	213	45.00	15.00	5.49	4.00	[-0.35 , 8.35]
**Subtotal (>75%)**								-0.13	[-7.19 ; 6.93]
Q = 14.40, df = 2 (p < 0.001) I² = 86%									
**Total**							**100.00**	**7.68**	**[5.31 , 10.06]**

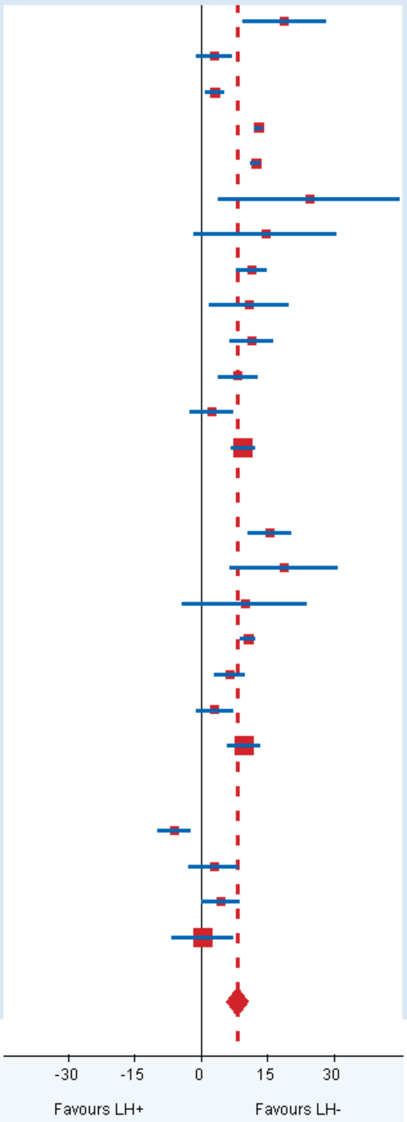

Test for heterogeneity: Q = 213.43, df = 20 (p < 0.0001), I² = 90.63%

Test overall effect: Z = 6.34 (p < 0.0001)

P-value for interaction: 0.052

Figure 31. Subgroup analyses for total daily insulin dose – geographical region (general)

Outcome: Daily insulin dose

Study or sub-category	LH+ N	LH+ Mean	LH+ SD	LH- N	LH- Mean	LH- SD	Weight %	WMD [95% CI] random effects model
Al-Jaber 2020	80	77.33	32.70	122	58.95	35.00	3.25	18.38 [8.90 , 27.86]
Barola 2018	231	38.30	16.90	141	44.80	19.20	5.72	-6.50 [-10.35 , -2.65]
Baruah 2017	94	35.51	18.45	654	32.95	18.21	5.66	2.56 [-1.42 , 6.54]
Gunhan 2022	98	119.00	96.00	247	95.00	61.00	1.12	24.00 [3.53 , 44.47]
Gupta 2018	97	42.87	18.57	42	40.37	14.11	4.87	2.50 [-3.15 , 8.15]
Kumar 2021	79	58.82	25.16	9	44.78	23.45	1.61	14.04 [-2.25 , 30.33]
Luo 2021	270	33.09	15.60	46	30.43	13.35	5.52	2.66 [-1.62 , 6.94]
Ji 2017	213	38.10	20.10	188	27.10	14.30	5.92	11.00 [7.61 , 14.39]
Pahuja 2019	64	43.97	x	29	33.55	x	3.45	10.42 [1.50 , 19.34]
Thewjitcharoen 2020	149	43.00	24.00	251	41.00	25.00	5.21	2.00 [-2.94 , 6.94]
**Subtotal (Asia)**								6.00 [1.21 ; 10.80]
Q = 62.73, df = 9 (p < 0.001) I² = 86%								
Blanco 2013	277	56.00	26.90	153	41.00	24.10	5.20	15.00 [10.04 , 19.96]
Bochanen 2021	92	69.00	44.20	54	50.80	30.80	2.42	18.20 [5.99 , 30.41]
Cunningham 2013	28	54.30	33.10	27	44.90	18.40	2.00	9.40 [-4.69 , 23.49]
Gentile 2019	169	35.50	8.80	127	32.80	9.70	6.38	2.70 [0.55 , 4.85]
Gentile 2020	718	54.90	8.20	509	42.30	10.00	6.64	12.60 [11.54 , 13.66]
Gentile 2021	360	49.00	24.00	420	43.00	27.00	5.83	6.00 [2.42 , 9.58]
Gentile 2022	487	65.50	10.60	673	53.60	9.50	6.62	11.90 [10.72 , 13.08]
Hauner 1996	66	49.00	16.00	213	45.00	15.00	5.49	4.00 [-0.35 , 8.35]
Pozzuoli 2017	151	48.80	25.90	202	37.80	21.40	5.14	11.00 [5.92 , 16.08]
Strollo 2016	298	49.00	18.00	89	41.00	19.00	5.44	8.00 [3.55 , 12.45]
**Subtotal (Europe)**								9.25 [6.42 ; 12.09]
Q = 91.21, df = 9 (p < 0.001) I² = 90%								
Frid 2016	2192	55.20	33.00	4889	45.10	31.50	6.52	10.10 [8.46 , 11.74]
**Subtotal (Worldwide)**								10.10 [8.46 ; 11.74]
**Total**							**100.00**	**7.68 [5.31 , 10.06]**

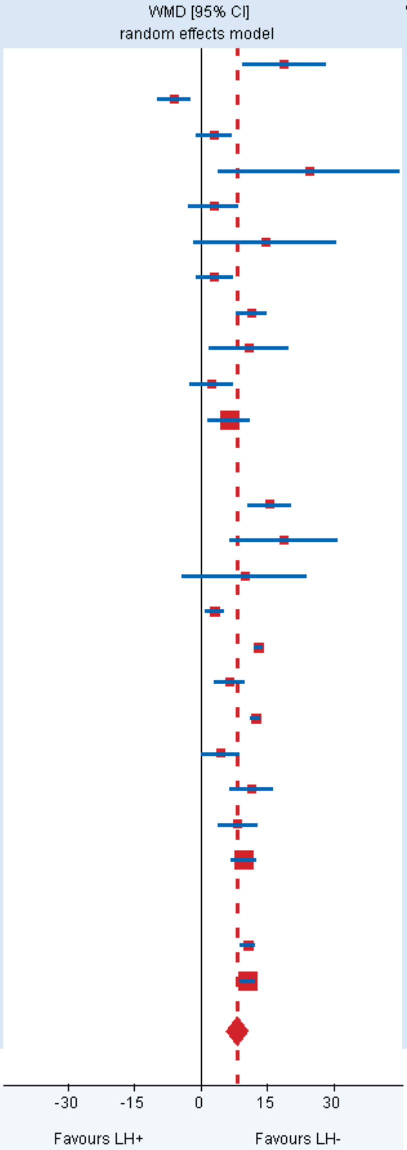

Test for heterogeneity: Q = 213.43, df = 20 (p < 0.0001), I² = 90.63%

Test overall effect: Z = 6.34 (p < 0.0001)

P-value for interaction: 0.276

Figure 32. Subgroup analyses for total daily insulin dose – geographical region (including Western vs Eastern Asia)

Outcome	Daily insulin dose								
Study or sub-category	LH+ N	LH+ Mean	LH+ SD	LH- N	LH- Mean	LH- SD	Weight %	WMD [95% CI] random effects model	
Blanco 2013	277	56.00	26.90	153	41.00	24.10	5.20	15.00	[10.04 , 19.96]
Bochanen 2021	92	69.00	44.20	54	50.80	30.80	2.42	18.20	[5.99 , 30.41]
Cunningham 2013	28	54.30	33.10	27	44.90	18.40	2.00	9.40	[-4.69 , 23.49]
Gentile 2019	169	35.50	8.80	127	32.80	9.70	6.38	2.70	[0.55 , 4.85]
Gentile 2020	718	54.90	8.20	509	42.30	10.00	6.64	12.60	[11.54 , 13.66]
Gentile 2021	360	49.00	24.00	420	43.00	27.00	5.83	6.00	[2.42 , 9.58]
Gentile 2022	487	65.50	10.60	673	53.60	9.50	6.62	11.90	[10.72 , 13.08]
Hauner 1996	66	49.00	16.00	213	45.00	15.00	5.49	4.00	[-0.35 , 8.35]
Pozzuoli 2017	151	48.80	25.90	202	37.80	21.40	5.14	11.00	[5.92 , 16.08]
Strollo 2016	298	49.00	18.00	89	41.00	19.00	5.44	8.00	[3.55 , 12.45]
**Subtotal (Europe)**								9.25	[6.42 ; 12.09]
Q = 91.21, df = 9 (p < 0.001) I² = 90%									
Frid 2016	2192	55.20	33.00	4889	45.10	31.50	6.52	10.10	[8.46 , 11.74]
**Subtotal (Worldwide)**								10.10	[8.46 ; 11.74]
Al-Jaber 2020	80	77.33	32.70	122	58.95	35.00	3.25	18.38	[8.90 , 27.86]
Gunhan 2022	98	119.00	96.00	247	95.00	61.00	1.12	24.00	[3.53 , 44.47]
**Subtotal (Western Asia)**								19.37	[10.77 ; 27.98]
Q = 0.24, df = 1 (p = 0.625) I² = 0%									
Barola 2018	231	38.30	16.90	141	44.80	19.20	5.72	-6.50	[-10.35 , -2.65]
Baruah 2017	94	35.51	18.45	654	32.95	18.21	5.66	2.56	[-1.42 , 6.54]
Gupta 2018	97	42.87	18.57	42	40.37	14.11	4.87	2.50	[-3.15 , 8.15]
Kumar 2021	79	58.82	25.16	9	44.78	23.45	1.61	14.04	[-2.25 , 30.33]
Luo 2021	270	33.09	15.60	46	30.43	13.35	5.52	2.66	[-1.62 , 6.94]
Ji 2017	213	38.10	20.10	188	27.10	14.30	5.92	11.00	[7.61 , 14.39]
Pahuja 2019	64	43.97	x	29	33.55	x	3.45	10.42	[1.50 , 19.34]
Thewjitcharoen 2020	149	43.00	24.00	251	41.00	25.00	5.21	2.00	[-2.94 , 6.94]
**Subtotal (Eastern Asia)**								3.87	[-0.88 ; 8.62]
Q = 49.48, df = 7 (p < 0.001) I² = 86%									
**Total**							**100.00**	**7.68**	**[5.31 , 10.06]**

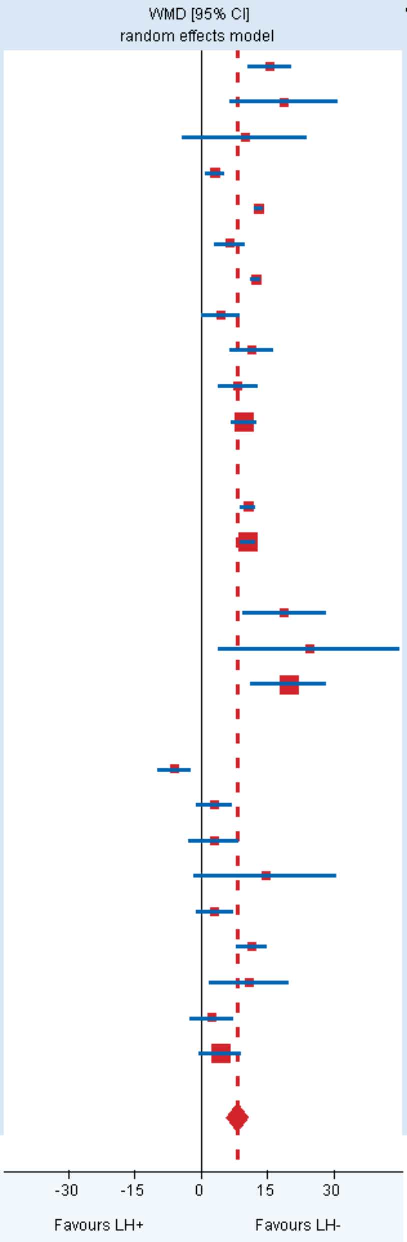

Test for heterogeneity: Q = 213.43, df = 20 (p < 0.0001), I² = 90.63%

Test overall effect: Z = 6.34 (p < 0.0001)

P-value for interaction: 0.012

Figure 33. Subgroup analyses for total daily insulin dose – duration of insulin therapy

Outcome: Daily insulin dose

Study or sub-category	LH+ N	LH+ Mean	LH+ SD	LH- N	LH- Mean	LH- SD	Weight %	WMD [95% CI] random effects model
Baruah 2017	94	35.51	18.45	654	32.95	18.21	5.66	2.56 [-1.42 , 6.54]
Blanco 2013	277	56.00	26.90	153	41.00	24.10	5.20	15.00 [10.04 , 19.96]
Gentile 2019	169	35.50	8.80	127	32.80	9.70	6.38	2.70 [0.55 , 4.85]
**Subtotal (<5)**								6.47 [-0.22 ; 13.16]
Q = 20.73, df = 2 (p < 0.001) I² = 90%								
Al-Jaber 2020	80	77.33	32.70	122	58.95	35.00	3.25	18.38 [8.90 , 27.86]
Frid 2016	2192	55.20	33.00	4889	45.10	31.50	6.52	10.10 [8.46 , 11.74]
Gentile 2020	718	54.90	8.20	509	42.30	10.00	6.64	12.60 [11.54 , 13.66]
Gentile 2022	487	65.50	10.60	673	53.60	9.50	6.62	11.90 [10.72 , 13.08]
Gunhan 2022	98	119.00	96.00	247	95.00	61.00	1.12	24.00 [3.53 , 44.47]
Gupta 2018	97	42.87	18.57	42	40.37	14.11	4.87	2.50 [-3.15 , 8.15]
Kumar 2021	79	58.82	25.16	9	44.78	23.45	1.61	14.04 [-2.25 , 30.33]
Luo 2021	270	33.09	15.60	46	30.43	13.35	5.52	2.66 [-1.62 , 6.94]
Ji 2017	213	38.10	20.10	188	27.10	14.30	5.92	11.00 [7.61 , 14.39]
Pahuja 2019	64	43.97	x	29	33.55	x	3.45	10.42 [1.50 , 19.34]
Pozzuoli 2017	151	48.80	25.90	202	37.80	21.40	5.14	11.00 [5.92 , 16.08]
**Subtotal (5-10)**								10.26 [8.40 ; 12.12]
Q = 37.26, df = 10 (p < 0.001) I² = 73%								
Cunningham 2013	28	54.30	33.10	27	44.90	18.40	2.00	9.40 [-4.69 , 23.49]
Gentile 2021	360	49.00	24.00	420	43.00	27.00	5.83	6.00 [2.42 , 9.58]
Strollo 2016	298	49.00	18.00	89	41.00	19.00	5.44	8.00 [3.55 , 12.45]
Thewjitcharoen 2020	149	43.00	24.00	251	41.00	25.00	5.21	2.00 [-2.94 , 6.94]
**Subtotal (10-15)**								5.72 [3.06 ; 8.38]
Q = 3.47, df = 3 (p = 0.324) I² = 14%								
Barola 2018	231	38.30	16.90	141	44.80	19.20	5.72	-6.50 [-10.35 , -2.65]
Bochanen 2021	92	69.00	44.20	54	50.80	30.80	2.42	18.20 [5.99 , 30.41]
Hauner 1996	66	49.00	16.00	213	45.00	15.00	5.49	4.00 [-0.35 , 8.35]
**Subtotal (Unknown)**								3.78 [-6.98 ; 14.55]
Q = 22.41, df = 2 (p < 0.001) I² = 91%								
**Total**							**100.00**	**7.68 [5.31 , 10.06]**

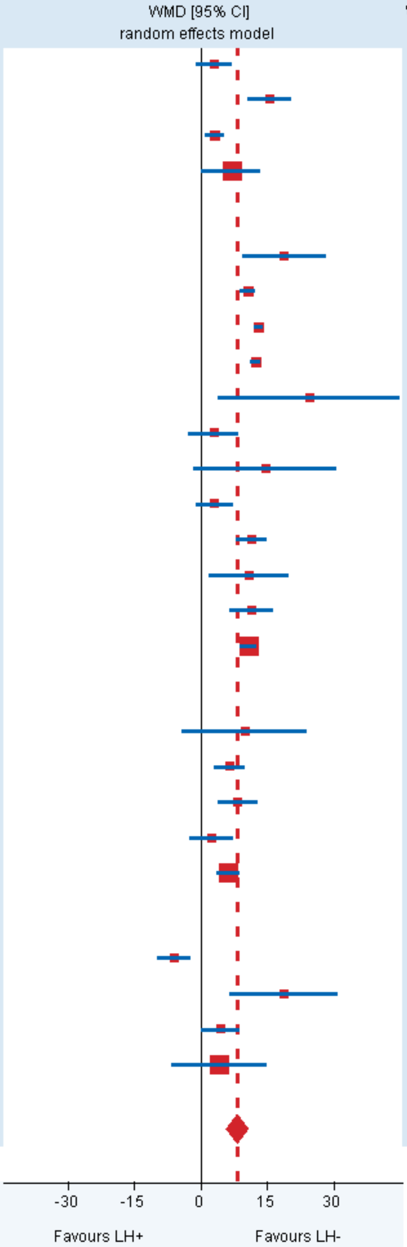

Test for heterogeneity: Q = 213.43, df = 20 (p < 0.0001), I² = 90.63%

Test overall effect: Z = 6.34 (p < 0.0001)

P-value for interaction: 0.033

Figure 34. Subgroup analyses for total daily insulin dose – a type of lipohypertrophy measurement

Outcome: Daily insulin dose

Study or sub-category	LH+ N	LH+ Mean	LH+ SD	LH- N	LH- Mean	LH- SD	Weight %	WMD [95% CI] random effects model
Al-Jaber 2020	80	77.33	32.70	122	58.95	35.00	3.25	18.38 [8.90 , 27.86]
Barola 2018	231	38.30	16.90	141	44.80	19.20	5.72	-6.50 [-10.35 , -2.65]
Baruah 2017	94	35.51	18.45	654	32.95	18.21	5.66	2.56 [-1.42 , 6.54]
Bochanen 2021	92	69.00	44.20	54	50.80	30.80	2.42	18.20 [5.99 , 30.41]
Cunningham 2013	28	54.30	33.10	27	44.90	18.40	2.00	9.40 [-4.69 , 23.49]
Frid 2016	2192	55.20	33.00	4889	45.10	31.50	6.52	10.10 [8.46 , 11.74]
Gentile 2019	169	35.50	8.80	127	32.80	9.70	6.38	2.70 [0.55 , 4.85]
Gunhan 2022	98	119.00	96.00	247	95.00	61.00	1.12	24.00 [3.53 , 44.47]
Gupta 2018	97	42.87	18.57	42	40.37	14.11	4.87	2.50 [-3.15 , 8.15]
Hauner 1996	66	49.00	16.00	213	45.00	15.00	5.49	4.00 [-0.35 , 8.35]
Ji 2017	213	38.10	20.10	188	27.10	14.30	5.92	11.00 [7.61 , 14.39]
Pahuja 2019	64	43.97	x	29	33.55	x	3.45	10.42 [1.50 , 19.34]
Pozzuoli 2017	151	48.80	25.90	202	37.80	21.40	5.14	11.00 [5.92 , 16.08]
Strollo 2016	298	49.00	18.00	89	41.00	19.00	5.44	8.00 [3.55 , 12.45]
**Subtotal (Only clinical)** Q = 104.88, df = 13 (p < 0.001) I ² = 88 %								7.24 [3.88 ; 10.59]
Gentile 2020	718	54.90	8.20	509	42.30	10.00	6.64	12.60 [11.54 , 13.66]
Gentile 2022	487	65.50	10.60	673	53.60	9.50	6.62	11.90 [10.72 , 13.08]
Kumar 2021	79	58.82	25.16	9	44.78	23.45	1.61	14.04 [-2.25 , 30.33]
Luo 2021	270	33.09	15.60	46	30.43	13.35	5.52	2.66 [-1.62 , 6.94]
**Subtotal (USG)** Q = 19.59, df = 3 (p < 0.001) I ² = 85 %								10.31 [7.54 ; 13.09]
Blanco 2013	277	56.00	26.90	153	41.00	24.10	5.20	15.00 [10.04 , 19.96]
Gentile 2021	360	49.00	24.00	420	43.00	27.00	5.83	6.00 [2.42 , 9.58]
Thewjitcharoen 2020	149	43.00	24.00	251	41.00	25.00	5.21	2.00 [-2.94 , 6.94]
**Subtotal (Partial USG)** Q = 14.18, df = 2 (p < 0.001) I ² = 86 %								7.62 [0.75 ; 14.48]
**Total**							**100.00**	**7.68 [5.31 , 10.06]**

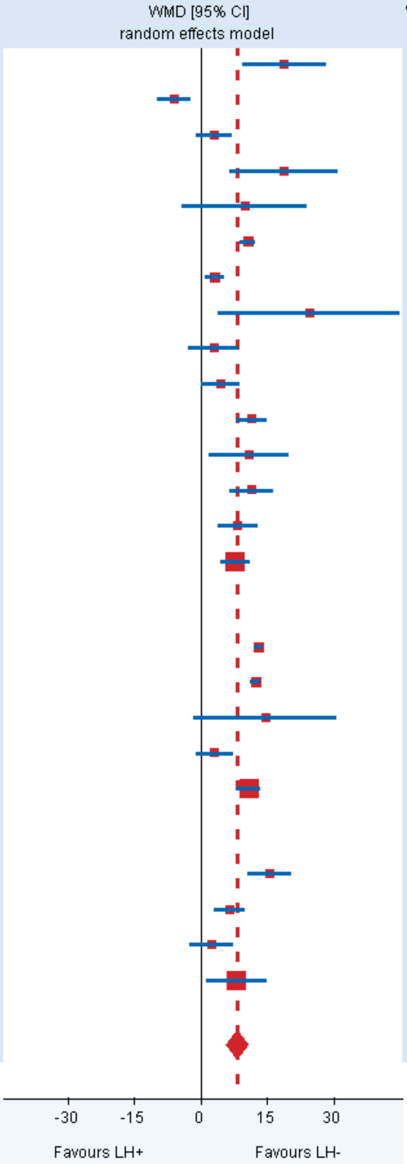

Test for heterogeneity: Q = 213.43, df = 20 (p < 0.0001), I ² = 90.63%

Test overall effect: Z = 6.34 (p < 0.0001)

P-value for interaction: 0.356

Figure 35. Sensitivity analysis for unexplained hypoglycemia (studies published between 2014–2023)

Outcome: Unexplained hypoglycemia

Study or sub-category	LH+ n	LH+ N	LH- n	LH- N	Weight %	OR [95% CI] random effects model
Al Hayek 2016	28	83	12	91	11.47	3.35 [1.57, 7.16]
Bochanen 2021	51	92	17	54	11.62	2.71 [1.34, 5.49]
Gupta 2018	93	97	23	42	10.11	19.21 [5.96, 61.94]
Gentile 2020	595	718	109	509	12.52	17.75 [13.32, 23.66]
Gentile 2021	157	360	60	420	12.44	4.64 [3.29, 6.54]
Strollo 2016	290	298	27	89	11.24	83.24 [36.11, 191.90]
Surucu 2018	80	191	26	245	12.15	6.07 [3.69, 9.99]
Thewjitcharoen 2020	4	149	1	251	6.66	6.90 [0.76, 62.29]
Tsadik 2018	29	103	27	73	11.80	0.67 [0.35, 1.27]
**Total**	**1327**	**2091**	**302**	**1774**	**100.00**	**6.69 [2.94, 15.23]**

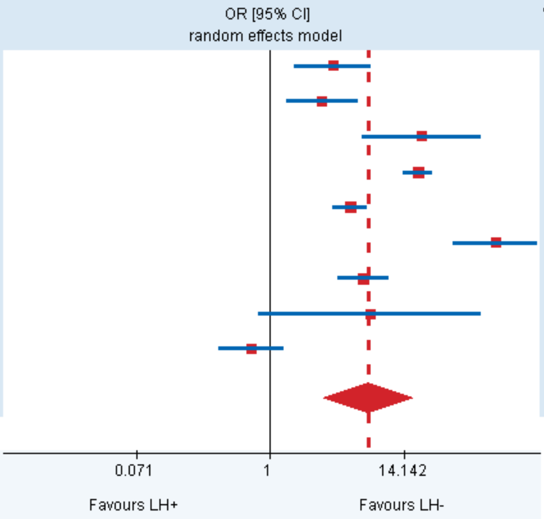

Test for heterogeneity:
Q = 144.35, df = 8 (p < 0.0001), I² = 94.46%

Test overall effect: Z = 4.53 (p < 0.0001)

Figure 36. Sensitivity analysis for HbA1c (%) (studies published between 2014–2023)

Outcome: HbA1c [%]

Study or sub-category	LH+ N	LH+ Mean	LH+ SD	LH- N	LH- Mean	LH- SD	Weight %	WMD [95% CI] random effects model
Al Jaber 2020	80	9.53	1.60	122	9.14	1.70	4.47	0.39 [-0.07, 0.85]
Arora 2021	290	10.53	1.73	210	8.95	1.53	4.72	1.58 [1.29, 1.86]
Barola 2018	231	10.00	2.70	141	9.20	2.40	4.36	0.80 [0.27, 1.33]
Baruah 2017	94	9.34	2.28	654	9.08	2.10	4.43	0.26 [-0.23, 0.75]
Bochanen 2021	92	7.50	1.10	54	7.40	1.20	4.58	0.10 [-0.29, 0.49]
Frid 2016	2205	8.85	2.70	4795	8.30	1.90	4.85	0.55 [0.43, 0.67]
Gentile 2019	169	9.20	1.60	127	8.10	2.30	4.46	1.10 [0.63, 1.57]
Gentile 2020	718	9.60	1.10	509	7.50	1.10	4.85	2.10 [1.98, 2.22]
Gentile 2021	360	8.00	1.20	420	7.70	1.30	4.82	0.30 [0.12, 0.48]
Gentile 2022	487	8.60	1.60	673	7.30	1.00	4.83	1.30 [1.14, 1.46]
Gupta 2018	97	9.62	2.13	42	9.84	2.66	3.59	-0.22 [-1.13, 0.69]
Gunhan 2022	98	8.50	1.70	247	9.30	7.80	3.35	-0.80 [-1.83, 0.23]
Hajheydari 2011	35	9.50	2.20	185	8.70	1.90	3.87	0.80 [0.02, 1.58]
Ji 2014	134	7.87	1.53	246	8.17	4.41	4.21	-0.30 [-0.91, 0.31]
Ji 2017	213	8.20	1.80	188	7.70	1.50	4.67	0.50 [0.18, 0.82]
Kamrul-Hasan 2020	78	10.00	2.20	769	9.50	2.00	4.39	0.50 [-0.01, 1.01]
Kumar 2021	79	9.12	2.53	8	8.68	1.39	3.18	0.44 [-0.67, 1.55]
Luo 2021	270	8.63	1.76	46	7.42	1.73	4.33	1.21 [0.67, 1.75]
Pahuja 2019	65	8.30	x	31	7.90	x	4.11	0.40 [-0.26, 1.06]
Pozzouli 2018	151	7.70	1.40	202	7.70	1.30	4.72	0.00 [-0.29, 0.29]
Singha 2021	46	9.00	2.60	45	7.70	1.10	3.79	1.30 [0.48, 2.12]
Strollo 2016	298	8.30	1.20	89	7.50	1.10	4.74	0.80 [0.53, 1.07]
Thewjitcharoen 2020	149	7.80	1.50	251	7.90	1.70	4.68	-0.10 [-0.42, 0.22]
**Total**							**100.00**	**0.59 [0.26, 0.93]**

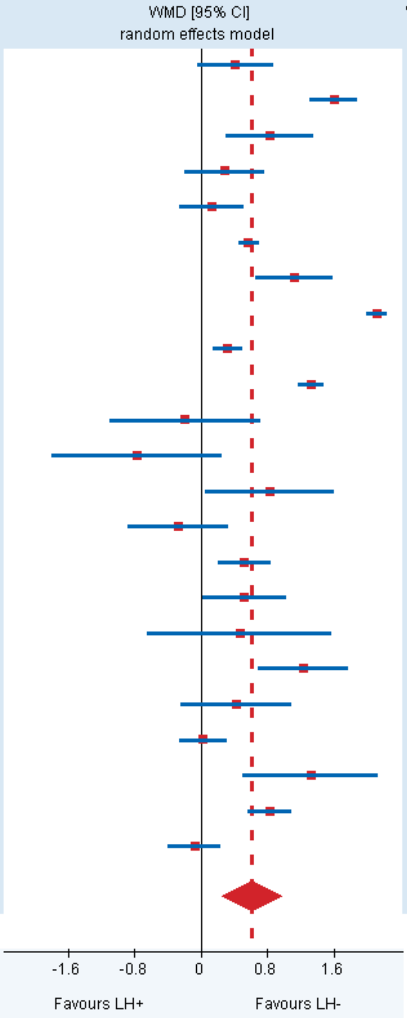

Test for heterogeneity: Q = 620.51, df = 22 (p < 0.0001), I² = 96.45%

Test overall effect: Z = 3.46 (p = 0.0005)

Figure 37. Sensitivity analysis for total daily insulin dose (studies published between 2014–2023)

Outcome: Daily insulin dose

Study or sub-category	LH+ N	LH+ Mean	LH+ SD	LH- N	LH- Mean	LH- SD	Weight %	WMD [95% CI] random effects model
Al-Jaber 2020	80	77.33	32.70	122	58.95	35.00	3.73	18.38 [8.90 , 27.86]
Barola 2018	231	38.30	16.90	141	44.80	19.20	6.55	-6.50 [-10.35 , -2.65]
Baruah 2017	94	35.51	18.45	654	32.95	18.21	6.48	2.56 [-1.42 , 6.54]
Bochanen 2021	92	69.00	44.20	54	50.80	30.80	2.78	18.20 [5.99 , 30.41]
Frid 2016	2192	55.20	33.00	4889	45.10	31.50	7.45	10.10 [8.46 , 11.74]
Gentile 2019	169	35.50	8.80	127	32.80	9.70	7.29	2.70 [0.55 , 4.85]
Gentile 2020	718	54.90	8.20	509	42.30	10.00	7.59	12.60 [11.54 , 13.66]
Gentile 2021	360	49.00	24.00	420	43.00	27.00	6.68	6.00 [2.42 , 9.58]
Gentile 2022	487	65.50	10.60	673	53.60	9.50	7.56	11.90 [10.72 , 13.08]
Gunhan 2022	98	119.00	96.00	247	95.00	61.00	1.29	24.00 [3.53 , 44.47]
Gupta 2018	97	42.87	18.57	42	40.37	14.11	5.59	2.50 [-3.15 , 8.15]
Kumar 2021	79	58.82	25.16	9	44.78	23.45	1.86	14.04 [-2.25 , 30.33]
Luo 2021	270	33.09	15.60	46	30.43	13.35	6.32	2.66 [-1.62 , 6.94]
Ji 2017	213	38.10	20.10	188	27.10	14.30	6.77	11.00 [7.61 , 14.39]
Pahuja 2019	64	43.97	x	29	33.55	x	3.96	10.42 [1.50 , 19.34]
Pozzuoli 2017	151	48.80	25.90	202	37.80	21.40	5.89	11.00 [5.92 , 16.08]
Strollo 2016	298	49.00	18.00	89	41.00	19.00	6.23	8.00 [3.55 , 12.45]
Thewjitcharoen 2020	149	43.00	24.00	251	41.00	25.00	5.97	2.00 [-2.94 , 6.94]
**Total**							**100.00**	**7.44 [4.89 , 9.99]**

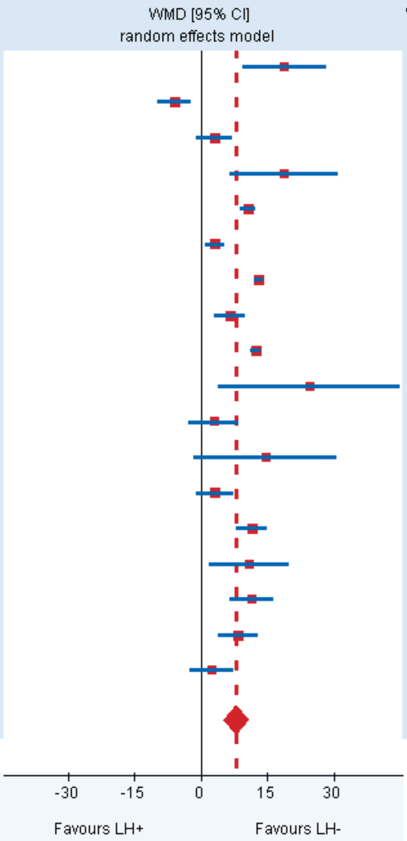

Test for heterogeneity: Q = 202.39, df = 17 (p < 0.0001), I² = 91.60%

Test overall effect: Z = 5.72 (p < 0.0001)

Figure 38. Egger plot for publication bias – unexplained hypoglycemia

Outcome	Unexplained hypoglycemia

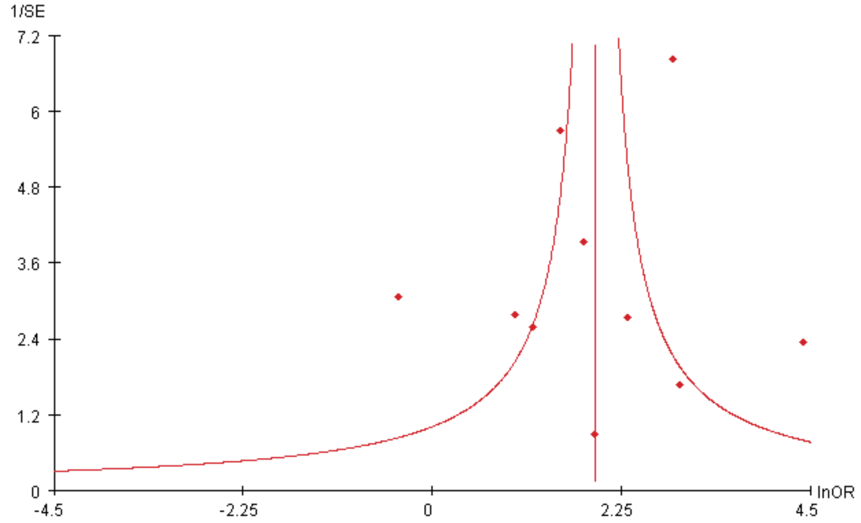

Kendall: 0.2000

Egger: -1.3201 [-7.9163, 5.2761] (p = 0.6567)

Horbold-Egger: -1.7009 [-8.7220, 5.3203] (p = 0.5917)

Figure 39. Egger plot for publication bias – uncontrolled glycemia (HbA1c >7%)

Outcome	Uncontrolled glycemia

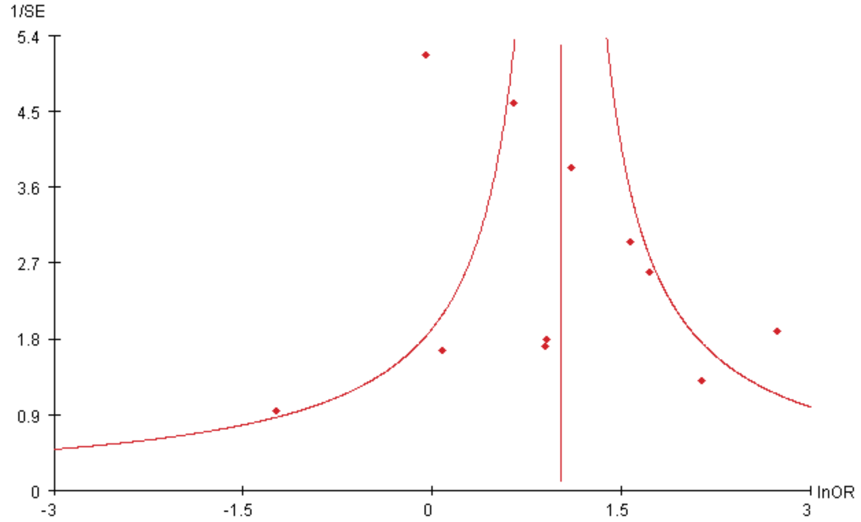

Kendall: -0.0182

Egger: 1.8074 [-1.5743, 5.1892] (p = 0.2575)

Horbold-Egger: 1.0871 [-3.0201, 5.1943] (p = 0.5641)
